# Supplementary material for: Lifecourse investigation of the cumulative impact of adversity on cognitive function in old age and the mediating role of mental health: longitudinal birth cohort study
Source: BMJ Open. 2023 Nov 8;13(11):e074105. doi: 10.1136/bmjopen-2023-074105 (PMC10632868; doi:10.1136/bmjopen-2023-074105)
Supplement: Supplementary data [file bmjopen-2023-074105supp001.pdf]

Supplementary materials

Table S1. List of adversity indicators at each stage of the life course.

Adversity variables	N (%)
**Childhood (<17 years)**	
Economic adversity	1353 (63.6%)
Overcrowding	*376 (17.7%)*
Lack of household amenities	*1095 (52.6%)*
Adverse housing condition	*518 (28.9%)*
Paternal occupational status	*167 (8.9%)*
Psychosocial adversity	324 (15.2%)
Separation from mother (>28 days; not due to hospitalisation)	*98 (5.1%)*
Parental divorce	*118 (5.5%)*
Negative peer relationships	*129 (6.8%)*
**Young adulthood (20-36 years)**	
Economic adversity	823 (40.3%)
Overcrowding	*24 (1.3%)*
Lack of household amenities	*185 (9.6%)*
Financial hardships	*323 (20.9%)*
Unemployment	*387 (19.9%)*
Psychosocial adversity	648 (31.4%)
Social isolation	*569 (29.3%)*
Divorce/separation	*112 (5.5%)*
**Mid-adulthood (43-53 years)**	
Economic adversity	993 (47.4%)
Financial hardships	*573 (27.4%)*
Work-related crisis	*358 (17.1%)*
Adverse living conditions	*410 (20.3%)*
Psychosocial adversity	1105 (52.7%)
Social isolation	*148 (7.1%)*
Lack of social support	*207 (9.9%)*
Loss of contact with relatives or friends	*269 (12.8%)*
Difficulties with own children	*563 (29.7%)*
Divorce/separation	*355 (16.9%)*
**Late adulthood (63 years)**	
Economic adversity	316 (16.7%)
Financial hardships	*234 (12.7%)*
Work-related crisis	*113 (6.1%)*
Psychosocial adversity	511 (25.8%)
Social isolation	*50 (2.8%)*
Lack of social support	*12 (0.7%)*
Loss of contact with relatives or friends	*99 (5.4%)*
Difficulties with own children	*217 (13.0%)*
Divorce/separation	*209 (11.5%)*

Liu Y, *et al. BMJ Open* 2023; 13:e074105. doi: 10.1136/bmjopen-2023-074105

Causal mediation analysis: Statistical equations

Defining total effect, average direct effect (ADE) and average causal mediation effect (ACME) (adapted from Imai et al., 2011):

Let t = exposure, M = mediator, and Y = outcome. The ACME can be defined using the below formula:

$$\sum\{Y(t, M(t_1)) - Y(t, M(t_0))\}$$

Where ACME equals the difference in outcome due to the change in mediator from the value expected under no exposure (M (t_0)) to the value expected under exposure (M (t_1)), holding the exposure t at a constant. Fixing the exposure and changing the mediator allows the elimination of other mechanisms and isolates the mediation effect via the mediator.

The ADE can be defined using the following:

$$\sum\{Y(t_1, M(t)) - Y(t_0, M(t))\}$$

Where ADE equals the difference in outcome due to a change in the exposure from not exposed (t_0) to exposed (t_1), holding the mediator at a constant that would be expected under exposure (M (t)). This represents the effect of exposure on the outcome that is not hypothesised to be transmitted via the mediator. The total effect equals the sum of the ACME and ADE.

Reference:

Imai K, Keele L, Tingley D, Yamamoto T. Unpacking the Black Box of Causality: Learning about Causal Mechanisms from Experimental and Observational Studies. *Am Polit Sci Rev* 2011; 105: 765–89.

Table S2. Linear regression models showing the association between duration of exposure to adversity (any, economic, psychosocial) across the lifecourse and cognitive function at 69 years.

Duration of exposure across the lifecourse to:	Cognitive state (ACE-III)			Processing speed			Verbal memory		
	β	95%CI	p-value	β	95%CI	p-value	β	95%CI	p-value
**Any adversity [linear trend]**									
Model 1[a]	**-0.39**	**-0.59; -0.20**	**<0.001**	-0.17	-0.37; 0.02	0.084	**-0.45**	**-0.63; -0.27**	**<0.001**
Model 2[b]	-0.06	-0.24; 0.12	0.510	-0.07	-0.27; 0.13	0.476	-0.14	-0.31; 0.03	0.102
**Economic adversity [linear trend]**									
Model 1[a]	**-0.51**	**-0.69; -0.32**	**<0.001**	**-0.23**	**-0.40; -0.06**	**0.010**	**-0.54**	**-0.70; -0.39**	**<0.001**
Model 2[b]	-0.12	-0.27; 0.04	0.147	-0.12	-0.30; 0.06	0.195	**-0.18**	**-0.32; -0.03**	**0.016**
**Psychosocial adversity [linear trend]**									
Model 1[a]	0.03	-0.23; 0.29	0.834	-0.13	-0.34; 0.09	0.255	**-0.23**	**-0.46; -0.01**	**0.044**
Model 2[b]	0.10	-0.13; 0.33	0.397	-0.09	-0.31; 0.12	0.402	-0.15	-0.36; 0.05	0.143

[a]Adjusted for sex

[b]Adjusted additionally for educational attainment and childhood (cognition and emotional problems) covariates.

Table S3. Linear regression models showing the association between duration of exposure to adversity (any, economic, psychosocial) across the lifecourse and mental health (Psychological distress), and between mental health (psychological distress) and cognitive function at 69 years.

	Cognitive state (ACE-III)			Processing speed			Verbal memory		
	β	95%CI	p-value	β	95%CI	p-value	β	95%CI	p-value
**Any adversity**									
Duration of exposure → Psychological distress	**0.46**	**0.26; 0.66**	**<0.001**	**0.46**	**0.28; 0.63**	**<0.001**	**0.46**	**0.28; 0.64**	**<0.001**
Psychological distress → Cognitive function	**-0.07**	**-0.11; -0.02**	**0.005**	**-0.05**	**-0.10; -0.004**	**0.035**	**-0.07**	**-0.11; -0.03**	**0.001**
**Economic adversity**									
Duration of exposure → Psychological distress	**0.42**	**0.21; 0.63**	**<0.001**	**0.44**	**0.26; 0.61**	**<0.001**	**0.45**	**0.27; 0.64**	**<0.001**
Psychological distress → Cognitive function	**-0.06**	**-0.11; -0.02**	**0.007**	**-0.05**	**-0.10; -0.003**	**0.038**	**-0.07**	**-0.11; -0.03**	**0.001**
**Psychosocial adversity**									
Duration of exposure → Psychological distress	**0.58**	**0.31; 0.84**	**<0.001**	**0.53**	**0.30; 0.76**	**<0.001**	**0.54**	**0.29; 0.80**	**<0.001**
Psychological distress → Cognitive function	**-0.07**	**-0.12; -0.02**	**0.004**	-0.05	-0.10; 0.00	0.051	**-0.07**	**-0.11; -0.03**	**0.001**

All models adjusted for socio-demographic (sex, educational attainment) and childhood (cognition and emotional problems) covariates.

Table S4. Causal mediation analysis showing the average direct, mediated (via mental health), and total effect of duration of exposure to adversity (any, economic, psychosocial) on cognitive function at 69 years.

	**Cognitive state (ACE-III)**		**Processing speed**		**Verbal memory**	
	β	95%CI	β	95%CI	β	95%CI
**Any adversity**						
ADE	-0.03	-0.20; 0.13	-0.10	-0.26; 0.08	-0.13	-0.28; 0.02
ACME	**-0.03**	**-0.06; -0.01**	-0.02	-0.04, 0.00	**-0.03**	**-0.06; -0.02**
Total effect	-0.07	-0.23; 0.09	-0.12	-0.28; 0.05	**-0.16**	**-0.31; -0.02**
**Economic**						
ADE	-0.05	-0.20; 0.09	-0.13	-0.29; 0.03	**-0.17**	**-0.31; -0.03**
ACME	**-0.03**	**-0.05; -0.01**	-0.02	-0.04; 0.00	**-0.03**	**-0.06; -0.02**
Total effect	-0.08	-0.23; 0.06	-0.14	-0.31; 0.01	**-0.20**	**-0.34; -0.07**
**Psychosocial**						
ADE	0.09	-0.14; 0.30	-0.10	-0.32; 0.12	-0.06	-0.26; 0.13
ACME	**-0.05**	**-0.08; -0.02**	-0.02	-0.04; 0.00	**-0.03**	**-0.06; -0.01**
Total effect	0.04	-0.18; 0.25	-0.11	-0.34; 0.10	-0.10	-0.30; 0.09

Adjusted for socio-demographic (sex, educational attainment) and childhood (cognition and emotional problems) covariates.
Abbreviations: ADE: average direct effect; ACME: average causal mediation effect.

Table S5. Linear regression models on the interaction between economic and psychosocial adversity on verbal memory at 69 years.

	**Verbal memory**		
	**β**	**95%CI**	**p-value**
**Interaction model**			
Economic x psychosocial adversity	-0.79	-3.73; 2.15	0.597

Liu Y, *et al. BMJ Open* 2023; 13:e074105. doi: 10.1136/bmjopen-2023-074105

Sensitivity analyses

Repeating steps from main analysis for a subset of participants with complete data on all three cognitive measures (N=1720)

Table S6. Linear regression models showing the association between duration of exposure to adversity (any, economic, psychosocial) across the lifecourse and cognitive function at 69 years (sensitivity analyses).

Duration of exposure across the lifecourse to:	Cognitive state (ACE-III)			Processing speed			Verbal memory		
	β	95%CI	p-value	β	95%CI	p-value	β	95%CI	p-value
**Any adversity [linear trend]**									
Model 1[a]	**-0.37**	**-0.55; -0.18**	**<0.001**	-0.17	-0.37; 0.04	0.107	**-0.46**	**-0.66; -0.27**	**<0.001**
Model 2[b]	-0.03	-0.19; 0.13	0.716	-0.06	-0.27; 0.15	0.579	-0.15	-0.33; 0.02	0.091

[a]Adjusted for sex

[b]Adjusted additionally for educational attainment and childhood (cognition and emotional problems) covariates.

Table S7. Causal mediation analysis showing the average direct, mediated (via mental health), and total effect of duration of exposure to adversity (any, economic, psychosocial) on cognitive function at 69 years.

	Cognitive state (ACE-III)		Processing speed		Verbal memory	
	β	95%CI	β	95%CI	β	95%CI
**Any adversity**						
ADE	0.01	-0.14; 0.16	0.01	-0.17; 0.20	-0.11	-0.28; 0.05
ACME	**-0.03**	**-0.06; -0.01**	-0.02	-0.05; 0.00	**-0.04**	**-0.06; -0.02**
Total effect	-0.02	-0.18; 0.13	-0.01	-0.20; 0.17	-0.15	-0.31; 0.01

Adjusted for socio-demographic (sex, educational attainment) and childhood (cognition and emotional problems) covariates. Abbreviations: ADE: average direct effect; ACME: average causal mediation effect.
