# Supplementary material for: Lifecourse investigation of the cumulative impact of adversity on cognitive function in old age and the mediating role of mental health: longitudinal birth cohort study
Source: BMJ Open. 2023 Nov 8;13(11):e074105. doi: 10.1136/bmjopen-2023-074105 (PMC10632868; doi:10.1136/bmjopen-2023-074105)
Supplement: Author's manuscript [file bmjopen-2023-074105.draft_revisions.pdf]

BMJ Open is committed to open peer review. As part of this commitment we make the peer review history of every article we publish publicly available.

When an article is published we post the peer reviewers' comments and the authors' responses online. We also post the versions of the paper that were used during peer review. These are the versions that the peer review comments apply to.

The versions of the paper that follow are the versions that were submitted during the peer review process. They are not the versions of record or the final published versions. They should not be cited or distributed as the published version of this manuscript.

BMJ Open is an open access journal and the full, final, typeset and author-corrected version of record of the manuscript is available on our site with no access controls, subscription charges or pay-per-view fees (http://bmjopen.bmj.com).

If you have any questions on BMJ Open's open peer review process please email info.bmjopen@bmj.com

A lifecourse investigation of the cumulative impact of adversity on cognitive function in old age and the mediating role of mental health: longitudinal birth cohort study

Journal:	*BMJ Open*
Manuscript ID	bmjopen-2023-074105
Article Type:	Original research
Date Submitted by the Author:	27-Mar-2023
Complete List of Authors:	Liu, Yiwen; University College London, MRC Unit for Lifelong Health and Ageing<br>Patalay, Praveetha; University College London, MRC Unit for Lifelong Health and Ageing; University College London, Centre for Longitudinal Studies, Social Research Institute<br>Stafford, Jean; University College London, MRC Unit for Lifelong Health and Ageing<br>Schott, Jonathan M.; University College London, Dementia Research Centre, UCL Queen Square Institute of Neurology<br>Richards, M; University College London, MRC Unit for Lifelong Health and Ageing
Keywords:	MENTAL HEALTH, Aging, EPIDEMIOLOGY, Dementia

SCHOLARONE™
Manuscripts

BMJ

I, the Submitting Author has the right to grant and does grant on behalf of all authors of the Work (as defined in the below author licence), an exclusive licence and/or a non-exclusive licence for contributions from authors who are: i) UK Crown employees; ii) where BMJ has agreed a CC-BY licence shall apply, and/or iii) in accordance with the terms applicable for US Federal Government officers or employees acting as part of their official duties; on a worldwide, perpetual, irrevocable, royalty-free basis to BMJ Publishing Group Ltd ("BMJ") its licensees and where the relevant Journal is co-owned by BMJ to the co-owners of the Journal, to publish the Work in this journal and any other BMJ products and to exploit all rights, as set out in our licence.

The Submitting Author accepts and understands that any supply made under these terms is made by BMJ to the Submitting Author unless you are acting as an employee on behalf of your employer or a postgraduate student of an affiliated institution which is paying any applicable article publishing charge ("APC") for Open Access articles. Where the Submitting Author wishes to make the Work available on an Open Access basis (and intends to pay the relevant APC), the terms of reuse of such Open Access shall be governed by a Creative Commons licence – details of these licences and which Creative Commons licence will apply to this Work are set out in our licence referred to above.

Other than as permitted in any relevant BMJ Author's Self Archiving Policies, I confirm this Work has not been accepted for publication elsewhere, is not being considered for publication elsewhere and does not duplicate material already published. I confirm all authors consent to publication of this Work and authorise the granting of this licence.

For peer review only

A lifecourse investigation of the cumulative impact of adversity on cognitive function in old age and the mediating role of mental health: longitudinal birth cohort study

Yiwen Liu[1], Praveetha Patalay[1,2], Jean Stafford[1], Jonathan M Schott[3], Marcus Richards[1]

Affiliations

[1]MRC Unit for Lifelong Health and Ageing at UCL, University College London, London, UK

[2]Centre for Longitudinal Studies, Social Research Institute, University College London, London, UK

[3]Dementia Research Centre, UCL Queen Square Institute of Neurology, London, UK

Corresponding author: Dr Yiwen Liu, MRC Unit for Lifelong Health & Ageing at UCL, University College London, London, UK, WC1E 7HB. Email: eva.liu@ucl.ac.uk

Word count: 3470

For peer review only

Abstract

Objective: To investigate the accumulating effect of adversities (any, economic, psychosocial) across the lifecourse (birth to 63 years) on cognitive function in older age, and the mediating role of mental health.

Design: National birth cohort study.

Setting: Great Britain.

Participants: 5362 singleton births within marriage in England, Wales and Scotland born within one week of March 1946, of which 2131 completed at least one cognitive assessment.

Main outcome measures: Cognitive assessments included the Addenbrooke's Cognitive Examination-III (ACE-III), as a measure of cognitive state, processing speed (timed-letter search task), and verbal memory (word learning task) at 69 years. Scores were standardised to the analytical sample. Mental health at 63 years was assessed using the 28-item General Health Questionnaire (GHQ-28), with scores standardised to the analytical sample.

Results: After adjusting for sex, increased duration of exposure to any adversity was associated with decreased performance on cognitive state (β = -0.27; 95%CI [-0.50, -0.03]) and verbal memory (β = -0.33; 95%CI [-0.54, -0.11]) at 69 years, although these effects were attenuated after adjusting for further covariates (childhood cognition and emotional problems, educational attainment). Analyses by type of adversity revealed stronger associations from economic adversity to verbal memory (β = -0.46; 95%CI [-0.61, -0.31]), with a small effect remaining even after adjusting for all covariates (β = -0.15; 95%CI [-0.28, -0.01]), and weaker associations from psychosocial adversity. Causal mediation analyses found that mental health mediated all associations between duration of exposure to adversity (any, economic, psychosocial) and cognitive function, with around 17% of the total effect of economic adversity on verbal memory attributable to mental health.

For peer review only - http://bmjopen.bmj.com/site/about/guidelines.xhtml

Conclusions: Improving mental health among older adults has the potential to reduce cognitive impairments, as well as mitigate against some of the effect of lifecourse accumulation of adversity on cognitive performance in older age.

Strengths and limitations of this study

- This is the first study to use mostly prospective indicators of adversity spanning six decades to assess the dose-response effect of adversities on cognitive function in late adulthood, as well as the mediating effect of mental health.
- The assessment of cognition and mental health in childhood allowed adjustment for important covariates to mitigate against reverse directionality.
- There may be bias from selective drop-out, with those who dropped out having lower childhood cognitive ability and educational attainment.
- The perceived impact of adversities and the clustering of different adversities were not considered in this study.

For peer review only

For peer review only - http://bmjopen.bmj.com/site/about/guidelines.xhtml

Introduction

Age-related disorders account for almost a quarter of the total burden of diseases worldwide, with cognitive and mental impairments in older age being one of the leading contributors.[1] There is some evidence that exposure to adversity is associated with reduced cognitive function in older age, particularly for economic adversity such as socio-economic circumstances (SEC).[2–6] However, few studies have adopted a lifecourse approach to examine prospectively measured adversities accumulated across multiple stages of development. According to the accumulation model – a key lifecourse model – persistent exposure to adversities can increase dysregulation of the hypothalamic-pituitary-adrenal (HPA)-axis, which over time could lead to physiological damage to neural and somatic systems.[7,8] It is therefore important to investigate whether persistent exposure to adversity across the lifecourse is associated with cognitive function in older adulthood to promote healthy cognitive ageing.

Experiencing an increased number of adversities (often assessed at one period in time) has been associated with poorer cognitive performance and greater decline in older adulthood.[2,4,9] Studies which examined the duration of exposure to the same adversity (e.g., SEC, maltreatment) also found an association with cognition in mid- and older adulthood,[5,10,11] although only one study used prospectively collected measures of adversity.[11] These findings suggest that experiencing multiple adversities at the same time or experiencing the same adversity persistently across time may both be associated with cognitive impairments in later life. Further research is needed on whether increased duration of exposure to any adversity (regardless of the type) across the lifecourse (from childhood to older adulthood) also shows a dose-response association with cognitive function in older age.

The accumulating effect of adversity – as well as having direct effects on cognitive function – may also have indirect effects via mental health. There is consistent evidence that persistent

For peer review only - http://bmjopen.bmj.com/site/about/guidelines.xhtml

exposure to adversities at the same time and across time is associated with increased psychological distress in older adulthood.[12–14] Mental health was also recently identified as one of the leading modifiable risk factors for dementia;[15] both mental health and cognitive function are intertwined across the lifecourse,[16,17] and there is some evidence that mental health in mid-adulthood predicted cognition in older adulthood, but not vice versa.[16] It is therefore plausible that mental health may be a key mechanism that mediates the association between the duration of exposure to adversity across the lifecourse and cognitive function in older age. There is some research supporting the role of depression as a mediator between one type of adversity (i.e. maltreatment, financial hardships) and cognitive function, although studies have mainly relied on retrospective reports or were limited to one stage of the lifecourse.[10,18] Therefore, further research on the mediating role of mental health is needed using prospective measures of different types of adversity across multiple stages of the lifecourse.

The aim of this study was to examine the longitudinal association between lifecourse exposure to adversity and cognitive function in older age, and the mediating role of mental health, using data from the MRC National Survey of Health and Development (NSHD; the British 1946 birth cohort). The use of this cohort is particularly advantageous given the long follow-up period, the use of repeated measures across multiple stages of the lifecourse, and the assessment of cognition and mental health in childhood. The latter is especially important for control of potential reverse directionality (i.e. early cognitive development and mental health being associated with exposure to adversities across the lifecourse).[19,20] We proposed two primary research questions: first, we examined the accumulation of adversity across the lifecourse (duration of exposure to any adversity) with cognitive function at 69 years; second, we examined to what extent mental health at 63 years mediated this relationship. As secondary analyses, we examined economic and psychosocial adversity – two frequently assessed areas of adversity in the literature[21,22] – separately to test for possible specificity.

For peer review only - http://bmjopen.bmj.com/site/about/guidelines.xhtml

Methods

Sample

The NSHD originally consisted of 5,362 male and female singleton births within marriage in England, Wales and Scotland, born within one week of March 1946 (http://www.nshd.mrc.ac.uk/nshd). At age 69 a target sample of 2,698 study members who were still alive and had a known current address in mainland Britain were invited to take part in a home visit.[23] Characteristics of those who were not assessed have been reported previously.[23] Ethical approval has been obtained from the Greater Manchester Local Research Ethics Committee and the Scotland Research Ethics Committee, and participants provided written informed consent at each data collection. The analytical sample included in this study are those who were still alive at 69 years and completed any cognitive assessment (Fig. 1).

For peer review

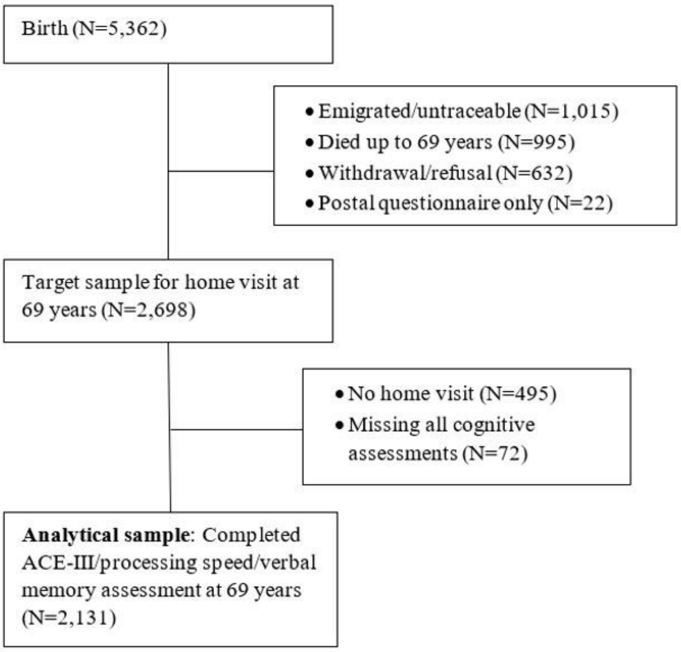

Fig. 1. Sample flow diagram. *ACE-III: Addenbrooke's Cognitive Examination-III*

For peer review only - http://bmjopen.bmj.com/site/about/guidelines.xhtml

Patient and Public Involvement

Patients or the public were not involved in the design, analysis or interpretation of this study.

Measures

Indicators of adversity

Indicators of adversity were broadly grouped into economic and psychosocial domains across four stages of the lifecourse (see supplementary materials: Table S1). Each was coded as binary (presence or absence).

Childhood (<16 years)

Economic: (i) overcrowding (≥2 people per room); (ii) lack of essential household amenities (no sole use of kitchen or bathroom, or no running hot water); (iii) below average housing condition rated by the interviewer; and (iv) father's occupational status (unskilled or unemployed, reported at age 11 or 15 years).

Psychosocial: (i) separation from mother for ≥28 days (not due to illness or hospitalisation); (ii) affectionless control parenting, indicated by low maternal care and high overprotectiveness, retrospectively reported at age 43 using the Parent Bonding Instrument;[24] (iii) maltreatment (retrospectively self-reported at age 43); (iv) parental divorce; or (v) difficulties with peers (not able to make friends easily or not being popular with other children, rated by the teacher when participants were 13-15 years).

Young adulthood (20-36 years)

Economic: (i) overcrowding; (ii) lack of essential household amenities; (iii) financial hardships (going without necessities due to a shortage of money; difficulty managing on current income); (iv) unemployment; or (v) any work-related crisis.

For peer review only - http://bmjopen.bmj.com/site/about/guidelines.xhtml

Psychosocial: (i) social isolation (never or rarely see friends or family); and (ii) divorce or separation from partner.

Mid-adulthood (43-53 years)

Economic: (i) any work-related stress (lost or feared losing employment, or experienced crisis at work during the last 12 months); (ii) financial hardships (same items as young adulthood, with an additional item on unable to pay bills); or (iii) inadequate living conditions (no central heating or running hot water, or damp in the property).

Psychosocial: (i) social isolation (never see friends or family; never have people to visit); (ii) lack of social support (no family or friends to talk to; have no one to help in times of crisis); (iii) lost contact with relatives or friends; (iv) difficulties with own children; or (v) divorce or separation from partner.

Late adulthood (60-64 years)

The same indicators from mid-adulthood were used, apart from "inadequate living conditions" in the economic domain, which was not assessed at 60-64 years.

Mental health: Psychological distress (63 years)

Mental health at 63 years was assessed using the 28-item General Health Questionnaire (GHQ-28), a self-administered questionnaire measuring psychological distress across four areas (somatic symptoms, anxiety and insomnia, social dysfunction and severe depression).[25] Each item was rated on a 4-point Likert scale from 0 (not at all) to 3 (much more than usual). A summary score was calculated which ranged from 0 to 84 (higher distress), and scores were standardised to the population.

For peer review only - http://bmjopen.bmj.com/site/about/guidelines.xhtml

Cognitive function (69 years)

Cognitive state

The Addenbrooke's Cognitive Examination-III (ACE-III) was originally developed to screen for mild dementia and cognitive impairments in a clinical setting but is also an appropriate measure of general cognitive state.[26–28] It covers 5 domains of cognitive abilities including attention, memory, language, verbal fluency and visuospatial skills, and was conducted via the ACEmobile app on iPad, with help from the interviewer.[27,28] Scores are calculated for each cognitive domain and an overall score (max=100) was derived from the sum of these domains, with higher scores indicating higher cognitive function.

Processing speed

Processing speed was assessed using a timed-letter search task. Participants were instructed to cross out target letters "P" and "W" embedded among non-target letters as quickly and accurately as possible within 1 minute. A total score representing the speed of letters identified (position reached at the end of the trial) was the main outcome, with a maximum score of 600.

Verbal memory

Verbal memory was assessed using a word learning task. Participants were shown a list of 15 words over 3 rounds and were asked to write down as many as they could remember at the end of each. A total score indicating the number of words correctly recalled across all rounds was calculated, with a maximum score of 45.

Covariates

Two sets of covariates were included: socio-demographic factors, including sex at birth (male as the reference group), and highest educational attainment by 26 years (categorised into

For peer review only - http://bmjopen.bmj.com/site/about/guidelines.xhtml

ordinary ‘O’ level or below vs advanced ‘A’ level or above, with the latter as the reference group); and childhood measures, including cognitive abilities at 8 years (derived from 4 tests from the National Foundation for Educational Research,[29] standardised to the whole population), and emotional problems at 13-15 years, rated by the teacher and grouped into none (reference group), mild or severe.

Statistical analyses

All statistical analyses were performed in R (version 3.6.2). Scores on each cognitive outcome were standardised to the analytical sample.

Accumulation model

We derived a variable indicating the presence or absence of any adversity at each stage of the lifecourse (childhood, young, mid and late adulthood). These were summed to calculate the duration of exposure to any adversity and ranged from 0 (no exposure) to 4 (exposure at all four times). Using this measure, we tested for a linear dose-response association with cognitive function (cognitive state, processing speed, verbal memory) at 69 years. This was first adjusted for sex, then fully adjusted for all covariates.

Mediation model

Linear regression models first examined the association between the duration of exposure to any adversity and psychological distress at 63 years, and between psychological distress at 63 years and cognitive functioning at 69 years, adjusted for all covariates. Causal mediation analysis then estimated the indirect effect of duration of exposure to any adversity on cognitive function via psychological distress at 63 years. The R package “mediation” was used, which performs causal mediation analysis within a counterfactual framework.[30–32] The total effect of

For peer review only - http://bmjopen.bmj.com/site/about/guidelines.xhtml

For peer review only

lifecourse adversity on cognitive function was estimated, which is composed of an average direct effect (ADE), and an average causal mediation effect (ACME) (See supplementary materials for detailed description on the ADE and ACME).[30–33]

Specificity of adversity

We repeated the accumulation and mediation models for economic and psychosocial domains separately to test for possible specificity.

Additional exploratory analysis

We further explored the interaction (tested at $p<0.10$) between economic and psychosocial adversity if both showed associations with cognitive function.

Missing data

Missing data was handled using multivariable imputation by chained equations (MICE) in R[34] with 20 imputed datasets. Imputation was performed on individual indicators of adversity at each stage of the lifecourse, which were included as auxiliary variables along with all variables included in the analysis. Derived adversity variables were calculated post-imputation. Imputation was performed up to the maximum analytical sample size for each cognitive outcome, and estimates were combined and pooled together using Rubin's rule.[34]

Results

Sample characteristics

In total, 2,131 participants completed at least one cognitive assessment at 69 years (cognitive state: *N*=1,762; processing speed: *N*=2,111; verbal memory: *N*=2,074). The distribution of adversity variables in the sample can be found in table 1. Economic adversity was more

For peer review only - http://bmjopen.bmj.com/site/about/guidelines.xhtml

prevalent than psychosocial adversity in childhood, young and mid-adulthood, with roughly half of participants reporting it at each stage. The rates of both adversities were reduced in older adulthood. Across the lifecourse, 2.8% of participants were not exposed to any adversity and 16% experienced it chronically (4 times).

Table 1. Sample characteristics.

	**N=2,131**
**Childhood (<17 years)**	
Economic adversity	1353 (63.6%)
Psychosocial adversity	846 (39.7%)
**Young adulthood (20-36 years)**	
Economic adversity	993 (48.6%)
Psychosocial adversity	648 (31.4%)
**Mid-adulthood (43-53 years)**	
Economic adversity	1119 (53.4%)
Psychosocial adversity	1105 (52.7%)
**Late adulthood (60-64 years)**	
Economic adversity	377 (20.0%)
Psychosocial adversity	511 (25.8%)
**Duration of exposure across the lifecourse to:**	
**Any adversity**	
0 (None)	60 (2.8%)
1 (Once)	318 (14.9%)
2 (Twice)	676 (31.7%)
3 (Three times)	737 (34.6%)
4 (Four times)	340 (16.0%)
**Economic**	
0 (None)	210 (9.9%)
1 (Once)	636 (29.8%)
2 (Twice)	754 (35.4%)
3 (Three times)	426 (20.0%)
4 (Four times)	105 (4.9%)
**Psychosocial**	
0 (None)	437 (20.5%)
1 (Once)	712 (33.4%)
2 (Twice)	614 (28.8%)
3 (Three times)	302 (14.2%)
4 (Four times)	66 (3.1%)
**Covariates**	
Sex (Female)	1086 (51.0%)
Educational attainment (26 years; O-levels or below)	1197 (59.3%)
Childhood internalising problems (13-15 years)	
0 (Absent)	1001 (52.4%)
1 (Mild)	713 (37.3%)
2 (Severe)	196 (10.3%)
**Mental health**	
Psychological distress (GHQ-28 total score; max = 84) (M; SD)	16.38 (8.02)
**Cognitive functioning**	
Cognitive state (ACE-III total score; max = 100) (M; SD)	91.52 (6.01)
Processing speed (Max = 600) (M; SD)	262.30 (74.14)
Verbal memory (Max = 45) (M; SD)	22.17 (6.07)

For peer review only - http://bmjopen.bmj.com/site/about/guidelines.xhtml

For peer review only

Accumulation model

Increased duration of exposure to any adversity across the lifecourse was associated with lower performance on cognitive state (ACE-III) and verbal memory, but not processing speed, at 69 years. Each additional period of exposure was associated with a 0.3 standard deviation (SD) decrease in cognitive state (β = -0.27; 95%CI [-0.50, -0.03]) and verbal memory (β = -0.33; 95%CI [-0.54, -0.11]) (Fig. 2, supplementary materials: Table S2). However, these effect sizes were attenuated in fully adjusted models.

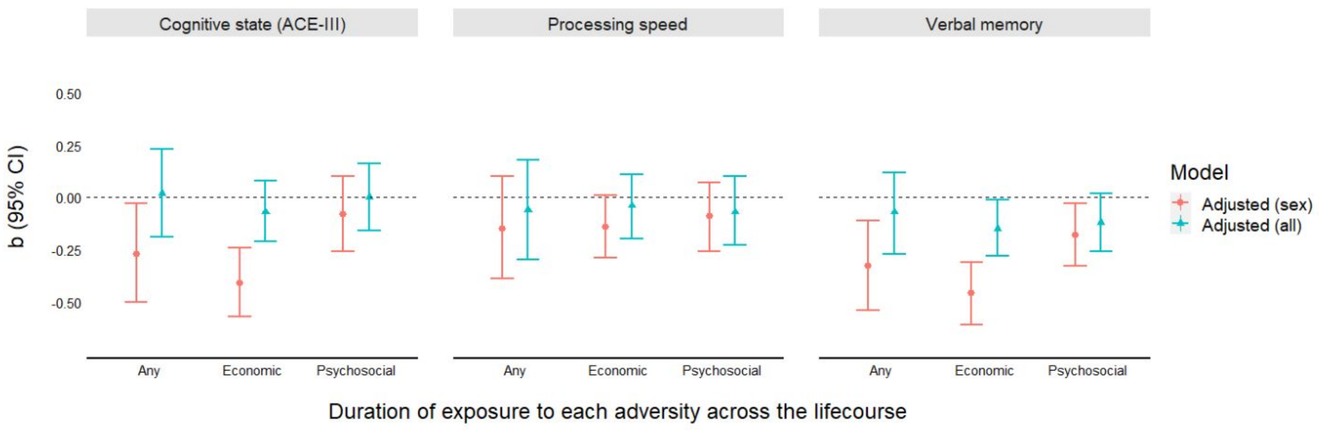

Fig. 2. Standardised estimates on the association between accumulating duration of exposure to adversity and each cognitive function at 69 years.

Mediation model

Each increased period of exposure to any adversity across the lifecourse was associated with around half a SD increase in psychological distress, and each SD increase in psychological distress was associated with a 0.07 SD decrease in cognitive state and verbal memory, and a 0.05 SD decrease in processing speed (Fig. 3; supplementary materials: Table S3). Causal mediation analysis found no direct effect of duration of exposure to any adversity on cognitive

For peer review only - http://bmjopen.bmj.com/site/about/guidelines.xhtml

functioning; however, mediated pathways were found to all three cognitive measures via increased psychological distress at 63 years (Table 2).

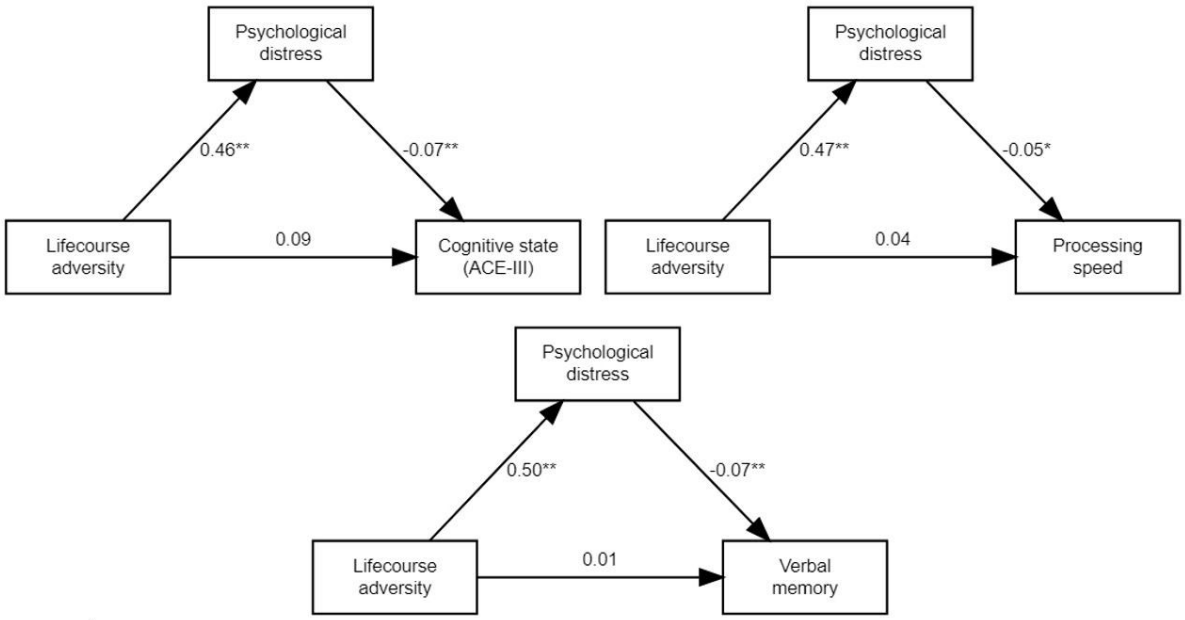

Fig. 3. Mediation models showing the direct and indirect effects of lifecourse adversity (duration of exposure to any adversity) on each cognitive function via mental health (psychological distress). **p<0.05; ** p<0.01*

review only

Specificity of adversity

Economic adversity

Each additional period of exposure to economic adversity was associated with a 0.4 (95%CI [-0.58, -0.24]) and 0.5 (95%CI [-0.61, -0.31]) SD decrease in cognitive state and verbal memory, respectively (Fig. 2; supplementary materials, Table S4). Both were attenuated in fully adjusted models, although a small effect remained on verbal memory (β = -0.15; 95%CI [-0.28, -0.01]) (Fig. 2; supplementary materials: Table S2). No association was found for processing speed.

For peer review only - http://bmjopen.bmj.com/site/about/guidelines.xhtml

Mediated effects were found to all three cognitive measures via increased psychological distress at 63 years (Table 2; supplementary materials: Table S3, Fig. S1). Increased exposure to economic adversity showed both a direct and indirect effect on verbal memory, with around 17% of its total effect mediated via psychological distress (calculated as the ratio between total effect and mediated effect).

Psychosocial adversity

A small dose-response effect was found between duration of exposure to psychosocial adversity and verbal memory (β = -0.18; 95%CI [-0.33, -0.03]), although this became attenuated when adjusted for all covariates (Fig. 2; supplementary materials: Table S2). No other associations were found for cognitive state or processing speed. Causal mediation analysis showed no direct effect of psychosocial adversity, although mediated effects were found via psychological distress to both cognitive state and verbal memory, with weaker evidence for mediation on processing speed (Table 2; supplementary materials Table S3, Fig. S1).

Table 2. Causal mediation analysis showing the average direct, mediated (via mental health), and total effect of duration of exposure to adversity (any, economic, psychosocial) on cognitive function at 69 years.

	**Cognitive state (ACE-III)**		**Processing speed**		**Verbal memory**	
	β	95%CI	β	95%CI	β	95%CI
**Any adversity**						
ADE	0.09	-0.10; 0.29	0.04	-0.15; 0.23	0.01	-0.17; 0.18
ACME	**-0.04**	**-0.07; -0.01**	**-0.03**	**-0.05, -0.01**	**-0.04**	**-0.06; -0.01**
Total effect	0.06	-0.13; 0.24	0.01	-0.17; 0.20	-0.03	-0.20; 0.14
**Economic**						
ADE	-0.03	-0.17; 0.11	-0.01	-0.14; 0.15	**-0.15**	**-0.28; -0.02**
ACME	**-0.03**	**-0.05; -0.01**	**-0.03**	**-0.05; -0.01**	**-0.03**	**-0.05; -0.01**
Total effect	-0.06	-0.20; 0.08	-0.02	-0.17; 0.12	**-0.18**	**-0.31; -0.05**
**Psychosocial**						
ADE	0.08	-0.06; 0.22	-0.01	-0.16; 0.14	-0.08	-0.21; 0.05
ACME	**-0.03**	**-0.06; -0.01**	-0.02	-0.05; 0.00	**-0.03**	**-0.05; -0.01**
Total effect	0.05	-0.10; 0.19	-0.03	-0.18; 0.11	-0.11	-0.25; 0.02

Adjusted for socio-demographic (sex, educational attainment) and childhood (cognition and emotional problems) covariates.
Abbreviations: ADE: average direct effect; ACME: average causal mediation effect.

For peer review only - http://bmjopen.bmj.com/site/about/guidelines.xhtml

Additional exploratory analysis

Given that both economic and psychosocial adversity were associated separately with verbal memory, an interaction term between them was included in the model to examine whether there was evidence for a multiplicative effect, although no interaction was detected ($p = 0.855$) (supplementary materials, Table S4).

Discussion

The overall aim of this paper was to use a lifecourse approach to examine the accumulation of adversity from childhood to adulthood on cognitive function in older adults and estimate the mediating role of mental health on the causal pathway between them. Some support for the accumulating effect of adversity was found on cognitive state and verbal memory, but not processing speed. Although most of these effects were attenuated after adjusting for covariates, mediated effects via psychological distress were identified for all three cognitive measures. Stronger associations were found from economic compared to psychosocial adversity, although similar mediated effects via psychological distress were found regardless of the type of adversity or cognitive measure.

The accumulating effect of adversity on cognitive function expands on previous research[2,4,6,11] by using comprehensive and primarily prospective assessments of two domains of adversity across four lifecourse stages, and found dose-response associations with both cognitive state and verbal memory. However, most of these associations were attenuated after adjusting for educational attainment and childhood characteristics (cognition and emotional problems),[3,35–39] highlighting the importance of having information on these outcomes from earlier in the lifecourse when trying to estimate effects in older adults. Childhood emotional problems are also a predictor of mental health in later life,[40,41] which has been associated with cognition

For peer review only - http://bmjopen.bmj.com/site/about/guidelines.xhtml

prospectively,[16,17] and may also increase risk of being exposed to further adversities later in life.[42,43] Therefore, although the accumulating duration of exposure to adversity may have a small dose-response effect on cognitive function in older age, most of this can be accounted for by socio-demographic and cognitive and emotional function in childhood.

Although there was weaker evidence for direct effects, mediated effects were found via psychological distress to all three cognitive measures. A few studies have previously found an indirect effect of specific types of adversity (e.g., maltreatment, financial hardships) on cognition in adulthood via depressive symptoms.[10,18] However, this is the first study to our knowledge to show prospectively that increased exposure to any adversity across the lifecourse was indirectly associated with both cognitive state and specific domains of cognitive function in older adulthood via mental health in older age. One proposed mechanism is that increased psychological distress may lead to over-activation of the HPA-axis and increased cortisol production, which have been associated with cognitive impairments.[44,45] Mental health in older adulthood may therefore be considered as both an antecedent for cognitive impairments later in life as well as a consequence of adversities accumulated across the lifecourse. Given that late-life depression was recently identified as one of the leading modifiable risk factors for dementia,[15] improving mental health in older adults may therefore not only directly improve cognitive function in older age, but could also mitigate some of the accumulating effect of lifecourse adversities on cognition.

Different findings also emerged when economic and psychosocial adversities were examined, with stronger associations from economic adversity to verbal memory compared to psychosocial adversity. There is some research suggesting that deprivation (e.g., economic) related adversities may impact more on cognitive processes,[46] possibly via reducing cognitive bandwidth and increasing cognitive load,[47,48] as well as increasing brain atrophy through stress

For peer review only - http://bmjopen.bmj.com/site/about/guidelines.xhtml

accumulation.[49,50] On the other hand, psychosocial adversities may affect emotional reactivity more than cognitive functioning.[46] Although similar mediated effects via psychological distress were found from both types of adversity, this only accounted for a part of the total effect of economic adversity on verbal memory, whereas the effect of psychosocial adversity on verbal memory was wholly mediated, suggesting that mental health may be the primary pathway from psychosocial adversity to cognitive function. Other potential mechanisms such as cardiometabolic health may also mediate the association between economic adversity and cognitive function and requires further investigation.[51] Nevertheless, an important possibility is that improving mental health in older adults could help improve cognitive function in the ageing population and mitigate some of the risks associated with chronic adversities. Given the social and health costs associated with cognitive impairments and dementia[52] – combined with the cost of mental ill-health in the population[53] – increasing investment in mental health services may be crucial, in the long term, to promote healthy cognitive and mental ageing.

Strengths and limitations

The longitudinal nature of this study offers several strengths. This is the first study to our knowledge to use mostly prospective indicators of adversity across different lifecourse stages to show the accumulating effect of duration of exposure to adversity on cognitive functioning in late adulthood. The assessment of cognition and mental health in childhood also allowed adjustment for important covariates and helped to mitigate against reverse directionality, although it does not fully exclude this possibility. The inclusion of two different domains of adversity (economic, psychosocial), as well as three different cognitive measures, further allowed testing of specificity in the associations.

There are also some limitations that should be considered. For instance, there may be bias from selective drop-out, as previous research has suggested that those who dropped out were more

For peer review only - http://bmjopen.bmj.com/site/about/guidelines.xhtml

likely to have lower childhood cognitive ability and educational attainment.[23] This may have led to an underestimation in the association between exposure to adversity and cognitive functioning. In addition, certain adversities may not be experienced in the same way across different lifecourse stages. There is some evidence suggesting that the perceived impact of adversity should also be considered in the accumulation model, as adverse events that are perceived as life changing may show stronger accumulating effect.[54,55] Therefore, caution is needed in assuming that the same adverse experience is being captured across the lifecourse. The lack of ethnic diversity in the cohort also limits the generalisability of these findings, particularly as people from ethnic minority backgrounds are more likely to experience adversities[56] and may be disproportionally affected by them. Furthermore, certain adversities may also co-occur as risk clusters or risk chains.[57–59] Future research should therefore include a risk clustering component to the accumulation model when examining the impact of lifecourse adversity on cognitive ageing.

Conclusion

Overall, findings from this study suggest that increased duration of exposure to any adversity – particularly economic adversity – has a small dose-response effect on cognitive state and verbal memory in older age. Mental health mediated the accumulating effect of all types of adversity (any, economic, psychosocial) on cognition, suggesting that routinely assessing and improving mental health in older adults may also help improve cognitive function in older age. Future studies should also account for the co-occurrence of adversities simultaneously and across time, as well as investigate other potential mediators such as cardiometabolic health, to better understand pathways to cognitive ageing.

For peer review only - http://bmjopen.bmj.com/site/about/guidelines.xhtml

Funding: This study is funded by the UK Medical Research Council which provides core funding for the NSHD (MC_UU_00019/1). MR additionally reports funding from MRC (MC_UU_00019/3). JMS acknowledges the support of the National Institute for Health Research University College London Hospitals Biomedical Research Centre, Alzheimer's Research UK, Medical Research Council, UK Dementia Research Institute and Alzheimer's Association. The funders had no involvement in study design, data collection, analysis, interpretation or the decision to submit for publication. The views expressed are those of the author(s) and YL serves as guarantor for the contents of this paper.

Declaration of interest: None

Acknowledgements

We are grateful to all study members who took part in the NSHD and to the study team members who helped to collect the data over the last seven decades.

Author contributions

Conceptualisation: YL, MR, PP

Methodology: YL, PP, JS

Funding acquisition: MR, JMS, PP

Formal analysis: YL

Writing – Original draft: YL

Writing – Review and editing: All authors

Data availability

The data that support the findings of this study are available to bona fide researchers upon request to the NSHD Data Sharing Committee. For more information see: http://www.nshd.mrc.ac.uk/data.

For peer review only

References

1 Prince MJ, Wu F, Guo Y, *et al.* The burden of disease in older people and implications for health policy and practice. *The Lancet* 2015; **385**: 549–62.

2 Chen R, Williams DR, Nishimi K, Slopen N, Kubzansky LD, Weuve J. A life course approach to understanding stress exposures and cognitive function among middle-aged and older adults. *Soc Sci Med* 2022; **314**: 115448.

3 Greenfield EA, Moorman SM. Childhood Socioeconomic Status and Later Life Cognition: Evidence From the Wisconsin Longitudinal Study. *J Aging Health* 2019; **31**: 1589–615.

4 Lee Y, Back JH, Kim J, Byeon H. Multiple Socioeconomic Risks and Cognitive Impairment in Older Adults. *Dement Geriatr Cogn Disord* 2010; **29**: 523–9.

5 Lyu J, Burr JA. Socioeconomic Status Across the Life Course and Cognitive Function Among Older Adults: An Examination of the Latency, Pathways, and Accumulation Hypotheses. *J Aging Health* 2016; **28**: 40–67.

6 Shrira A. Greater age-related decline in markers of physical, mental and cognitive health among Israeli older adults exposed to lifetime cumulative adversity. *Aging Ment Health* 2014; **18**: 610–8.

7 McEwen BS, Rasgon NL. The Brain and Body on Stress: Allostatic Load and Mechanisms for Depression and Dementia. Oxford University Press, 2018 https://oxfordmedicine.com/view/10.1093/med/9780190603342.001.0001/med-9780190603342-chapter-2 (accessed Feb 15, 2022).

8 Power C, Kuh D, Morton S. From Developmental Origins of Adult Disease to Life Course Research on Adult Disease and Aging: Insights from Birth Cohort Studies. *Annu Rev Public Health* 2013; **34**: 7–28.

9 Shrira A. Greater age-related decline in markers of physical, mental and cognitive health among Israeli older adults exposed to lifetime cumulative adversity. *Aging Ment Health* 2014; **18**: 610–8.

10 Künzi M, Gheorghe DA, Kliegel M, Ballhausen N, Gallacher J, Bauermeister S. Cumulative life course adversity, mental health, and cognition in the UK biobank. *Sci Rep* 2022; **12**: 14700.

11 Zeki Al Hazzouri A, Elfassy T, Sidney S, Jacobs D, Pérez Stable EJ, Yaffe K. Sustained Economic Hardship and Cognitive Function: The Coronary Artery Risk Development in Young Adults Study. *Am J Prev Med* 2017; **52**: 1–9.

12 Ejlskov L, Bøggild H, Kuh D, Stafford M. Social relationship adversities throughout the lifecourse and risk of loneliness in later life. *Ageing Soc* 2020; **40**: 1718–34.

13 Hatch SL, Mishra G, Hotopf M, Jones PB, Kuh D. Appraisals of stressors and common mental disorder from early to mid-adulthood in the 1946 British birth cohort. *J Affect Disord* 2009; **119**: 66–75.

14 Shrira A. The effect of lifetime cumulative adversity on change and chronicity in depressive symptoms and quality of life in older adults. *Int Psychogeriatr* 2012; **24**: 1988–97.

15 Livingston G, Huntley J, Sommerlad A, *et al.* Dementia prevention, intervention, and care: 2020 report of the Lancet Commission. *The Lancet* 2020; **396**: 413–46.

For peer review only - http://bmjopen.bmj.com/site/about/guidelines.xhtml

16 John A, Rusted J, Richards M, Gaysina D. Bidirectional relation between affective symptoms and cognitive function from middle to late adulthood: a population-based birth cohort study. *Aging Ment Health* 2021; **25**: 1642–8.

17 Richards M, Hatch SL. A life course approach to the development of mental skills. *J Gerontol B Psychol Sci Soc Sci* 2011; **66 Suppl 1**: i26-35.

18 Cadar D, Kobayashi LK, Steptoe AP, Demakakos P. OP24 The mediating pathways of the association between adverse childhood experiences and cognitive health in later life. *J Epidemiol Community Health* 2020; **74**: A11–2.

19 Verlinden M, Veenstra R, Ghassabian A, *et al.* Executive Functioning and Non-Verbal Intelligence as Predictors of Bullying in Early Elementary School. *J Abnorm Child Psychol* 2014; **42**: 953–66.

20 Zych I, Farrington DP, Llorent VJ, Ribeaud D, Eisner MP. Childhood Risk and Protective Factors as Predictors of Adolescent Bullying Roles. *Int J Bullying Prev* 2021; **3**: 138–46.

21 Lawn RB, Anderson EL, Suderman M, *et al.* Psychosocial adversity and socioeconomic position during childhood and epigenetic age: analysis of two prospective cohort studies. *Hum Mol Genet* 2018; **27**: 1301–8.

22 Sousa ACP de A, Guerra RO, Tu MT, Phillips SP, Guralnik JM, Zunzunegui M-V. Lifecourse Adversity and Physical Performance across Countries among Men and Women Aged 65-74. *PLOS ONE* 2014; **9**: e102299.

23 Kuh D, Wong A, Shah I, *et al.* The MRC National Survey of Health and Development reaches age 70: maintaining participation at older ages in a birth cohort study. *Eur J Epidemiol* 2016; **31**: 1135–47.

24 Parker G, Tupling H, Brown LB. A parental bonding instrument. *Br J Med Psychol* 1979; **52**: 1–10.

25 Goldberg DP, Hillier VF. A scaled version of the General Health Questionnaire. *Psychol Med* 1979; **9**: 139–45.

26 Noone P. Addenbrooke's Cognitive Examination-III. *Occup Med* 2015; **65**: 418–20.

27 James S-N, Davis D, O'Hare C, *et al.* Lifetime affective problems and later-life cognitive state: Over 50 years of follow-up in a British birth cohort study. *J Affect Disord* 2018; **241**: 348–55.

28 Richards M, James S-N, Sizer A, *et al.* Identifying the lifetime cognitive and socioeconomic antecedents of cognitive state: seven decades of follow-up in a British birth cohort study. *BMJ Open* 2019; **9**: e024404.

29 Pigeon DA. Tests used in the 1954 and 1957 surveys. *Home Sch* 1964; : 129–32.

30 Imai K, Keele L, Tingley D, Yamamoto T. Unpacking the Black Box of Causality: Learning about Causal Mechanisms from Experimental and Observational Studies. *Am Polit Sci Rev* 2011; **105**: 765–89.

31 Imai K, Keele L, Tingley D. A general approach to causal mediation analysis. *Psychol Methods* 2010; **15**: 309–34.

32 Imai K, Keele L, Yamamoto T. Identification, Inference and Sensitivity Analysis for Causal Mediation Effects. *Stat Sci* 2010; **25**: 51–71.

For peer review only - http://bmjopen.bmj.com/site/about/guidelines.xhtml

33 Tingley D, Yamamoto T, Hirose K, Keele LJ, Imai K. mediation: R Package for Causal Mediation Analysis. *J Stat Softw* 2014; **59**: 1–38.

34 Buuren SV, Groothuis-oudshoorn K. MICE: Multivariate imputation by chained equations in R. *J Stat Softw* 2011; **45**: 1–67.

35 Kuiper JS, Smidt N, Zuidema SU, Comijs HC, Oude Voshaar RC, Zuidersma M. A longitudinal study of the impact of social network size and loneliness on cognitive performance in depressed older adults. *Aging Ment Health* 2020; **24**: 889–97.

36 Richards M, Wadsworth MEJ. Long term effects of early adversity on cognitive function. *Arch Dis Child* 2004; **89**: 922–7.

37 Staff RT, Chapko D, Hogan MJ, Whalley LJ. Life course socioeconomic status and the decline in information processing speed in late life. *Soc Sci Med* 2016; **151**: 130–8.

38 Ding R, He P. Associations between childhood adversities and late-life cognitive function: Potential mechanisms. *Soc Sci Med* 2021; **291**: 114478.

39 Halpin AB, MacAulay RK, Boeve AR, D'Errico LM, Michaud S. Are Adverse Childhood Experiences Associated with Worse Cognitive Function in Older Adults? *J Int Neuropsychol Soc* 2021; : 1–10.

40 Colman I, Wadsworth MEJ, Croudace TJ, Jones PB. Forty-Year Psychiatric Outcomes Following Assessment for Internalizing Disorder in Adolescence. *Am J Psychiatry* 2007; **164**: 126–33.

41 Copeland WE, Alaie I, Jonsson U, Shanahan L. Associations of Childhood and Adolescent Depression With Adult Psychiatric and Functional Outcomes. *J Am Acad Child Adolesc Psychiatry* 2021; **60**: 604–11.

42 Groot S de, Veldman K, Iii BCA, Bültmann U. Work functioning among young adults: the role of mental health problems from childhood to young adulthood. *Occup Environ Med* 2022; **79**: 217–23.

43 Stansfeld SA, Clark C, Caldwell T, Rodgers B, Power C. Psychosocial work characteristics and anxiety and depressive disorders in midlife: the effects of prior psychological distress. *Occup Environ Med* 2008; **65**: 634–42.

44 Keller J, Gomez R, Williams G, *et al.* HPA axis in major depression: cortisol, clinical symptomatology and genetic variation predict cognition. *Mol Psychiatry* 2017; **22**: 527–36.

45 Wingenfeld K, Wolf OT. HPA Axis Alterations in Mental Disorders: Impact on Memory and its Relevance for Therapeutic Interventions. *CNS Neurosci Ther* 2011; **17**: 714–22.

46 Sheridan MA, Shi F, Miller AB, Salhi C, McLaughlin KA. Network structure reveals clusters of associations between childhood adversities and development outcomes. *Dev Sci* 2020; **23**: e12934.

47 Mani A, Mullainathan S, Shafir E, Zhao J. Poverty Impedes Cognitive Function. *Science* 2013; **341**: 976–80.

48 Shah AK, Mullainathan S, Shafir E. Some Consequences of Having Too Little. *Science* 2012; **338**: 682–5.

For peer review only

For peer review only - http://bmjopen.bmj.com/site/about/guidelines.xhtml

49 Butterworth P, Cherbuin N, Sachdev P, Anstey KJ. The association between financial hardship and amygdala and hippocampal volumes: results from the PATH through life project. *Soc Cogn Affect Neurosci* 2012; **7**: 548–56.

50 Elbejjani M, Fuhrer R, Abrahamowicz M, *et al.* Life-Course Socioeconomic Position and Hippocampal Atrophy in a Prospective Cohort of Older Adults. *Psychosom Med* 2017; **79**: 14–23.

51 Oi K, Haas S. Cardiometabolic Risk and Cognitive Decline: The Role of Socioeconomic Status in Childhood and Adulthood. *J Health Soc Behav* 2019; **60**: 326–43.

52 Prince MJ, Wimo A, Guerchet MM, Ali GC, Wu Y-T, Prina M. World Alzheimer Report 2015 - The Global Impact of Dementia: An analysis of prevalence, incidence, cost and trends. 2015; published online Aug. https://kclpure.kcl.ac.uk/portal/en/publications/world-alzheimer-report-2015--the-global-impact-of-dementia(ae525fda-1938-4892-8daa-a2222a672254).html (accessed May 24, 2022).

53 Trautmann S, Rehm J, Wittchen H-U. The economic costs of mental disorders. *EMBO Rep* 2016; **17**: 1245–9.

54 Hatch SL, Mishra G, Hotopf M, Jones PB, Kuh D. Appraisals of stressors and common mental disorder from early to mid-adulthood in the 1946 British birth cohort. *J Affect Disord* 2009; **119**: 66–75.

55 Power C, Kuh D, Morton S. From Developmental Origins of Adult Disease to Life Course Research on Adult Disease and Aging: Insights from Birth Cohort Studies. *Annu Rev Public Health* 2013; **34**: 7–28.

56 Adjei NK, Schlüter DK, Straatmann VS, *et al.* Impact of poverty and family adversity on adolescent health: a multi-trajectory analysis using the UK Millennium Cohort Study. *Lancet Reg Health - Eur* 2022; **13**: 100279.

57 Kuh D, Ben-Shlomo Y, Lynch J, Hallqvist J, Power C. Life course epidemiology. *J Epidemiol Community Health* 2003; **57**: 778–83.

58 Rutter M. Psychosocial Adversity: Risk, Resilience & Recovery. *South Afr J Child Adolesc Ment Health* 1995; **7**: 75–88.

59 Rutter M. Resilience concepts and findings: implications for family therapy. *J Fam Ther* 1999; **21**: 119–44.

For peer review only

For peer review only - http://bmjopen.bmj.com/site/about/guidelines.xhtml

Supplementary materials

Table S1. List of adversity indicators at each stage of the life course.

Adversity variables	N (%)
**Childhood (<17 years)**	
Economic adversity	1353 (63.6%)
Overcrowding	*376 (17.7%)*
Lack of household amenities	*1095 (52.6%)*
Adverse housing condition	*518 (28.9%)*
Paternal occupational status	*167 (8.9%)*
Psychosocial adversity	846 (39.7%)
Separation from mother (>28 days; not due to hospitalisation)	*98 (5.1%)*
Affectionless control parenting	*579 (30.5%)*
Maltreatment	*116 (6.1%)*
Parental divorce	*118 (5.5%)*
Negative peer relationships	*129 (6.8%)*
**Young adulthood (20-36 years)**	
Economic adversity	993 (48.6%)
Overcrowding	*24 (1.3%)*
Lack of household amenities	*185 (9.6%)*
Financial hardships	*323 (20.9%)*
Unemployment	*387 (19.9%)*
Work-related crisis	*289 (15.2%)*
Psychosocial adversity	648 (31.4%)
Social isolation	*569 (29.3%)*
Divorce/separation	*112 (5.5%)*
**Mid-adulthood (43-53 years)**	
Economic adversity	1119 (53.4%)
Financial hardships	*573 (27.4%)*
Work-related crisis	*584 (27.9%)*
Adverse living conditions	*410 (20.3%)*
Psychosocial adversity	1105 (52.7%)
Social isolation	*148 (7.1%)*
Lack of social support	*207 (9.9%)*
Loss of contact with relatives or friends	*269 (12.8%)*
Difficulties with own children	*563 (29.7%)*
Divorce/separation	*355 (16.9%)*
**Late adulthood (63 years)**	
Economic adversity	377 (20.0%)
Financial hardships	*234 (12.7%)*
Work-related crisis	*200 (10.8%)*
Psychosocial adversity	511 (25.8%)
Social isolation	*50 (2.8%)*
Lack of social support	*12 (0.7%)*
Loss of contact with relatives or friends	*99 (5.4%)*
Difficulties with own children	*217 (13.0%)*
Divorce/separation	*209 (11.5%)*

For peer review only - http://bmjopen.bmj.com/site/about/guidelines.xhtml

Causal mediation analysis: Statistical equations

Defining total effect, average direct effect (ADE) and average causal mediation effect (ACME) (adapted from Imai et al., 2011):

Let t = exposure, M = mediator, and Y = outcome. The ACME can be defined using the below formula:

$$\sum\{Y(t, M(t_1)) - Y(t, M(t_0))\}$$

Where ACME equals the difference in outcome due to the change in mediator from the value expected under no exposure ($M(t_0)$) to the value expected under exposure ($M(t_1)$), holding the exposure t at a constant. Fixing the exposure and changing the mediator allows the elimination of other mechanisms and isolates the mediation effect via the mediator.

The ADE can be defined using the following:

$$\sum\{Y(t_1, M(t)) - Y(t_0, M(t))\}$$

Where ADE equals the difference in outcome due to a change in the exposure from not exposed (t_0) to exposed (t_1), holding the mediator at a constant that would be expected under exposure ($M(t)$). This represents the effect of exposure on the outcome that is not hypothesised to be transmitted via the mediator. The total effect equals the sum of the ACME and ADE.

Reference:

Imai K, Keele L, Tingley D, Yamamoto T. Unpacking the Black Box of Causality: Learning about Causal Mechanisms from Experimental and Observational Studies. *Am Polit Sci Rev* 2011; 105: 765–89.

For peer review only - http://bmjopen.bmj.com/site/about/guidelines.xhtml

Table S2. Linear regression models showing the association between duration of exposure to adversity (any, economic, psychosocial) across the lifecourse and cognitive function at 69 years.

Duration of exposure across the lifecourse to:	**Cognitive state (ACE-III)**			**Processing speed**			**Verbal memory**		
	β	95%CI	p-value	β	95%CI	p-value	β	95%CI	p-value
**Any adversity [linear trend]**									
Model 1[a]	**-0.27**	**-0.50; -0.03**	**0.029**	-0.15	-0.39; 0.10	0.237	**-0.33**	**-0.54; -0.11**	**0.003**
Model 2[b]	0.02	-0.19; 0.23	0.829	-0.06	-0.30; 0.18	0.636	-0.07	-0.27; 0.12	0.475
**Economic adversity [linear trend]**									
Model 1[a]	**-0.41**	**-0.57; -0.24**	**<0.001**	-0.14	-0.29; 0.01	0.063	**-0.46**	**-0.61; -0.31**	**<0.001**
Model 2[b]	-0.07	-0.21; 0.08	0.370	-0.04	-0.20; 0.11	0.572	**-0.15**	**-0.28; -0.01**	**0.033**
**Psychosocial adversity [linear trend]**									
Model 1[a]	-0.08	-0.26; 0.10	0.385	-0.09	-0.26; 0.07	0.277	**-0.18**	**-0.33; -0.03**	**0.021**
Model 2[b]	0.00	-0.16; 0.16	0.997	-0.07	-0.23; 0.10	0.418	-0.12	-0.26; 0.02	0.084

[a]Adjusted for sex

[b]Adjusted additionally for educational attainment and childhood (cognition and emotional problems) covariates..

Table S3. Linear regression models showing the asociation between duration of exposure to adversity (any, economic, psychosocial) across the lifecourse and mental health (Psychological distress), and between mental health (psychological distress) and cognitive function at 69 years.

	**Cognitive state (ACE-III)**			**Processing speed**			**Verbal memory**		
	β	95%CI	p-value	β	95%CI	p-value	β	95%CI	p-value
**Any adversity**									
Duration of exposure → Psychological distress	**0.46**	**0.21; 0.71**	**<0.001**	**0.47**	**0.25; 0.70**	**<0.001**	**0.50**	**0.29; 0.72**	**<0.001**
Psychological distress → Cognitive function	**-0.07**	**-0.12; -0.02**	**0.004**	**-0.05**	**-0.10; -0.001**	**0.045**	**-0.07**	**-0.12; -0.03**	**0.001**
**Economic adversity**									
Duration of exposure → Psychological distress	**0.44**	**0.27; 0.61**	**<0001**	**0.43**	**0.26; 0.59**	**<0.001**	**0.46**	**0.28; 0.63**	**<0.001**
Psychological distress → Cognitive function	**-0.07**	**-0.11; -0.02**	**0.006**	**-0.05**	**-0.10; -0.003**	**0.040**	**-0.07**	**-0.11; -0.03**	**0.001**
**Psychosocial adversity**									
Duration of exposure → Psychological distress	**0.51**	**0.32; 0.71**	**<0.001**	**0.48**	**0.31; 0.65**	**<0.001**	**0.49**	**0.31; 0.67**	**<0.001**
Psychological distress → Cognitive function	**-0.07**	**-0.12; -0.02**	**0.005**	-0.05	-0.09; 0.003	0.064	**-0.07**	**-0.11; -0.03**	**0.001**

All models adjusted for socio-demographic (sex, educational attainment) and childhood (cognition and emotional problems) covariates..

For peer review only - http://bmjopen.bmj.com/site/about/guidelines.xhtml

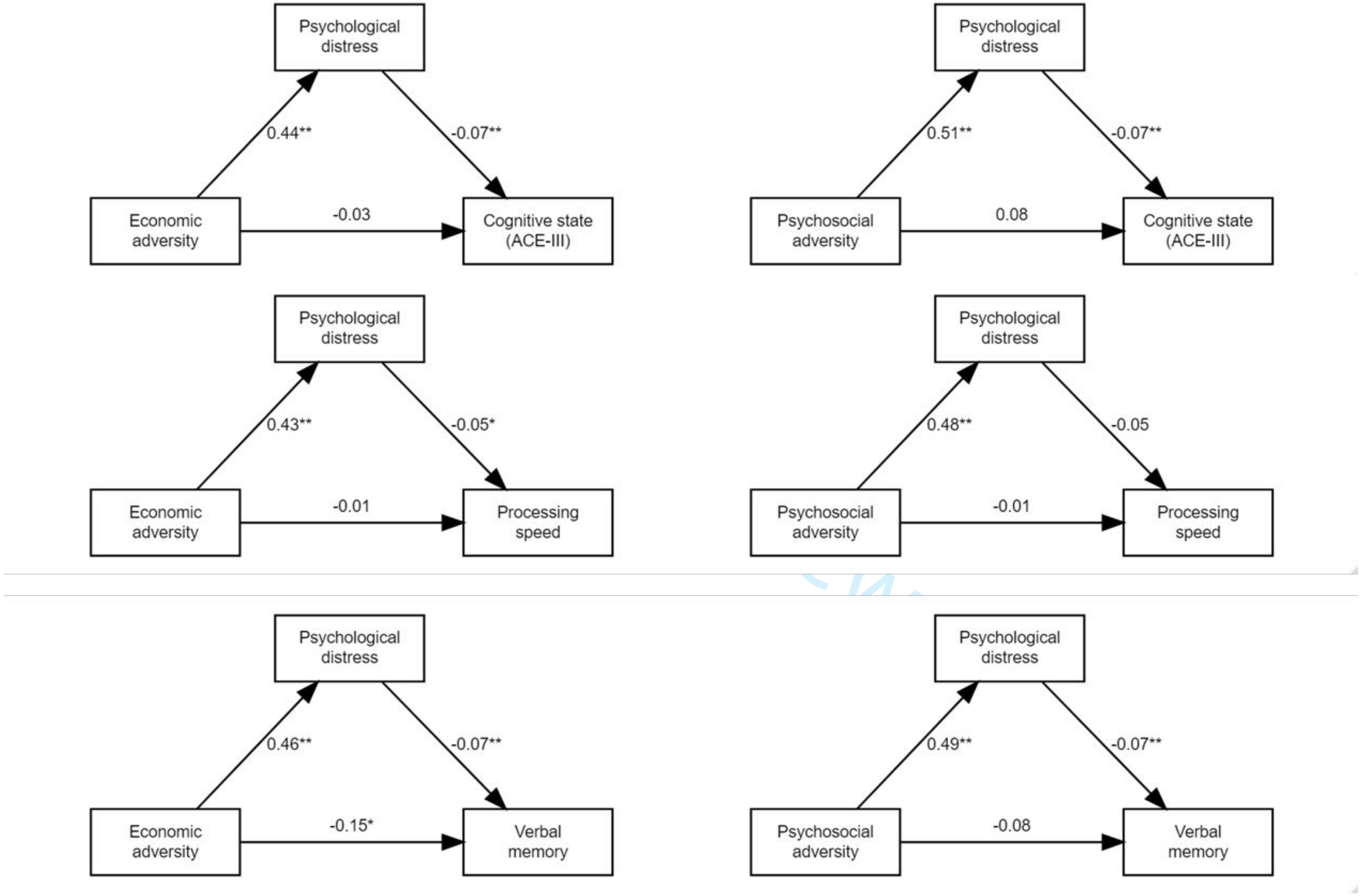

Fig. S1. Mediation models showing the direct and indirect pathways from duration of exposure to economic or psychosocial adversity to cognitive function via mental health (psychological distress). *p<0.05; ** p<0.01

For peer review only - http://bmjopen.bmj.com/site/about/guidelines.xhtml

Table S4. Linear regression models on the interaction between economic and psychosocial adversity on verbal memory at 69 years.

	Verbal memory		
	**β**	**95%CI**	**p-value**
**Interaction model**			
Economic x psychosocial adversity	-0.07	-0.77; 0.64	0.855

For peer review only

For peer review only - http://bmjopen.bmj.com/site/about/guidelines.xhtml

STROBE Statement—Checklist of items that should be included in reports of ***cohort studies***

	Item No	Recommendation	Page No
**Title and abstract**	1	(*a*) Indicate the study's design with a commonly used term in the title or the abstract<br>(*b*) Provide in the abstract an informative and balanced summary of what was done and what was found	1-3
**Introduction**			
Background/rationale	2	Explain the scientific background and rationale for the investigation being reported	4
Objectives	3	State specific objectives, including any prespecified hypotheses	5
**Methods**			
Study design	4	Present key elements of study design early in the paper	6
Setting	5	Describe the setting, locations, and relevant dates, including periods of recruitment, exposure, follow-up, and data collection	6
Participants	6	(*a*) Give the eligibility criteria, and the sources and methods of selection of participants. Describe methods of follow-up<br>(*b*) For matched studies, give matching criteria and number of exposed and unexposed	6
Variables	7	Clearly define all outcomes, exposures, predictors, potential confounders, and effect modifiers. Give diagnostic criteria, if applicable	7-10
Data sources/ measurement	8*	For each variable of interest, give sources of data and details of methods of assessment (measurement). Describe comparability of assessment methods if there is more than one group	7-10
Bias	9	Describe any efforts to address potential sources of bias	11
Study size	10	Explain how the study size was arrived at	6
Quantitative variables	11	Explain how quantitative variables were handled in the analyses. If applicable, describe which groupings were chosen and why	7-10
Statistical methods	12	(*a*) Describe all statistical methods, including those used to control for confounding<br>(*b*) Describe any methods used to examine subgroups and interactions<br>(*c*) Explain how missing data were addressed<br>(*d*) If applicable, explain how loss to follow-up was addressed<br>(*e*) Describe any sensitivity analyses	10-11
**Results**			
Participants	13*	(a) Report numbers of individuals at each stage of study—eg numbers potentially eligible, examined for eligibility, confirmed eligible, included in the study, completing follow-up, and analysed<br>(b) Give reasons for non-participation at each stage<br>(c) Consider use of a flow diagram	6
Descriptive data	14*	(a) Give characteristics of study participants (eg demographic, clinical, social) and information on exposures and potential confounders<br>(b) Indicate number of participants with missing data for each variable of interest<br>(c) Summarise follow-up time (eg, average and total amount)	11-12
Outcome data	15*	Report numbers of outcome events or summary measures over time	12

For peer review only - http://bmjopen.bmj.com/site/about/guidelines.xhtml
For peer review only

Main results	16	(*a*) Give unadjusted estimates and, if applicable, confounder-adjusted estimates and their precision (eg, 95% confidence interval). Make clear which confounders were adjusted for and why they were included<br>(*b*) Report category boundaries when continuous variables were categorized<br>(*c*) If relevant, consider translating estimates of relative risk into absolute risk for a meaningful time period	13-15
Other analyses	17	Report other analyses done—eg analyses of subgroups and interactions, and sensitivity analyses	16
**Discussion**			
Key results	18	Summarise key results with reference to study objectives	16
Limitations	19	Discuss limitations of the study, taking into account sources of potential bias or imprecision. Discuss both direction and magnitude of any potential bias	18-19
Interpretation	20	Give a cautious overall interpretation of results considering objectives, limitations, multiplicity of analyses, results from similar studies, and other relevant evidence	19
Generalisability	21	Discuss the generalisability (external validity) of the study results	19
**Other information**			
Funding	22	Give the source of funding and the role of the funders for the present study and, if applicable, for the original study on which the present article is based	20

*Give information separately for exposed and unexposed groups.

Note: An Explanation and Elaboration article discusses each checklist item and gives methodological background and published examples of transparent reporting. The STROBE checklist is best used in conjunction with this article (freely available on the Web sites of PLoS Medicine at http://www.plosmedicine.org/, Annals of Internal Medicine at http://www.annals.org/, and Epidemiology at http://www.epidem.com/). Information on the STROBE Initiative is available at http://www.strobe-statement.org.

For peer review only

BMJ Open

A lifecourse investigation of the cumulative impact of adversity on cognitive function in old age and the mediating role of mental health: longitudinal birth cohort study

Journal:	*BMJ Open*
Manuscript ID	bmjopen-2023-074105.R1
Article Type:	Original research
Date Submitted by the Author:	28-Jul-2023
Complete List of Authors:	Liu, Yiwen; University College London, MRC Unit for Lifelong Health and Ageing<br>Patalay, Praveetha; University College London, MRC Unit for Lifelong Health and Ageing; University College London, Centre for Longitudinal Studies, Social Research Institute<br>Stafford, Jean; University College London, MRC Unit for Lifelong Health and Ageing<br>Schott, Jonathan M.; University College London, Dementia Research Centre, UCL Queen Square Institute of Neurology<br>Richards, M; University College London, MRC Unit for Lifelong Health and Ageing
**Primary Subject Heading**:	Epidemiology
Secondary Subject Heading:	Mental health, Neurology, Public health
Keywords:	MENTAL HEALTH, Aging, EPIDEMIOLOGY, Dementia

SCHOLARONE™
Manuscripts

I, the Submitting Author has the right to grant and does grant on behalf of all authors of the Work (as defined in the below author licence), an exclusive licence and/or a non-exclusive licence for contributions from authors who are: i) UK Crown employees; ii) where BMJ has agreed a CC-BY licence shall apply, and/or iii) in accordance with the terms applicable for US Federal Government officers or employees acting as part of their official duties; on a worldwide, perpetual, irrevocable, royalty-free basis to BMJ Publishing Group Ltd ("BMJ") its licensees and where the relevant Journal is co-owned by BMJ to the co-owners of the Journal, to publish the Work in this journal and any other BMJ products and to exploit all rights, as set out in our licence.

The Submitting Author accepts and understands that any supply made under these terms is made by BMJ to the Submitting Author unless you are acting as an employee on behalf of your employer or a postgraduate student of an affiliated institution which is paying any applicable article publishing charge ("APC") for Open Access articles. Where the Submitting Author wishes to make the Work available on an Open Access basis (and intends to pay the relevant APC), the terms of reuse of such Open Access shall be governed by a Creative Commons licence – details of these licences and which Creative Commons licence will apply to this Work are set out in our licence referred to above.

Other than as permitted in any relevant BMJ Author's Self Archiving Policies, I confirm this Work has not been accepted for publication elsewhere, is not being considered for publication elsewhere and does not duplicate material already published. I confirm all authors consent to publication of this Work and authorise the granting of this licence.

For peer review only

A lifecourse investigation of the cumulative impact of adversity on cognitive function in old age and the mediating role of mental health: longitudinal birth cohort study

Yiwen Liu[1], Praveetha Patalay[1,2], Jean Stafford[1], Jonathan M Schott[3], Marcus Richards[1]

Affiliations

[1]MRC Unit for Lifelong Health and Ageing at UCL, University College London, London, UK

[2]Centre for Longitudinal Studies, Social Research Institute, University College London, London, UK

[3]Dementia Research Centre, UCL Queen Square Institute of Neurology, London, UK

Corresponding author: Dr Yiwen Liu, MRC Unit for Lifelong Health & Ageing at UCL, University College London, London, UK, WC1E 7HB. Email: eva.liu@ucl.ac.uk

Word count: 3994

For peer review only

Abstract

Objective: To investigate the accumulation of adversities (duration of exposure to any, economic, psychosocial) across the lifecourse (birth to 63 years) on cognitive function in older age, and the mediating role of mental health.

Design: National birth cohort study.

Setting: Great Britain.

Participants: 5362 singleton births within marriage in England, Wales and Scotland born within one week of March 1946, of which 2131 completed at least one cognitive assessment.

Main outcome measures: Cognitive assessments included the Addenbrooke's Cognitive Examination-III (ACE-III), as a measure of cognitive state, processing speed (timed-letter search task), and verbal memory (word learning task) at 69 years. Scores were standardised to the analytical sample. Mental health at 60-64 years was assessed using the 28-item General Health Questionnaire (GHQ-28), with scores standardised to the analytical sample.

Results: After adjusting for sex, increased duration of exposure to any adversity was associated with decreased performance on cognitive state ($\beta = -0.39$; 95%CI [-0.59, -0.20]) and verbal memory ($\beta = -0.45$; 95%CI [-0.63, -0.27]) at 69 years, although these effects were attenuated after adjusting for further covariates (childhood cognition and emotional problems, educational attainment). Analyses by type of adversity revealed stronger associations from economic adversity to verbal memory ($\beta = -0.54$; 95%CI [-0.70, -0.39]), with a small effect remaining even after adjusting for all covariates ($\beta = -0.18$; 95%CI [-0.32, -0.03]), and weaker associations from psychosocial adversity. Causal mediation analyses found that mental health mediated all associations between duration of exposure to adversity (any, economic, psychosocial) and cognitive function, with around 15% of the total effect of economic adversity on verbal memory attributable to mental health.

For peer review only - http://bmjopen.bmj.com/site/about/guidelines.xhtml

Conclusions: Improving mental health among older adults has the potential to reduce cognitive impairments, as well as mitigate against some of the effect of lifecourse accumulation of adversity on cognitive performance in older age.

Strengths and limitations of this study

- Used all prospective indicators of adversity spanning six decades to assess its dose-response effect on cognitive function.
- Adjusted for important covariates in childhood (cognition and mental health) to mitigate against reverse directionality.
- There may be bias from selective drop-out, with those who dropped out having lower childhood cognitive ability and educational attainment.
- The perceived impact or timing of adversities were not considered in this study.

For peer review only

Introduction

Age-related disorders account for almost a quarter of the total burden of diseases worldwide, with cognitive and mental impairments in older age being one of the leading contributors.[1] There is some evidence that exposure to adversity – often assessed using an index that captures a wide array of potentially traumatic events that may be associated with adverse functioning[2] – is associated with reduced cognitive function in older age, particularly for economic adversity such as socio-economic circumstances (SEC), although findings remain mixed and the association between adversity and cognition is likely to be complex.[3–7] Furthermore, few studies have adopted a lifecourse approach to examine prospectively measured adversities accumulated across multiple stages of development. According to the accumulation model – a key lifecourse model – multiple (number of adversities) or persistent exposure (duration of exposure) to adversities can increase dysregulation of the hypothalamic-pituitary-adrenal (HPA)-axis, which over time could lead to physiological damage to neural and somatic systems.[8,9] It is therefore important to investigate whether the accumulation of adversity across the lifecourse is associated with cognitive function in older adulthood to promote healthy cognitive ageing.

The accumulation of adversity can be assessed as the number of adversities experienced (at the same time or across time), or the duration of exposure across time. Experiencing an increased number of adversities (often assessed at one period in time) has been associated with poorer cognitive performance and greater decline in older adulthood.[3,5,10] Similarly, studies which examined the duration of exposure to the same adversity (e.g., SEC, maltreatment) also found an association with cognition in mid- and older adulthood,[6,11,12] although only one study used prospectively collected measures of adversity.[12] These findings suggest that experiencing multiple adversities at the same time or experiencing the same adversity persistently across

For peer review only - http://bmjopen.bmj.com/site/about/guidelines.xhtml

time may both be associated with cognitive impairments in later life. Further research is needed on whether experiencing any adversity (regardless of the type) persistently across the lifecourse (from childhood to older adulthood) also shows a dose-response association with cognitive function in older age.

The accumulation of adversity – as well as having direct effects on cognitive function – may also have indirect effects via mental health. There is consistent evidence that both the number of and duration of exposure to adversities across the lifecourse are associated with increased psychological distress in older adulthood.[13–16] Mental health was also recently identified as one of the leading modifiable risk factors for dementia;[17] both mental health and cognitive function are intertwined across the lifecourse,[18,19] and there is some evidence that mental health in mid-adulthood predicted cognition in older adulthood, but not vice versa.[18] It is therefore plausible that mental health may be a key mechanism that mediates the association between the accumulation of adversity across the lifecourse and cognitive function in older age. There is some research supporting the role of depression as a mediator between one type of adversity (i.e. maltreatment, financial hardships) and cognitive function, and one study which examined the accumulation of adversity (defined as the number of adversities experienced) also identified depression as a partial mediator for cognition.[11,20] However, these studies have mainly relied on retrospective reports – which may be prone to recall biases, or did not examine the duration of exposure to adversity across different stages of the lifecourse. Therefore, further research on the mediating role of mental health is needed using prospective measures of different types of adversity across multiple stages of the lifecourse.

The aim of this study was to examine the longitudinal association between the accumulation of adversity across the lifecourse (defined as the duration of exposure to adversity) and cognitive function in older age, and the mediating role of mental health, using data from the MRC National Survey of Health and Development (NSHD; the British 1946 birth cohort). The

For peer review only - http://bmjopen.bmj.com/site/about/guidelines.xhtml

use of this cohort is particularly advantageous given the long follow-up period, the use of repeated measures across multiple stages of the lifecourse, and the assessment of cognition and mental health in childhood. The latter is especially important for control of potential reverse directionality (i.e. poorer cognitive abilities and mental health in early childhood being associated with increased exposure to adversities across the lifecourse).[21,22] We proposed two primary research questions: first, we examined the duration of exposure to any adversity from birth to 63 years with cognitive function at 69 years; second, we examined to what extent mental health at 60-64 years mediated this relationship. As secondary analyses, we examined economic and psychosocial adversity – two frequently assessed areas of adversity in the literature[23,24] – separately to test for possible specificity.

Methods

Sample

The NSHD originally consisted of 5,362 male and female singleton births within marriage in England, Wales and Scotland, born within one week of March 1946 (http://www.nshd.mrc.ac.uk/nshd). At age 69 a target sample of 2,698 study members who were still alive and had a known current address in mainland Britain were invited to take part in a home visit.[25] Characteristics of those who were not assessed have been reported previously.[25] Ethical approval has been obtained from the Greater Manchester Local Research Ethics Committee and the Scotland Research Ethics Committee. For the latest data collection used in this study, ethical approval was obtained from the NRES Queen Square Research Ethics Committee (14/LO/1073) and Scotland A Research Ethics Committee (14/SS/1009). Participants provided written informed consent at each data collection. The analytical sample included in this study are those who were still alive at 69 years and completed any cognitive assessment (Fig. 1).

For peer review only - http://bmjopen.bmj.com/site/about/guidelines.xhtml

[Figure 1]

Patient and Public Involvement

Patients or the public were not involved in the design, analysis or interpretation of this study.

Measures

Indicators of adversity

Indicators of adversity were broadly grouped into economic and psychosocial domains across four stages of the lifecourse (see supplementary materials: Table S1). Each was coded as binary (presence or absence).

Childhood (<17 years)

Economic: (i) overcrowding ($\geq$2 people per room); (ii) lack of essential household amenities (no sole use of kitchen or bathroom, or no running hot water); (iii) below average housing condition rated by the interviewer; and (iv) father's occupational status (unskilled or unemployed, reported at age 11 or 15 years).

Psychosocial*:* (i) separation from mother for $\geq$28 days (not due to illness or hospitalisation); (ii) parental divorce; or (iii) difficulties with peers (not able to make friends easily or not being popular with other children, rated by the teacher when participants were 13-15 years).

Young adulthood (20-36 years)

Economic*:* (i) overcrowding; (ii) lack of essential household amenities; (iii) financial hardships (going without necessities due to a shortage of money; difficulty managing on current income); or (iv) unemployment.

For peer review only

For peer review only - http://bmjopen.bmj.com/site/about/guidelines.xhtml

Psychosocial: (i) social isolation (never or rarely see friends or family); and (ii) divorce or separation from partner.

Mid-adulthood (43-53 years)

Economic: (i) lost or feared losing employment during the last 12 months; (ii) financial hardships (same items as young adulthood, with an additional item on unable to pay bills); or (iii) inadequate living conditions (no central heating or running hot water, or damp in the property).

Psychosocial: (i) social isolation (never see friends or family; never have people to visit); (ii) lack of social support (no family or friends to talk to; have no one to help in times of crisis); (iii) lost contact with relatives or friends; (iv) difficulties with own children; or (v) divorce or separation from partner.

Late adulthood (60-64 years)

The same indicators from mid-adulthood were used, apart from "inadequate living conditions" in the economic domain, which was not assessed at 60-64 years.

Mental health: Psychological distress (60-64 years)

Mental health at 60-64 years was assessed using the 28-item General Health Questionnaire (GHQ-28), a self-administered questionnaire measuring psychological distress across four areas (somatic symptoms, anxiety and insomnia, social dysfunction and severe depression).[26] Each item was rated on a 4-point Likert scale from 0 (not at all) to 3 (much more than usual), with high internal reliability ($\alpha = 0.91$). A summary score was calculated which ranged from 0 to 84 (higher distress), and scores were standardised to the population.

For peer review only - http://bmjopen.bmj.com/site/about/guidelines.xhtml

Cognitive function (69 years)

Cognitive state

The Addenbrooke's Cognitive Examination-III (ACE-III) was originally developed to screen for mild dementia and cognitive impairments in a clinical setting but is also an appropriate measure of general cognitive state.[27–29] It covers 5 domains of cognitive abilities including attention, memory, language, verbal fluency and visuospatial skills, and was conducted via the ACEmobile app on iPad, with help from the interviewer.[28,29] Scores are calculated for each cognitive domain and an overall score (max=100) was derived from the sum of these domains, with higher scores indicating higher cognitive function.

Processing speed

Processing speed was assessed using a timed-letter search task. Participants were instructed to cross out target letters “P” and “W” embedded among non-target letters as quickly and accurately as possible within 1 minute. A total score representing the speed of letters identified (position reached at the end of the trial) was the main outcome, with a maximum score of 600.

Verbal memory

Verbal memory was assessed using a recall of word learning task. Participants were shown a list of 15 words, each was presented for 2 seconds, and were asked to write down as many as they could remember at the end of the round. This was repeated for 3 rounds, and a total score indicating the number of words correctly recalled across all rounds was calculated, with a maximum score of 45.

For peer review only - http://bmjopen.bmj.com/site/about/guidelines.xhtml

Covariates

Two sets of covariates were included: socio-demographic factors, including sex at birth (male as the reference group), and highest educational attainment by 26 years (categorised into ordinary 'O' level or below vs advanced 'A' level or above, with the latter as the reference group); and childhood measures, including cognitive abilities at 8 years (derived from 4 tests from the National Foundation for Educational Research,[30] standardised to the whole population), and emotional problems at 13-15 years, rated by the teacher and grouped into none (reference group), mild or severe.

For peer review only

Statistical analyses

All statistical analyses were performed in R (version 3.6.2). Scores on each cognitive outcome were standardised to the analytical sample.

Accumulation model

We derived a variable indicating the presence or absence of any adversity at each stage of the lifecourse (childhood, young, mid and late adulthood). These were summed to calculate the duration of exposure to any adversity and ranged from 0 (no exposure) to 4 (exposure at all four times). Using this measure, we tested for a linear dose-response association with cognitive function (cognitive state, processing speed, verbal memory) at 69 years. This was first adjusted for sex, then fully adjusted for all covariates.

Mediation model

Linear regression models first examined the association between the duration of exposure to any adversity and psychological distress at 60-64 years, and between psychological distress at 60-64 years and cognitive functioning at 69 years, adjusted for all covariates. Causal mediation

For peer review only - http://bmjopen.bmj.com/site/about/guidelines.xhtml

analysis then estimated the indirect effect of duration of exposure to any adversity on cognitive function via psychological distress. The R package "mediation" was used, which performs causal mediation analysis within a counterfactual framework.[31–33] The total effect of lifecourse adversity on cognitive function was estimated, which is composed of an average direct effect (ADE), and an average causal mediation effect (ACME) (See supplementary materials for detailed description on the ADE and ACME).[31–34]

Specificity of adversity

We repeated the accumulation and mediation models for economic and psychosocial domains separately to test for possible specificity.

Additional exploratory analysis

We further explored the interaction (tested at $p<0.10$ due to the increased statistical power needed to detect interaction) between economic and psychosocial adversity if both showed associations with cognitive function.

Sensitivity analysis

We further repeated the accumulation and mediation model in a subset of participants with complete data on all three cognitive outcomes (N=1730).

Missing data

Missing data was handled using multivariable imputation by chained equations (MICE) in R[35] with 20 imputed datasets. Imputation was performed on individual indicators of adversity at each stage of the lifecourse, which were included as auxiliary variables along with all variables included in the analysis. Derived adversity variables were calculated post-imputation.

For peer review only - http://bmjopen.bmj.com/site/about/guidelines.xhtml

For peer review only

Imputation was performed up to the maximum analytical sample size for each cognitive outcome, and estimates were combined and pooled together using Rubin's rule.[35]

Results

Sample characteristics

In total, 2,131 participants completed at least one cognitive assessment at 69 years (cognitive state: *N*=1,762; processing speed: *N*=2,111; verbal memory: *N*=2,074). The distribution of adversity variables in the sample can be found in table 1. Economic adversity was more prevalent than psychosocial adversity in childhood, young and mid-adulthood, with roughly half of participants reporting it at each stage. The rates of both adversities were reduced in older adulthood. Across the lifecourse, 4.1% of participants were not exposed to any adversity and 12.7% experienced it persistently (4 times).

Table 1. Sample characteristics.

	**N=2,131**
**Childhood (<17 years)**	
Economic adversity	1353 (63.6%)
Psychosocial adversity	324 (15.2%)
**Young adulthood (20-36 years)**	
Economic adversity	823 (40.3%)
Psychosocial adversity	648 (31.4%)
**Mid-adulthood (43-53 years)**	
Economic adversity	993 (47.4%)
Psychosocial adversity	1105 (52.7%)
**Late adulthood (60-64 years)**	
Economic adversity	316 (16.7%)
Psychosocial adversity	511 (25.8%)
**Duration of exposure across the lifecourse to:**	
**Any adversity**	
0 (None)	88 (4.1%)
1 (Once)	408 (19.1%)
2 (Twice)	739 (34.7%)
3 (Three times)	626 (29.4%)
4 (Four times)	270 (12.7%)
**Economic**	
0 (None)	275 (12.9%)
1 (Once)	718 (33.7%)
2 (Twice)	716 (33.6%)
3 (Three times)	353 (16.6%)
4 (Four times)	69 (3.2%)
**Psychosocial**	

For peer review only - http://bmjopen.bmj.com/site/about/guidelines.xhtml

For peer review only

0 (None)	572 (26.8%)
1 (Once)	786 (36.9%)
2 (Twice)	543 (25.5%)
3 (Three times)	204 (9.6%)
4 (Four times)	26 (1.2%)
**Covariates**	
Sex (Female)	1086 (51.0%)
Educational attainment (26 years; O-levels or below)	1197 (59.3%)
Childhood internalising problems (13-15 years)	
0 (Absent)	1001 (52.4%)
1 (Mild)	713 (37.3%)
2 (Severe)	196 (10.3%)
**Mental health**	
Psychological distress (GHQ-28 total score; max = 84) (M; SD)	16.38 (8.02)
**Cognitive functioning**	
Cognitive state (ACE-III total score; max = 100) (M; SD)	91.52 (6.01)
Processing speed (Max = 600) (M; SD)	262.30 (74.14)
Verbal memory (Max = 45) (M; SD)	22.17 (6.07)

Accumulation model

Increased duration of exposure to any adversity across the lifecourse was associated with lower performance on cognitive state (ACE-III) and verbal memory, but not processing speed, at 69 years. Each additional period of exposure was associated with a 0.4 to 0.5 standard deviation (SD) decrease in cognitive state (β = -0.39; 95%CI [-0.59, -0.20]) and verbal memory (β = -0.45; 95%CI [-0.63, -0.27]) (Fig. 2, supplementary materials: Table S2). However, these effect sizes were attenuated in fully adjusted models.

[Figure 2]

Mediation model

Each increased period of exposure to any adversity across the lifecourse was associated with a 0.46 SD increase in psychological distress, and each SD increase in psychological distress was associated with a 0.07 SD decrease in cognitive state and verbal memory, and a 0.05 SD decrease in processing speed (Fig. 3; supplementary materials: Table S3). Causal mediation analysis found no direct effect of duration of exposure to any adversity on cognitive functioning; however, mediated pathways were found to cognitive state and verbal memory

For peer review only - http://bmjopen.bmj.com/site/about/guidelines.xhtml

via increased psychological distress at 60-64 years, with weaker evidence of a mediated effect found for processing speed (supplementary materials Table S4).

[Figure 3]

Specificity of adversity

Economic adversity

Each additional period of exposure to economic adversity was associated with a 0.51 (95%CI [-0.69, -0.32]), 0.23 (95%CI [-0.40, -0.06]) and 0.54 (95%CI [-0.70, -0.39]) SD decrease in cognitive state, processing speed and verbal memory, respectively (Fig. 2; supplementary materials, Table S2). These were attenuated in fully adjusted models, although a small effect remained on verbal memory ($\beta = -0.18$; 95%CI [-0.32, -0.03]) (Fig. 2; supplementary materials: Table S2). Mediated effects were found to cognitive state and verbal memory via increased psychological distress at 60-64 years, with weaker evidence for a mediated effect to processing speed (supplementary materials: Table S3, S4; Fig.4). Increased exposure to economic adversity showed both a direct and indirect effect on verbal memory, with around 15% of its total effect mediated via psychological distress (calculated as the ratio between total effect and mediated effect).

Psychosocial adversity

A small dose-response effect was found between duration of exposure to psychosocial adversity and verbal memory ($\beta = -0.23$; 95%CI [-0.46, -0.01]), although this became attenuated when adjusted for all covariates (Fig. 2; supplementary materials: Table S2). No other associations were found for cognitive state or processing speed. Causal mediation analysis showed no direct effect of psychosocial adversity, although mediated effects were

For peer review only - http://bmjopen.bmj.com/site/about/guidelines.xhtml

found via psychological distress to both cognitive state and verbal memory, with weaker evidence for mediation on processing speed (supplementary materials Table S3, S4; Fig. 4).

[Figure 4]

Additional exploratory analysis

Given that both economic and psychosocial adversity were associated separately with verbal memory, an interaction term between them was included in the model to examine whether there was evidence for a multiplicative effect, although no interaction was detected ($p = 0.597$) (supplementary materials, Table S4).

Sensitivity analysis

Steps from the accumulation model and mediation model were repeated within a subset of participants with complete data on all three cognitive outcomes, and findings remained the same (supplementary materials Tables S6-7)

Discussion

The overall aim of this paper was to use a lifecourse approach to examine the accumulation of adversity (defined as the duration of exposure) from childhood to adulthood on cognitive function in older adults and estimate the mediating role of mental health on the causal pathway between them. Some support for the dose-response effect of the duration of exposure to adversity was found on cognitive state and verbal memory, but not processing speed. Although most of these effects were attenuated after adjusting for covariates, mediated effects via psychological distress were identified for cognitive state and verbal memory. Stronger associations were found from economic compared to psychosocial adversity to all three

For peer review only - http://bmjopen.bmj.com/site/about/guidelines.xhtml

For peer review only

cognitive outcomes, although similar mediated effects via psychological distress were found regardless of the type of adversity.

The accumulation of adversity can be defined using both the number of adversities experienced or the duration of exposure across time. Although the current study only focused on testing the latter, the dose-response effect of the duration of exposure to adversity on cognitive function is consistent with and expands on previous research which have assessed the number of adversities, offering support for both interpretations of the accumulation model.[3,5,7,12]. However, most of these associations were attenuated after adjusting for educational attainment and childhood characteristics (cognition and emotional problems),[4,36–40] highlighting the importance of having information on these outcomes from earlier in the lifecourse when trying to estimate effects in older adults. Childhood emotional problems are also a predictor of mental health in later life,[41,42] which has been associated with cognition prospectively,[18,19] and may also increase risk of being exposed to further adversities later in life.[43,44] Therefore, although increased duration of exposure to adversity may have a small dose-response effect on cognitive function in older age, most of this can be accounted for by socio-demographic and cognitive and emotional function in childhood.

Although there was weaker evidence for direct effects, mediated effects were found via psychological distress to both cognitive state and verbal memory. Previous research in the same cohort have shown that the accumulation of adversity (both the number of adversities as well as the duration of exposure) is associated with increased psychological distress and decreased wellbeing,[16] and a few studies have also shown an indirect effect of specific types of adversity (e.g., maltreatment, financial hardships) on cognition in adulthood via depressive symptoms.[11,20] However, this is the first study to our knowledge to show prospectively that increased duration of exposure to any adversity across the lifecourse was indirectly associated

For peer review only - http://bmjopen.bmj.com/site/about/guidelines.xhtml

with both cognitive state and specific domains of cognitive function in older adulthood via mental health in older age. One proposed mechanism is that increased psychological distress may lead to over-activation of the HPA-axis and increased cortisol production, which have been associated with cognitive impairments.[45,46] Mental health in older adulthood may therefore be considered as both an antecedent for cognitive impairments later in life as well as a consequence of adversities accumulated across the lifecourse. Given that late-life depression was recently identified as one of the leading modifiable risk factors for dementia,[17] improving mental health in older adults may therefore not only directly improve cognitive function in older age, but could also mitigate some of the effect of lifecourse adversities on cognition.

Different findings also emerged when economic and psychosocial adversities were examined, with stronger associations from economic adversity to verbal memory compared to psychosocial adversity. There is some research suggesting that deprivation (e.g., economic) related adversities may impact more on cognitive processes,[47] possibly via reducing cognitive bandwidth (the mental capacity for paying attention and decision-making) and increasing cognitive load,[48,49] as well as increasing brain atrophy through stress accumulation.[50,51] For example, the hippocampus in particular may be more vulnerable to economic adversity, and the accumulation of economic adversity has been previously associated with increased hippocampal atrophy in mid-to-late life.[50,51] On the other hand, psychosocial adversities may affect emotional reactivity more than cognitive functioning.[47] Although similar mediated effects via psychological distress were found from both types of adversity, this only accounted for a part of the total effect of economic adversity on verbal memory, whereas the effect of psychosocial adversity on verbal memory was wholly mediated, suggesting that mental health may be the primary pathway from psychosocial adversity to cognitive function. Economic adversity further showed a small effect on processing speed, suggesting that processing speed may be more sensitive to economic adversity compared to other types of adversity. One

For peer review only - http://bmjopen.bmj.com/site/about/guidelines.xhtml

potential mechanism is cardiometabolic health, which has been previously associated with both economic adversity and processing speed,[52,53] and may also mediate the association between economic adversity and cognitive function and requires further investigation.[54] Nevertheless, an important possibility is that improving mental health in older adults could help improve cognitive function in the ageing population and mitigate some of the risks associated with persistent adversities. Given the social and health costs associated with cognitive impairments and dementia[55] – combined with the cost of mental ill-health in the population[56] – increasing investment in mental health services may be crucial, in the long term, to promote healthy cognitive and mental ageing.

Strengths and limitations

The longitudinal nature of this study offers several strengths. This is the first study to our knowledge to use prospective indicators of adversity across different lifecourse stages to show the dose-response effect of duration of exposure to adversity on cognitive functioning in late adulthood. The assessment of cognition and mental health in childhood also allowed adjustment for important covariates and helped to mitigate against reverse directionality, although it does not fully exclude this possibility. The inclusion of two different domains of adversity (economic, psychosocial), as well as three different cognitive measures, further allowed testing of specificity in the associations.

There are also some limitations that should be considered. For instance, there may be bias from selective drop-out, as previous research has suggested that those who dropped out were more likely to have lower childhood cognitive ability and educational attainment, and worse mental health.[25] This may have led to an underestimation in the association between exposure to adversity and cognitive functioning. In addition, certain adversities may not be experienced in the same way across different lifecourse stages. For example, childhood economic adversity

For peer review only - http://bmjopen.bmj.com/site/about/guidelines.xhtml

was relatively common in this cohort, given they were born in a time of significant economic uncertainty in a post-war Britain,[57] and may be experienced differently compared to younger birth cohorts. There is some evidence suggesting that the severity of adversity should also be considered in the accumulation model, as adverse events that are perceived as life changing may show stronger effect.[58,59] Caution is also needed in assuming that the same adverse experience is being captured across the lifecourse, particularly as certain indicators of adversity (i.e., difficulties with peers) was reported by the teacher. The lack of ethnic diversity in the cohort also limits the generalisability of these findings, particularly as people from ethnic minority backgrounds are more likely to experience adversities[60] and may be disproportionally affected by them. Furthermore, certain adversities may also co-occur as risk clusters or risk chains,[61–63] and adversities occurring at different stages of the lifecourse may also show differential effects. This was not investigated in the current study given our aim of testing the duration of exposure across the lifecourse, However, there is evidence for a recency effect of adversities on mental health, where later life adversities show stronger associations with mental health compared to adversities experienced earlier in the lifecourse.[58] Further research should therefore focus on assessing the severity as well as the timing of adversities, taking into account risk clusters between different adversities, and to investigate other possible mechanisms such as cardiometabolic health when examining the impact of lifecourse adversity on cognitive ageing.

Conclusion

Overall, findings from this study suggest that increased duration of exposure to any adversity – particularly economic adversity – has a small dose-response effect on cognitive state and verbal memory in older age. Mental health mediated the dose-response effect of duration of exposure to all types of adversity (any, economic, psychosocial) on cognition, suggesting that

For peer review only - http://bmjopen.bmj.com/site/about/guidelines.xhtml

routinely assessing and improving mental health in older adults may also help improve cognitive function in older age. Future studies should also account for the severity, timing, and co-occurrence of adversities, as well as investigate other potential mediators such as cardiometabolic health, to better understand pathways to cognitive ageing.

For peer review only

For peer review only - http://bmjopen.bmj.com/site/about/guidelines.xhtml

Funding: This study is funded by the UK Medical Research Council which provides core funding for the NSHD (MC_UU_00019/1). MR additionally reports funding from MRC (MC_UU_00019/3). JMS acknowledges the support of the National Institute for Health Research University College London Hospitals Biomedical Research Centre, Alzheimer's Research UK, Medical Research Council, UK Dementia Research Institute, Alzheimer's Association, and the British Heart Foundation (PG/17/90/33415). The funders had no involvement in study design, data collection, analysis, interpretation or the decision to submit for publication. The views expressed are those of the author(s) and YL serves as guarantor for the contents of this paper.

Declaration of interest: None

Acknowledgements

We are grateful to all study members who took part in the NSHD and to the study team members who helped to collect the data over the last seven decades.

Author contributions

Conceptualisation: YL, MR, PP

Methodology: YL, PP, JS

Funding acquisition: MR, JMS, PP

Formal analysis: YL

Writing – Original draft: YL

Writing – Review and editing: All authors

Data availability

The data that support the findings of this study are available to bona fide researchers upon request to the NSHD Data Sharing Committee. For more information see: http://www.nshd.mrc.ac.uk/data.

For peer review only

For peer review only - http://bmjopen.bmj.com/site/about/guidelines.xhtml

List of Figures

Fig. 1. Sample flow diagram. *ACE-III: Addenbrooke's Cognitive Examination-III*

Fig. 2. Standardised estimates on the association between duration of exposure to adversity and each cognitive function at 69 years.

Fig. 3. Mediation models showing the direct and indirect effects of lifecourse adversity (duration of exposure to any adversity) on each cognitive function via mental health (psychological distress). *$^*p<0.05$; $^{**}p<0.01$*

Fig. 4. Mediation models showing the direct and indirect pathways from duration of exposure to economic or psychosocial adversity to cognitive function via mental health (psychological distress). *$^*p<0.05$; $^{**}p<0.01$*

For peer review only

References

1 Prince MJ, Wu F, Guo Y, *et al.* The burden of disease in older people and implications for health policy and practice. *The Lancet* 2015; **385**: 549–62.

2 Turner RJ, Lloyd DA. Lifetime traumas and mental health: the significance of cumulative adversity. *J Health Soc Behav* 1995; **36**: 360–76.

3 Chen R, Williams DR, Nishimi K, Slopen N, Kubzansky LD, Weuve J. A life course approach to understanding stress exposures and cognitive function among middle-aged and older adults. *Soc Sci Med* 2022; **314**: 115448.

4 Greenfield EA, Moorman SM. Childhood Socioeconomic Status and Later Life Cognition: Evidence From the Wisconsin Longitudinal Study. *J Aging Health* 2019; **31**: 1589–615.

5 Lee Y, Back JH, Kim J, Byeon H. Multiple Socioeconomic Risks and Cognitive Impairment in Older Adults. *Dement Geriatr Cogn Disord* 2010; **29**: 523–9.

6 Lyu J, Burr JA. Socioeconomic Status Across the Life Course and Cognitive Function Among Older Adults: An Examination of the Latency, Pathways, and Accumulation Hypotheses. *J Aging Health* 2016; **28**: 40–67.

7 Shrira A. Greater age-related decline in markers of physical, mental and cognitive health among Israeli older adults exposed to lifetime cumulative adversity. *Aging Ment Health* 2014; **18**: 610–8.

8 McEwen BS, Rasgon NL. The Brain and Body on Stress: Allostatic Load and Mechanisms for Depression and Dementia. Oxford University Press, 2018 https://oxfordmedicine.com/view/10.1093/med/9780190603342.001.0001/med-9780190603342-chapter-2 (accessed Feb 15, 2022).

9 Power C, Kuh D, Morton S. From Developmental Origins of Adult Disease to Life Course Research on Adult Disease and Aging: Insights from Birth Cohort Studies. *Annu Rev Public Health* 2013; **34**: 7–28.

10 Shrira A. Greater age-related decline in markers of physical, mental and cognitive health among Israeli older adults exposed to lifetime cumulative adversity. *Aging Ment Health* 2014; **18**: 610–8.

11 Künzi M, Gheorghe DA, Kliegel M, Ballhausen N, Gallacher J, Bauermeister S. Cumulative life course adversity, mental health, and cognition in the UK biobank. *Sci Rep* 2022; **12**: 14700.

12 Zeki Al Hazzouri A, Elfassy T, Sidney S, Jacobs D, Pérez Stable EJ, Yaffe K. Sustained Economic Hardship and Cognitive Function: The Coronary Artery Risk Development in Young Adults Study. *Am J Prev Med* 2017; **52**: 1–9.

13 Ejlskov L, Bøggild H, Kuh D, Stafford M. Social relationship adversities throughout the lifecourse and risk of loneliness in later life. *Ageing Soc* 2020; **40**: 1718–34.

14 Hatch SL, Mishra G, Hotopf M, Jones PB, Kuh D. Appraisals of stressors and common mental disorder from early to mid-adulthood in the 1946 British birth cohort. *J Affect Disord* 2009; **119**: 66–75.

For peer review only - http://bmjopen.bmj.com/site/about/guidelines.xhtml

15 Shrira A. The effect of lifetime cumulative adversity on change and chronicity in depressive symptoms and quality of life in older adults. *Int Psychogeriatr* 2012; **24**: 1988–97.

16 Liu Y, Hatch SL, Patalay P, Schott JM, Richards M. A lifecourse approach in examining the association between accumulation of adversity and mental health in older adulthood. *J Affect Disord* 2023; **339**: 211–8.

17 Livingston G, Huntley J, Sommerlad A, *et al.* Dementia prevention, intervention, and care: 2020 report of the Lancet Commission. *The Lancet* 2020; **396**: 413–46.

18 John A, Rusted J, Richards M, Gaysina D. Bidirectional relation between affective symptoms and cognitive function from middle to late adulthood: a population-based birth cohort study. *Aging Ment Health* 2021; **25**: 1642–8.

19 Richards M, Hatch SL. A life course approach to the development of mental skills. *J Gerontol B Psychol Sci Soc Sci* 2011; **66 Suppl 1**: i26-35.

20 Cadar D, Kobayashi LK, Steptoe AP, Demakakos P. OP24 The mediating pathways of the association between adverse childhood experiences and cognitive health in later life. *J Epidemiol Community Health* 2020; **74**: A11–2.

21 Verlinden M, Veenstra R, Ghassabian A, *et al.* Executive Functioning and Non-Verbal Intelligence as Predictors of Bullying in Early Elementary School. *J Abnorm Child Psychol* 2014; **42**: 953–66.

22 Zych I, Farrington DP, Llorent VJ, Ribeaud D, Eisner MP. Childhood Risk and Protective Factors as Predictors of Adolescent Bullying Roles. *Int J Bullying Prev* 2021; **3**: 138–46.

23 Lawn RB, Anderson EL, Suderman M, *et al.* Psychosocial adversity and socioeconomic position during childhood and epigenetic age: analysis of two prospective cohort studies. *Hum Mol Genet* 2018; **27**: 1301–8.

24 Sousa ACP de A, Guerra RO, Tu MT, Phillips SP, Guralnik JM, Zunzunegui M-V. Lifecourse Adversity and Physical Performance across Countries among Men and Women Aged 65-74. *PLOS ONE* 2014; **9**: e102299.

25 Kuh D, Wong A, Shah I, *et al.* The MRC National Survey of Health and Development reaches age 70: maintaining participation at older ages in a birth cohort study. *Eur J Epidemiol* 2016; **31**: 1135–47.

26 Goldberg DP, Hillier VF. A scaled version of the General Health Questionnaire. *Psychol Med* 1979; **9**: 139–45.

27 Noone P. Addenbrooke's Cognitive Examination-III. *Occup Med* 2015; **65**: 418–20.

28 James S-N, Davis D, O'Hare C, *et al.* Lifetime affective problems and later-life cognitive state: Over 50 years of follow-up in a British birth cohort study. *J Affect Disord* 2018; **241**: 348–55.

29 Richards M, James S-N, Sizer A, *et al.* Identifying the lifetime cognitive and socioeconomic antecedents of cognitive state: seven decades of follow-up in a British birth cohort study. *BMJ Open* 2019; **9**: e024404.

30 Pigeon DA. Tests used in the 1954 and 1957 surveys. *Home Sch* 1964; : 129–32.

For peer review only - http://bmjopen.bmj.com/site/about/guidelines.xhtml

31 Imai K, Keele L, Tingley D, Yamamoto T. Unpacking the Black Box of Causality: Learning about Causal Mechanisms from Experimental and Observational Studies. *Am Polit Sci Rev* 2011; **105**: 765–89.

32 Imai K, Keele L, Tingley D. A general approach to causal mediation analysis. *Psychol Methods* 2010; **15**: 309–34.

33 Imai K, Keele L, Yamamoto T. Identification, Inference and Sensitivity Analysis for Causal Mediation Effects. *Stat Sci* 2010; **25**: 51–71.

34 Tingley D, Yamamoto T, Hirose K, Keele LJ, Imai K. mediation: R Package for Causal Mediation Analysis. *J Stat Softw* 2014; **59**: 1–38.

35 Buuren SV, Groothuis-oudshoorn K. MICE: Multivariate imputation by chained equations in R. *J Stat Softw* 2011; **45**: 1–67.

36 Kuiper JS, Smidt N, Zuidema SU, Comijs HC, Oude Voshaar RC, Zuidersma M. A longitudinal study of the impact of social network size and loneliness on cognitive performance in depressed older adults. *Aging Ment Health* 2020; **24**: 889–97.

37 Richards M, Wadsworth MEJ. Long term effects of early adversity on cognitive function. *Arch Dis Child* 2004; **89**: 922–7.

38 Staff RT, Chapko D, Hogan MJ, Whalley LJ. Life course socioeconomic status and the decline in information processing speed in late life. *Soc Sci Med* 2016; **151**: 130–8.

39 Ding R, He P. Associations between childhood adversities and late-life cognitive function: Potential mechanisms. *Soc Sci Med* 2021; **291**: 114478.

40 Halpin AB, MacAulay RK, Boeve AR, D’Errico LM, Michaud S. Are Adverse Childhood Experiences Associated with Worse Cognitive Function in Older Adults? *J Int Neuropsychol Soc* 2021; : 1–10.

41 Colman I, Wadsworth MEJ, Croudace TJ, Jones PB. Forty-Year Psychiatric Outcomes Following Assessment for Internalizing Disorder in Adolescence. *Am J Psychiatry* 2007; **164**: 126–33.

42 Copeland WE, Alaie I, Jonsson U, Shanahan L. Associations of Childhood and Adolescent Depression With Adult Psychiatric and Functional Outcomes. *J Am Acad Child Adolesc Psychiatry* 2021; **60**: 604–11.

43 Groot S de, Veldman K, Iii BCA, Bültmann U. Work functioning among young adults: the role of mental health problems from childhood to young adulthood. *Occup Environ Med* 2022; **79**: 217–23.

44 Stansfeld SA, Clark C, Caldwell T, Rodgers B, Power C. Psychosocial work characteristics and anxiety and depressive disorders in midlife: the effects of prior psychological distress. *Occup Environ Med* 2008; **65**: 634–42.

45 Keller J, Gomez R, Williams G, *et al.* HPA axis in major depression: cortisol, clinical symptomatology and genetic variation predict cognition. *Mol Psychiatry* 2017; **22**: 527–36.

46 Wingenfeld K, Wolf OT. HPA Axis Alterations in Mental Disorders: Impact on Memory and its Relevance for Therapeutic Interventions. *CNS Neurosci Ther* 2011; **17**: 714–22.

For peer review only - http://bmjopen.bmj.com/site/about/guidelines.xhtml

47 Sheridan MA, Shi F, Miller AB, Salhi C, McLaughlin KA. Network structure reveals clusters of associations between childhood adversities and development outcomes. *Dev Sci* 2020; **23**: e12934.

48 Mani A, Mullainathan S, Shafir E, Zhao J. Poverty Impedes Cognitive Function. *Science* 2013; **341**: 976–80.

49 Shah AK, Mullainathan S, Shafir E. Some Consequences of Having Too Little. *Science* 2012; **338**: 682–5.

50 Butterworth P, Cherbuin N, Sachdev P, Anstey KJ. The association between financial hardship and amygdala and hippocampal volumes: results from the PATH through life project. *Soc Cogn Affect Neurosci* 2012; **7**: 548–56.

51 Elbejjani M, Fuhrer R, Abrahamowicz M, *et al.* Life-Course Socioeconomic Position and Hippocampal Atrophy in a Prospective Cohort of Older Adults. *Psychosom Med* 2017; **79**: 14–23.

52 Pollitt RA, Rose KM, Kaufman JS. Evaluating the evidence for models of life course socioeconomic factors and cardiovascular outcomes: a systematic review. *BMC Public Health* 2005; **5**: 7.

53 Lyall DM, Celis-Morales CA, Anderson J, *et al.* Associations between single and multiple cardiometabolic diseases and cognitive abilities in 474 129 UK Biobank participants. *Eur Heart J* 2017; **38**: 577–83.

54 Oi K, Haas S. Cardiometabolic Risk and Cognitive Decline: The Role of Socioeconomic Status in Childhood and Adulthood. *J Health Soc Behav* 2019; **60**: 326–43.

55 Prince MJ, Wimo A, Guerchet MM, Ali GC, Wu Y-T, Prina M. World Alzheimer Report 2015 - The Global Impact of Dementia: An analysis of prevalence, incidence, cost and trends. 2015; published online Aug. https://kclpure.kcl.ac.uk/portal/en/publications/world-alzheimer-report-2015--the-global-impact-of-dementia(ae525fda-1938-4892-8daa-a2222a672254).html (accessed May 24, 2022).

56 Trautmann S, Rehm J, Wittchen H-U. The economic costs of mental disorders. *EMBO Rep* 2016; **17**: 1245–9.

57 Short JR. Housing in Britain: The Post-War Experience. London: Routledge, 2021 DOI:10.4324/9781003158066.

58 Hatch SL, Mishra G, Hotopf M, Jones PB, Kuh D. Appraisals of stressors and common mental disorder from early to mid-adulthood in the 1946 British birth cohort. *J Affect Disord* 2009; **119**: 66–75.

59 Power C, Kuh D, Morton S. From Developmental Origins of Adult Disease to Life Course Research on Adult Disease and Aging: Insights from Birth Cohort Studies. *Annu Rev Public Health* 2013; **34**: 7–28.

60 Adjei NK, Schlüter DK, Straatmann VS, *et al.* Impact of poverty and family adversity on adolescent health: a multi-trajectory analysis using the UK Millennium Cohort Study. *Lancet Reg Health - Eur* 2022; **13**: 100279.

61 Kuh D, Ben-Shlomo Y, Lynch J, Hallqvist J, Power C. Life course epidemiology. *J Epidemiol Community Health* 2003; **57**: 778–83.

For peer review only - http://bmjopen.bmj.com/site/about/guidelines.xhtml

62 Rutter M. Psychosocial Adversity: Risk, Resilience & Recovery. *South Afr J Child Adolesc Ment Health* 1995; **7**: 75–88.

63 Rutter M. Resilience concepts and findings: implications for family therapy. *J Fam Ther* 1999; **21**: 119–44.

For peer review only

For peer review only - http://bmjopen.bmj.com/site/about/guidelines.xhtml

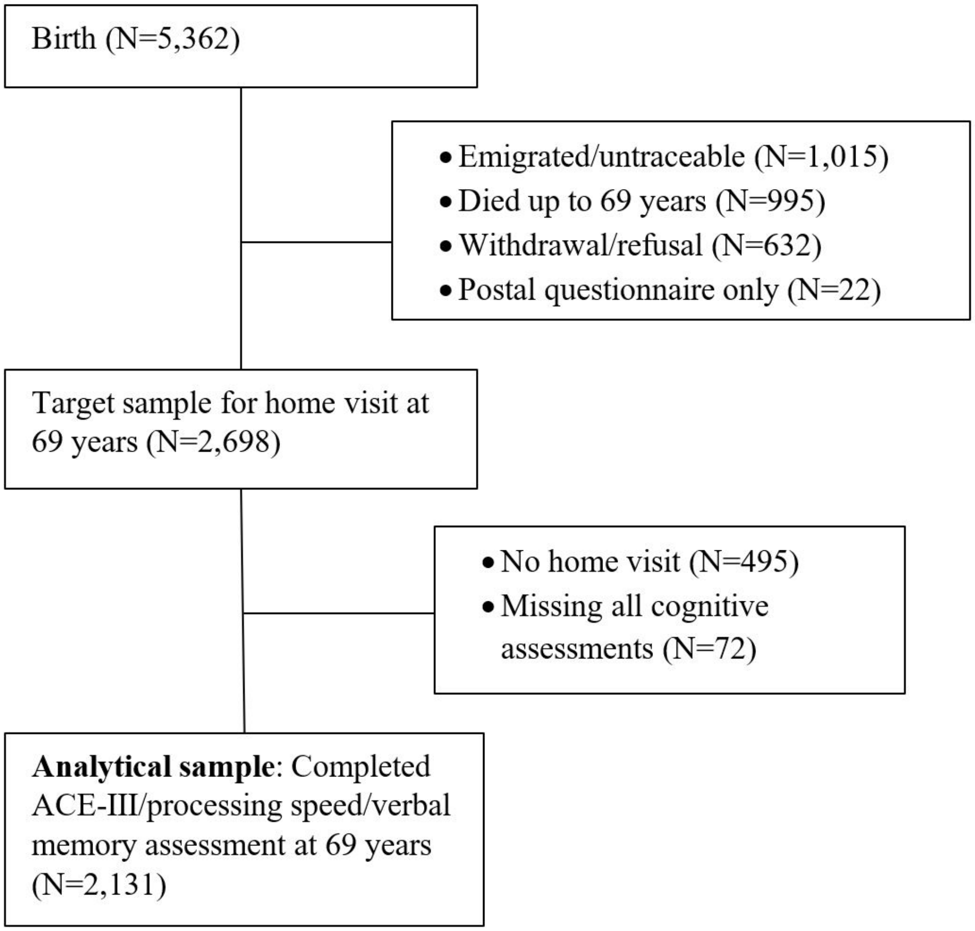

Fig. 1. Sample flow diagram. ACE-III: Addenbrooke's Cognitive Examination-III.

224x220mm (120 x 120 DPI)

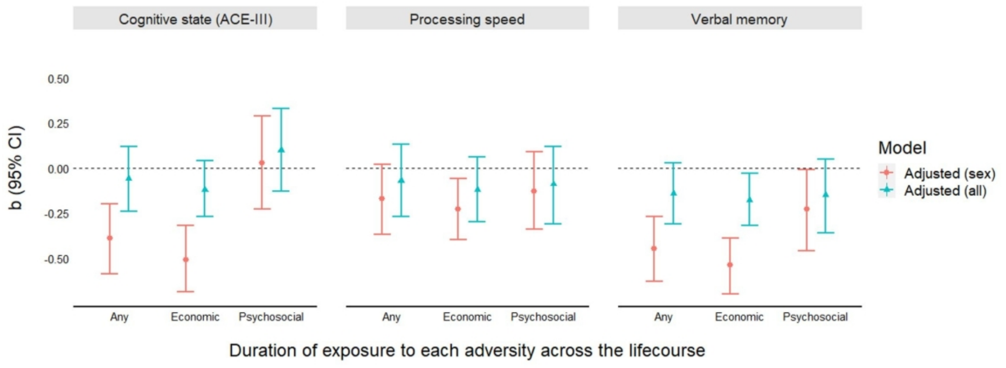

Fig. 2. Standardised estimates on the association between duration of exposure to adversity and each cognitive function at 69 years.

437x168mm (120 x 120 DPI)

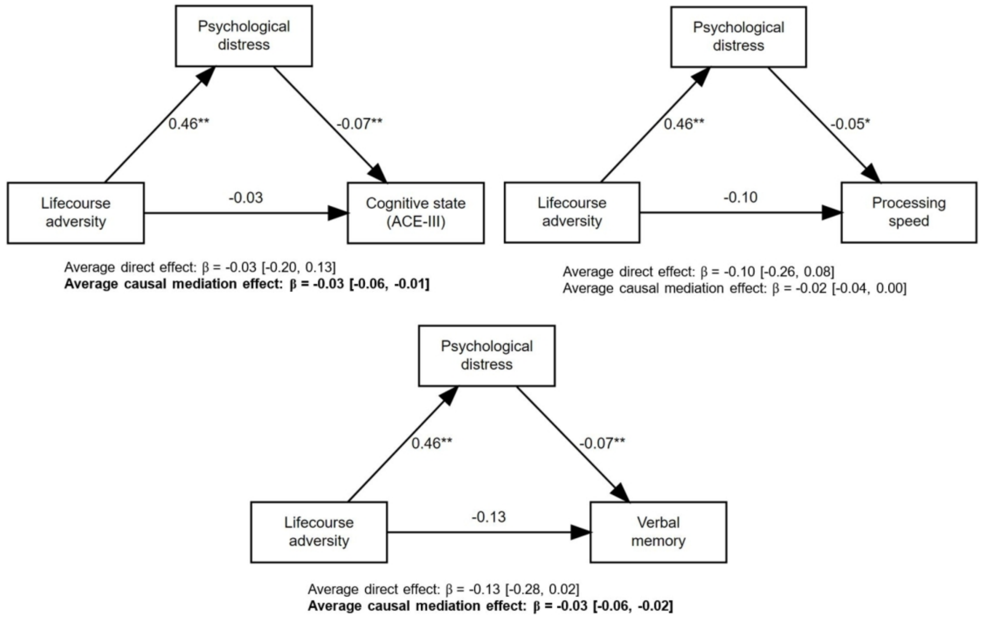

Fig. 3. Mediation models showing the direct and indirect effects of lifecourse adversity (duration of exposure to any adversity) on each cognitive function via mental health (psychological distress). **p<0.05; ** p<0.01*

404x250mm (120 x 120 DPI)

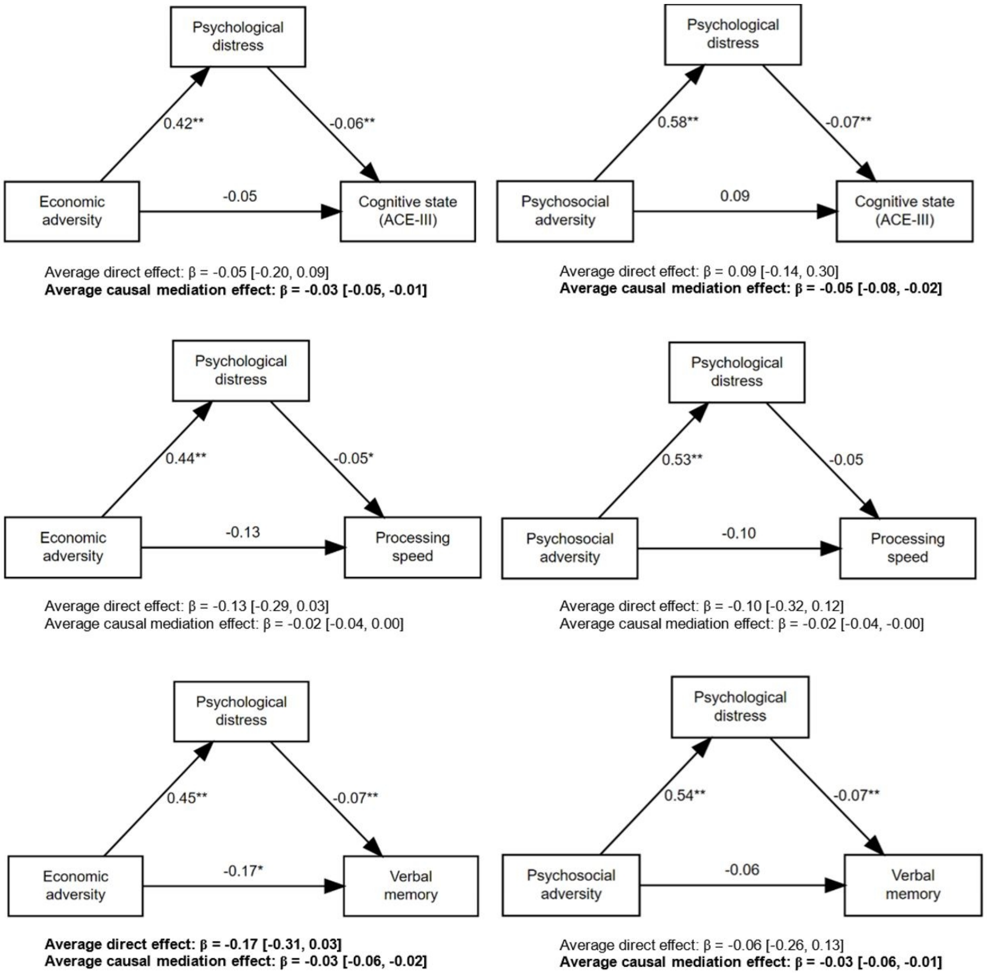

Fig. 4. Mediation models showing the direct and indirect pathways from duration of exposure to economic or psychosocial adversity to cognitive function via mental health (psychological distress). **p<0.05; ** p<0.01*

264x256mm (120 x 120 DPI)

Supplementary materials

Table S1. List of adversity indicators at each stage of the life course.

Adversity variables	N (%)
**Childhood (<17 years)**	
Economic adversity	1353 (63.6%)
Overcrowding	*376 (17.7%)*
Lack of household amenities	*1095 (52.6%)*
Adverse housing condition	*518 (28.9%)*
Paternal occupational status	*167 (8.9%)*
Psychosocial adversity	324 (15.2%)
Separation from mother (>28 days; not due to hospitalisation)	*98 (5.1%)*
Parental divorce	*118 (5.5%)*
Negative peer relationships	*129 (6.8%)*
**Young adulthood (20-36 years)**	
Economic adversity	823 (40.3%)
Overcrowding	*24 (1.3%)*
Lack of household amenities	*185 (9.6%)*
Financial hardships	*323 (20.9%)*
Unemployment	*387 (19.9%)*
Psychosocial adversity	648 (31.4%)
Social isolation	*569 (29.3%)*
Divorce/separation	*112 (5.5%)*
**Mid-adulthood (43-53 years)**	
Economic adversity	993 (47.4%)
Financial hardships	*573 (27.4%)*
Work-related crisis	*358 (17.1%)*
Adverse living conditions	*410 (20.3%)*
Psychosocial adversity	1105 (52.7%)
Social isolation	*148 (7.1%)*
Lack of social support	*207 (9.9%)*
Loss of contact with relatives or friends	*269 (12.8%)*
Difficulties with own children	*563 (29.7%)*
Divorce/separation	*355 (16.9%)*
**Late adulthood (63 years)**	
Economic adversity	316 (16.7%)
Financial hardships	*234 (12.7%)*
Work-related crisis	*113 (6.1%)*
Psychosocial adversity	511 (25.8%)
Social isolation	*50 (2.8%)*
Lack of social support	*12 (0.7%)*
Loss of contact with relatives or friends	*99 (5.4%)*
Difficulties with own children	*217 (13.0%)*
Divorce/separation	*209 (11.5%)*

For peer review only

For peer review only - http://bmjopen.bmj.com/site/about/guidelines.xhtml

Causal mediation analysis: Statistical equations

Defining total effect, average direct effect (ADE) and average causal mediation effect (ACME) (adapted from Imai et al., 2011):

Let t = exposure, M = mediator, and Y = outcome. The ACME can be defined using the below formula:

$$\sum\{Y(t, M(t_1)) - Y(t, M(t_0))\}$$

Where ACME equals the difference in outcome due to the change in mediator from the value expected under no exposure (M (t_0)) to the value expected under exposure (M (t_1)), holding the exposure t at a constant. Fixing the exposure and changing the mediator allows the elimination of other mechanisms and isolates the mediation effect via the mediator.

The ADE can be defined using the following:

$$\sum\{Y(t_1, M(t)) - Y(t_0, M(t))\}$$

Where ADE equals the difference in outcome due to a change in the exposure from not exposed (t_0) to exposed (t_1), holding the mediator at a constant that would be expected under exposure (M (t)). This represents the effect of exposure on the outcome that is not hypothesised to be transmitted via the mediator. The total effect equals the sum of the ACME and ADE.

Reference:

Imai K, Keele L, Tingley D, Yamamoto T. Unpacking the Black Box of Causality: Learning about Causal Mechanisms from Experimental and Observational Studies. *Am Polit Sci Rev* 2011; 105: 765–89.

For peer review only

Table S2. Linear regression models showing the association between duration of exposure to adversity (any, economic, psychosocial) across the lifecourse and cognitive function at 69 years.

Duration of exposure across the lifecourse to:	Cognitive state (ACE-III)			Processing speed			Verbal memory		
	β	95%CI	p-value	β	95%CI	p-value	β	95%CI	p-value
**Any adversity [linear trend]**									
Model 1[a]	**-0.39**	**-0.59; -0.20**	**<0.001**	-0.17	-0.37; 0.02	0.084	**-0.45**	**-0.63; -0.27**	**<0.001**
Model 2[b]	-0.06	-0.24; 0.12	0.510	-0.07	-0.27; 0.13	0.476	-0.14	-0.31; 0.03	0.102
**Economic adversity [linear trend]**									
Model 1[a]	**-0.51**	**-0.69; -0.32**	**<0.001**	**-0.23**	**-0.40; -0.06**	**0.010**	**-0.54**	**-0.70; -0.39**	**<0.001**
Model 2[b]	-0.12	-0.27; 0.04	0.147	-0.12	-0.30; 0.06	0.195	**-0.18**	**-0.32; -0.03**	**0.016**
**Psychosocial adversity [linear trend]**									
Model 1[a]	0.03	-0.23; 0.29	0.834	-0.13	-0.34; 0.09	0.255	**-0.23**	**-0.46; -0.01**	**0.044**
Model 2[b]	0.10	-0.13; 0.33	0.397	-0.09	-0.31; 0.12	0.402	-0.15	-0.36; 0.05	0.143

[a]Adjusted for sex
[b]Adjusted additionally for educational attainment and childhood (cognition and emotional problems) covariates.

Table S3. Linear regression models showing the association between duration of exposure to adversity (any, economic, psychosocial) across the lifecourse and mental health (Psychological distress), and between mental health (psychological distress) and cognitive function at 69 years.

	Cognitive state (ACE-III)			Processing speed			Verbal memory		
	β	95%CI	p-value	β	95%CI	p-value	β	95%CI	p-value
**Any adversity**									
Duration of exposure → Psychological distress	**0.46**	**0.26; 0.66**	**<0.001**	**0.46**	**0.28; 0.63**	**<0.001**	**0.46**	**0.28; 0.64**	**<0.001**
Psychological distress → Cognitive function	**-0.07**	**-0.11; -0.02**	**0.005**	**-0.05**	**-0.10; -0.004**	**0.035**	**-0.07**	**-0.11; -0.03**	**0.001**
**Economic adversity**									
Duration of exposure → Psychological distress	**0.42**	**0.21; 0.63**	**<0.001**	**0.44**	**0.26; 0.61**	**<0.001**	**0.45**	**0.27; 0.64**	**<0.001**
Psychological distress → Cognitive function	**-0.06**	**-0.11; -0.02**	**0.007**	**-0.05**	**-0.10; -0.003**	**0.038**	**-0.07**	**-0.11; -0.03**	**0.001**
**Psychosocial adversity**									
Duration of exposure → Psychological distress	**0.58**	**0.31; 0.84**	**<0.001**	**0.53**	**0.30; 0.76**	**<0.001**	**0.54**	**0.29; 0.80**	**<0.001**
Psychological distress → Cognitive function	**-0.07**	**-0.12; -0.02**	**0.004**	-0.05	-0.10; 0.00	0.051	**-0.07**	**-0.11; -0.03**	**0.001**

All models adjusted for socio-demographic (sex, educational attainment) and childhood (cognition and emotional problems) covariates.

For peer review only - http://bmjopen.bmj.com/site/about/guidelines.xhtml

Table S4. Causal mediation analysis showing the average direct, mediated (via mental health), and total effect of duration of exposure to adversity (any, economic, psychosocial) on cognitive function at 69 years.

	Cognitive state (ACE-III)		Processing speed		Verbal memory	
	β	95%CI	β	95%CI	β	95%CI
**Any adversity**						
ADE	-0.03	-0.20; 0.13	-0.10	-0.26; 0.08	-0.13	-0.28; 0.02
ACME	**-0.03**	**-0.06; -0.01**	-0.02	-0.04, 0.00	**-0.03**	**-0.06; -0.02**
Total effect	-0.07	-0.23; 0.09	-0.12	-0.28; 0.05	**-0.16**	**-0.31; -0.02**
**Economic**						
ADE	-0.05	-0.20; 0.09	-0.13	-0.29; 0.03	**-0.17**	**-0.31; -0.03**
ACME	**-0.03**	**-0.05; -0.01**	-0.02	-0.04; 0.00	**-0.03**	**-0.06; -0.02**
Total effect	-0.08	-0.23; 0.06	-0.14	-0.31; 0.01	**-0.20**	**-0.34; -0.07**
**Psychosocial**						
ADE	0.09	-0.14; 0.30	-0.10	-0.32; 0.12	-0.06	-0.26; 0.13
ACME	**-0.05**	**-0.08; -0.02**	-0.02	-0.04; 0.00	**-0.03**	**-0.06; -0.01**
Total effect	0.04	-0.18; 0.25	-0.11	-0.34; 0.10	-0.10	-0.30; 0.09

Adjusted for socio-demographic (sex, educational attainment) and childhood (cognition and emotional problems) covariates. Abbreviations: ADE: average direct effect; ACME: average causal mediation effect.

Table S5. Linear regression models on the interaction between economic and psychosocial adversity on verbal memory at 69 years.

	Verbal memory		
	**β**	**95%CI**	**p-value**
**Interaction model**			
Economic x psychosocial adversity	-0.79	-3.73; 2.15	0.597

For peer review only - http://bmjopen.bmj.com/site/about/guidelines.xhtml

For peer review only

Sensitivity analyses

Repeating steps from main analysis for a subset of participants with complete data on all three cognitive measures (N=1720)

Table S6. Linear regression models showing the association between duration of exposure to adversity (any, economic, psychosocial) across the lifecourse and cognitive function at 69 years (sensitivity analyses).

Duration of exposure across the lifecourse to:	Cognitive state (ACE-III)			Processing speed			Verbal memory		
	β	95%CI	p-value	β	95%CI	p-value	β	95%CI	p-value
**Any adversity [linear trend]**									
Model 1[a]	**-0.37**	**-0.55; -0.18**	**<0.001**	-0.17	-0.37; 0.04	0.107	**-0.46**	**-0.66; -0.27**	**<0.001**
Model 2[b]	-0.03	-0.19; 0.13	0.716	-0.06	-0.27; 0.15	0.579	-0.15	-0.33; 0.02	0.091

[a]Adjusted for sex

[b]Adjusted additionally for educational attainment and childhood (cognition and emotional problems) covariates.

Table S7. Causal mediation analysis showing the average direct, mediated (via mental health), and total effect of duration of exposure to adversity (any, economic, psychosocial) on cognitive function at 69 years.

	Cognitive state (ACE-III)		Processing speed		Verbal memory	
	β	95%CI	β	95%CI	β	95%CI
**Any adversity**						
ADE	0.01	-0.14; 0.16	0.01	-0.17; 0.20	-0.11	-0.28; 0.05
ACME	**-0.03**	**-0.06; -0.01**	-0.02	-0.05; 0.00	**-0.04**	**-0.06; -0.02**
Total effect	-0.02	-0.18; 0.13	-0.01	-0.20; 0.17	-0.15	-0.31; 0.01

Adjusted for socio-demographic (sex, educational attainment) and childhood (cognition and emotional problems) covariates. Abbreviations: ADE: average direct effect; ACME: average causal mediation effect.

For peer review only

STROBE Statement—Checklist of items that should be included in reports of ***cohort studies***

	Item No	Recommendation	Page No
**Title and abstract**	1	(*a*) Indicate the study's design with a commonly used term in the title or the abstract<br>(*b*) Provide in the abstract an informative and balanced summary of what was done and what was found	1-3
**Introduction**			
Background/rationale	2	Explain the scientific background and rationale for the investigation being reported	4-5
Objectives	3	State specific objectives, including any prespecified hypotheses	5-6
**Methods**			
Study design	4	Present key elements of study design early in the paper	6
Setting	5	Describe the setting, locations, and relevant dates, including periods of recruitment, exposure, follow-up, and data collection	6
Participants	6	(*a*) Give the eligibility criteria, and the sources and methods of selection of participants. Describe methods of follow-up<br>(*b*) For matched studies, give matching criteria and number of exposed and unexposed	6-7
Variables	7	Clearly define all outcomes, exposures, predictors, potential confounders, and effect modifiers. Give diagnostic criteria, if applicable	7-10
Data sources/ measurement	8*	For each variable of interest, give sources of data and details of methods of assessment (measurement). Describe comparability of assessment methods if there is more than one group	7-10
Bias	9	Describe any efforts to address potential sources of bias	11
Study size	10	Explain how the study size was arrived at	6
Quantitative variables	11	Explain how quantitative variables were handled in the analyses. If applicable, describe which groupings were chosen and why	7-10
Statistical methods	12	(*a*) Describe all statistical methods, including those used to control for confounding<br>(*b*) Describe any methods used to examine subgroups and interactions<br>(*c*) Explain how missing data were addressed<br>(*d*) If applicable, explain how loss to follow-up was addressed<br>(*e*) Describe any sensitivity analyses	10-12
**Results**			
Participants	13*	(a) Report numbers of individuals at each stage of study—eg numbers potentially eligible, examined for eligibility, confirmed eligible, included in the study, completing follow-up, and analysed<br>(b) Give reasons for non-participation at each stage<br>(c) Consider use of a flow diagram	6-7
Descriptive data	14*	(a) Give characteristics of study participants (eg demographic, clinical, social) and information on exposures and potential confounders<br>(b) Indicate number of participants with missing data for each variable of interest<br>(c) Summarise follow-up time (eg, average and total amount)	12-13
Outcome data	15*	Report numbers of outcome events or summary measures over time	13

For peer review only

For peer review only - http://bmjopen.bmj.com/site/about/guidelines.xhtml

Main results	16	(*a*) Give unadjusted estimates and, if applicable, confounder-adjusted estimates and their precision (eg, 95% confidence interval). Make clear which confounders were adjusted for and why they were included<br>(*b*) Report category boundaries when continuous variables were categorized<br>(*c*) If relevant, consider translating estimates of relative risk into absolute risk for a meaningful time period	14-16
Other analyses	17	Report other analyses done—eg analyses of subgroups and interactions, and sensitivity analyses	18
**Discussion**			
Key results	18	Summarise key results with reference to study objectives	18
Limitations	19	Discuss limitations of the study, taking into account sources of potential bias or imprecision. Discuss both direction and magnitude of any potential bias	21-22
Interpretation	20	Give a cautious overall interpretation of results considering objectives, limitations, multiplicity of analyses, results from similar studies, and other relevant evidence	20-21
Generalisability	21	Discuss the generalisability (external validity) of the study results	21-22
**Other information**			
Funding	22	Give the source of funding and the role of the funders for the present study and, if applicable, for the original study on which the present article is based	23

*Give information separately for exposed and unexposed groups.

Note: An Explanation and Elaboration article discusses each checklist item and gives methodological background and published examples of transparent reporting. The STROBE checklist is best used in conjunction with this article (freely available on the Web sites of PLoS Medicine at http://www.plosmedicine.org/, Annals of Internal Medicine at http://www.annals.org/, and Epidemiology at http://www.epidem.com/). Information on the STROBE Initiative is available at http://www.strobe-statement.org.

For peer review only

For peer review only - http://bmjopen.bmj.com/site/about/guidelines.xhtml

BMJ Open

A lifecourse investigation of the cumulative impact of adversity on cognitive function in old age and the mediating role of mental health: longitudinal birth cohort study

Journal:	*BMJ Open*
Manuscript ID	bmjopen-2023-074105.R2
Article Type:	Original research
Date Submitted by the Author:	10-Oct-2023

Complete List of Authors:	Liu, Yiwen; University College London, MRC Unit for Lifelong Health and Ageing<br>Patalay, Praveetha; University College London, MRC Unit for Lifelong Health and Ageing; University College London, Centre for Longitudinal Studies, Social Research Institute<br>Stafford, Jean; University College London, MRC Unit for Lifelong Health and Ageing<br>Schott, Jonathan M.; University College London, Dementia Research Centre, UCL Queen Square Institute of Neurology<br>Richards, M; University College London, MRC Unit for Lifelong Health and Ageing

**Primary Subject Heading**:	Epidemiology
Secondary Subject Heading:	Mental health, Neurology, Public health
Keywords:	MENTAL HEALTH, Aging, EPIDEMIOLOGY, Dementia

SCHOLARONE™
Manuscripts

I, the Submitting Author has the right to grant and does grant on behalf of all authors of the Work (as defined in the below author licence), an exclusive licence and/or a non-exclusive licence for contributions from authors who are: i) UK Crown employees; ii) where BMJ has agreed a CC-BY licence shall apply, and/or iii) in accordance with the terms applicable for US Federal Government officers or employees acting as part of their official duties; on a worldwide, perpetual, irrevocable, royalty-free basis to BMJ Publishing Group Ltd ("BMJ") its licensees and where the relevant Journal is co-owned by BMJ to the co-owners of the Journal, to publish the Work in this journal and any other BMJ products and to exploit all rights, as set out in our licence.

The Submitting Author accepts and understands that any supply made under these terms is made by BMJ to the Submitting Author unless you are acting as an employee on behalf of your employer or a postgraduate student of an affiliated institution which is paying any applicable article publishing charge ("APC") for Open Access articles. Where the Submitting Author wishes to make the Work available on an Open Access basis (and intends to pay the relevant APC), the terms of reuse of such Open Access shall be governed by a Creative Commons licence – details of these licences and which Creative Commons licence will apply to this Work are set out in our licence referred to above.

Other than as permitted in any relevant BMJ Author's Self Archiving Policies, I confirm this Work has not been accepted for publication elsewhere, is not being considered for publication elsewhere and does not duplicate material already published. I confirm all authors consent to publication of this Work and authorise the granting of this licence.

For peer review only

A lifecourse investigation of the cumulative impact of adversity on cognitive function in old age and the mediating role of mental health: longitudinal birth cohort study

Yiwen Liu[1], Praveetha Patalay[1,2], Jean Stafford[1], Jonathan M Schott[3], Marcus Richards[1]

Affiliations

[1]MRC Unit for Lifelong Health and Ageing at UCL, University College London, London, UK

[2]Centre for Longitudinal Studies, Social Research Institute, University College London, London, UK

[3]Dementia Research Centre, UCL Queen Square Institute of Neurology, London, UK

Corresponding author: Dr Yiwen Liu, MRC Unit for Lifelong Health & Ageing at UCL, University College London, London, UK, WC1E 7HB. Email: eva.liu@ucl.ac.uk

Word count: 3994

For peer review only

Abstract

Objective: To investigate the accumulation of adversities (duration of exposure to any, economic, psychosocial) across the lifecourse (birth to 63 years) on cognitive function in older age, and the mediating role of mental health.

Design: National birth cohort study.

Setting: Great Britain.

Participants: 5362 singleton births within marriage in England, Wales and Scotland born within one week of March 1946, of which 2131 completed at least one cognitive assessment.

Main outcome measures: Cognitive assessments included the Addenbrooke's Cognitive Examination-III (ACE-III), as a measure of cognitive state, processing speed (timed-letter search task), and verbal memory (word learning task) at 69 years. Scores were standardised to the analytical sample. Mental health at 60-64 years was assessed using the 28-item General Health Questionnaire (GHQ-28), with scores standardised to the analytical sample.

Results: After adjusting for sex, increased duration of exposure to any adversity was associated with decreased performance on cognitive state (β = -0.39; 95%CI [-0.59, -0.20]) and verbal memory (β = -0.45; 95%CI [-0.63, -0.27]) at 69 years, although these effects were attenuated after adjusting for further covariates (childhood cognition and emotional problems, educational attainment). Analyses by type of adversity revealed stronger associations from economic adversity to verbal memory (β = -0.54; 95%CI [-0.70, -0.39]), with a small effect remaining even after adjusting for all covariates (β = -0.18; 95%CI [-0.32, -0.03]), and weaker associations from psychosocial adversity. Causal mediation analyses found that mental health mediated all associations between duration of exposure to adversity (any, economic, psychosocial) and cognitive function, with around 15% of the total effect of economic adversity on verbal memory attributable to mental health.

For peer review only - http://bmjopen.bmj.com/site/about/guidelines.xhtml

Conclusions: Improving mental health among older adults has the potential to reduce cognitive impairments, as well as mitigate against some of the effect of lifecourse accumulation of adversity on cognitive performance in older age.

Strengths and limitations of this study

- Used all prospective indicators of adversity spanning six decades to assess its dose-response effect on cognitive function.
- Adjusted for important covariates in childhood (cognition and mental health) to mitigate against reverse directionality.
- There may be bias from selective drop-out, with those who dropped out having lower childhood cognitive ability and educational attainment.
- The perceived impact or timing of adversities were not considered in this study.

For peer review only

For peer review only - http://bmjopen.bmj.com/site/about/guidelines.xhtml

Introduction

Age-related disorders account for almost a quarter of the total burden of diseases worldwide, with cognitive and mental impairments in older age being one of the leading contributors.[1] There is some evidence that exposure to adversity – often assessed using an index that captures a wide array of potentially traumatic events that may be associated with adverse functioning [2] – is associated with reduced cognitive function in older age, particularly for economic adversity such as socio-economic circumstances (SEC), although findings remain mixed and the association between adversity and cognition is likely to be complex.[3–7] Furthermore, few studies have adopted a lifecourse approach to examine prospectively measured adversities accumulated across multiple stages of development. According to the accumulation model – a key lifecourse model – multiple (number of adversities) or persistent exposure (duration of exposure) to adversities can increase dysregulation of the hypothalamic-pituitary-adrenal (HPA)-axis, which over time could lead to physiological damage to neural and somatic systems.[8,9] It is therefore important to investigate whether the accumulation of adversity across the lifecourse is associated with cognitive function in older adulthood to better understand pathways to cognitive ageing.

The4ccumulateion of adversity can be assessed as the number of adversities experienced (at the same time or across time), or the duration of exposure across time. Experiencing an increased number of adversities (often assessed at one period in time) has been associated with poorer cognitive performance and greater decline in older adulthood.[3,5,10] Similarly, studies which examined the duration of exposure to the same adversity (e.g., SEC, maltreatment) also found an association between increased duration of exposure and worse cognition in mid- and older adulthood,[6,11,12] although only one study used prospectively collected measures of adversity.[12] These findings suggest that experiencing multiple adversities at the same time

For peer review only - http://bmjopen.bmj.com/site/about/guidelines.xhtml

or experiencing the same adversity persistently across time may both be associated with cognitive impairments in later life. Further research is needed on whether experiencing any adversity (regardless of the type) persistently across the lifecourse (from childhood to older adulthood) also shows a dose-response association with poorer cognitive function in older age.

The accumulation of adversity – as well as having direct effects on cognitive function – may also have indirect effects via mental health. There is consistent evidence that both the number of and duration of exposure to adversities across the lifecourse are associated with increased psychological distress in older adulthood.[13–16] Mental ill-health was also recently identified as one of the leading modifiable risk factors for dementia;[17] both mental health and cognitive function are intertwined across the lifecourse,[18,19] and there is some evidence that mental health in mid-adulthood predicted cognition in older adulthood, but not vice versa.[18] It is therefore plausible that mental health may be a key mechanism that mediates the association between the accumulation of adversity across the lifecourse and cognitive function in older age. There is some research supporting the role of depression as a mediator between one type of adversity (i.e. maltreatment, financial hardships) and cognitive function, and one study which examined the accumulation of adversity (defined as the number of adversities experienced) also identified depression as a partial mediator for cognition.[11,20] However, these studies have mainly relied on retrospective reports – which may be prone to recall biases, or did not examine the duration of exposure to adversity across different stages of the lifecourse. Therefore, further research on the mediating role of mental health is needed using prospective measures of different types of adversity across multiple stages of the lifecourse.

The aim of this study was to examine the longitudinal association between the accumulation of adversity across the lifecourse (defined as the duration of exposure to adversity) and cognitive function in older age, and the mediating role of mental health, using data from the MRC National Survey of Health and Development (NSHD; the British 1946 birth cohort). The

For peer review only - http://bmjopen.bmj.com/site/about/guidelines.xhtml

use of this cohort is particularly advantageous given the long follow-up period, the use of repeated measures across multiple stages of the lifecourse, and the assessment of cognition and mental health in childhood. The latter is especially important for control of potential reverse directionality (i.e. poorer cognitive abilities and mental health in early childhood being associated with increased exposure to adversities across the lifecourse).[21,22] We proposed two primary research questions: first, we examined the duration of exposure to any adversity from birth to 63 years with cognitive function at 69 years; second, we examined to what extent mental health at 60-64 years mediated this relationship. As secondary analyses, we examined economic and psychosocial adversity – two frequently assessed areas of adversity in the literature [23,24] – separately to test for possible specificity.

Methods

Sample

The NSHD originally consisted of 5,362 male and female singleton births within marriage in England, Wales and Scotland, born within one week of March 1946 (http://www.nshd.mrc.ac.uk/nshd). At age 69 a target sample of 2,698 study members who were still alive and had a known current address in mainland Britain were invited to take part in a home visit.[25] Characteristics of those who were not assessed have been reported previously.[25] Ethical approval has been obtained from the Greater Manchester Local Research Ethics Committee and the Scotland Research Ethics Committee. For the latest data collection used in this study, ethical approval was obtained from the NRES Queen Square Research Ethics Committee (14/LO/1073) and Scotland A Research Ethics Committee (14/SS/1009). Participants provided written informed consent at each data collection. The analytical sample included in this study are those who were still alive at 69 years and completed any cognitive assessment (Fig. 1).

For peer review only - http://bmjopen.bmj.com/site/about/guidelines.xhtml

[Figure 1]

Patient and Public Involvement

Patients or the public were not involved in the design, analysis or interpretation of this study.

Measures

Indicators of adversity

Indicators of adversity were broadly grouped into economic and psychosocial domains across four stages of the lifecourse (see supplementary materials: Table S1). Each was coded as binary (presence or absence).

<u>*Childhood (<17 years)*</u>

<u>Economic</u>: (i) overcrowding (≥2 people per room); (ii) lack of essential household amenities (no sole use of kitchen or bathroom, or no running hot water); (iii) below average housing condition rated by the interviewer; and (iv) father's occupational status (unskilled or unemployed, reported at age 11 or 15 years).

<u>Psychosocial</u>*:* (i) separation from mother for ≥28 days (not due to illness or hospitalisation); (ii) parental divorce; or (iii) difficulties with peers (not able to make friends easily or not being popular with other children, rated by the teacher when participants were 13-15 years).

<u>*Young adulthood (20-36 years)*</u>

<u>Economic</u>*:* (i) overcrowding; (ii) lack of essential household amenities; (iii) financial hardships (going without necessities due to a shortage of money; difficulty managing on current income); or (iv) unemployment.

For peer review only - http://bmjopen.bmj.com/site/about/guidelines.xhtml

Psychosocial: (i) social isolation (never or rarely see friends or family); and (ii) divorce or separation from partner.

Mid-adulthood (43-53 years)

Economic: (i) lost or feared losing employment during the last 12 months; (ii) financial hardships (same items as young adulthood, with an additional item on unable to pay bills); or (iii) inadequate living conditions (no central heating or running hot water, or damp in the property).

Psychosocial: (i) social isolation (never see friends or family; never have people to visit); (ii) lack of social support (no family or friends to talk to; have no one to help in times of crisis); (iii) lost contact with relatives or friends; (iv) difficulties with own children; or (v) divorce or separation from partner.

Late adulthood (60-64 years)

The same indicators from mid-adulthood were used, apart from "inadequate living conditions" in the economic domain, which was not assessed at 60-64 years.

Mental health: Psychological distress (60-64 years)

Mental health at 60-64 years was assessed using the 28-item General Health Questionnaire (GHQ-28), a self-administered questionnaire measuring psychological distress across four areas (somatic symptoms, anxiety and insomnia, social dysfunction and severe depression).[26] Each item was rated on a 4-point Likert scale from 0 (not at all) to 3 (much more than usual), with high internal reliability ($\alpha = 0.91$). A summary score was calculated which ranged from 0 to 84 (higher distress), and scores were standardised to the population.

For peer review only - http://bmjopen.bmj.com/site/about/guidelines.xhtml

For peer review only

Cognitive function (69 years)

Cognitive state

The Addenbrooke's Cognitive Examination-III (ACE-III) was originally developed to screen for mild dementia and cognitive impairments in a clinical setting but is also an appropriate measure of general cognitive state.[27–29] It covers 5 domains of cognitive abilities including attention, memory, language, verbal fluency and visuospatial skills, and was conducted via the ACEmobile app on iPad, with help from the interviewer.[28,29] Scores are calculated for each cognitive domain and an overall score (max=100) was derived from the sum of these domains, with higher scores indicating higher cognitive function.

Processing speed

Processing speed was assessed using a timed-letter search task. Participants were instructed to cross out target letters "P" and "W" embedded among non-target letters as quickly and accurately as possible within 1 minute. A total score representing the speed of letters identified (position reached at the end of the trial) was the main outcome, with a maximum score of 600.

Verbal memory

Verbal memory was assessed using a recall of word learning task. Participants were shown a list of 15 words, each was presented for 2 seconds, and were asked to write down as many as they could remember at the end of the round. This was repeated for 3 rounds, and a total score indicating the number of words correctly recalled across all rounds was calculated, with a maximum score of 45.

For peer review only - http://bmjopen.bmj.com/site/about/guidelines.xhtml

For peer review only

Covariates

Two sets of covariates were included: socio-demographic factors, including sex at birth (male as the reference group), and highest educational attainment by 26 years (categorised into ordinary 'O' level or below vs advanced 'A' level or above, with the latter as the reference group); and childhood measures, including cognitive abilities at 8 years (derived from 4 tests from the National Foundation for Educational Research,[30] standardised to the whole population), and emotional problems at 13-15 years, rated by the teacher and grouped into none (reference group), mild or severe.

Statistical analyses

All statistical analyses were performed in R (version 3.6.2). Scores on each cognitive outcome were standardised to the analytical sample.

Accumulation model

We derived a variable indicating the presence or absence of any adversity at each stage of the lifecourse (childhood, young, mid and late adulthood). These were summed to calculate the duration of exposure to any adversity and ranged from 0 (no exposure) to 4 (exposure at all four times). Using this measure, we tested for a linear dose-response association with cognitive function (cognitive state, processing speed, verbal memory) at 69 years. This was first adjusted for sex, then fully adjusted for all covariates.

Mediation model

Linear regression models first examined the association between the duration of exposure to any adversity and psychological distress at 60-64 years, and between psychological distress at 60-64 years and cognitive functioning at 69 years, adjusted for all covariates. Causal mediation

For peer review only - http://bmjopen.bmj.com/site/about/guidelines.xhtml

analysis then estimated the indirect effect of duration of exposure to any adversity on cognitive function via psychological distress. The R package "mediation" was used, which performs causal mediation analysis within a counterfactual framework.[31–33] The total effect of lifecourse adversity on cognitive function was estimated, which is composed of an average direct effect (ADE), and an average causal mediation effect (ACME) (See supplementary materials for detailed description on the ADE and ACME).[31–34]

Specificity of adversity

We repeated the accumulation and mediation models for economic and psychosocial domains separately to test for possible specificity.

Additional exploratory analysis

We further explored the interaction (tested at $p<0.10$ due to the increased statistical power needed to detect interaction) between economic and psychosocial adversity if both showed associations with cognitive function.

Sensitivity analysis

We further repeated the accumulation and mediation model in a subset of participants with complete data on all three cognitive outcomes (N=1730).

Missing data

Missing data was handled using multivariable imputation by chained equations (MICE) in R [35] with 20 imputed datasets. Imputation was performed on individual indicators of adversity at each stage of the lifecourse, which were included as auxiliary variables along with all variables included in the analysis. Derived adversity variables were calculated post-imputation.

For peer review only - http://bmjopen.bmj.com/site/about/guidelines.xhtml

For peer review only

Imputation was performed up to the maximum analytical sample size for each cognitive outcome, and estimates were combined and pooled together using Rubin's rule.[35]

Results

Sample characteristics

In total, 2,131 participants completed at least one cognitive assessment at 69 years (cognitive state: *N*=1,762; processing speed: *N*=2,111; verbal memory: *N*=2,074). The distribution of adversity variables in the sample can be found in table 1. Economic adversity was more prevalent than psychosocial adversity in childhood, young and mid-adulthood, with roughly half of participants reporting it at each stage. The rates of both adversities were reduced in older adulthood. Across the lifecourse, 4.1% of participants were not exposed to any adversity and 12.7% experienced it persistently (4 times).

Table 1. Sample characteristics.

	N=2,131
**Childhood (<17 years)**	
Economic adversity	1353 (63.6%)
Psychosocial adversity	324 (15.2%)
**Young adulthood (20-36 years)**	
Economic adversity	823 (40.3%)
Psychosocial adversity	648 (31.4%)
**Mid-adulthood (43-53 years)**	
Economic adversity	993 (47.4%)
Psychosocial adversity	1105 (52.7%)
**Late adulthood (60-64 years)**	
Economic adversity	316 (16.7%)
Psychosocial adversity	511 (25.8%)
**Duration of exposure across the lifecourse to:**	
**Any adversity**	
0 (None)	88 (4.1%)
1 (Once)	408 (19.1%)
2 (Twice)	739 (34.7%)
3 (Three times)	626 (29.4%)
4 (Four times)	270 (12.7%)
**Economic**	
0 (None)	275 (12.9%)
1 (Once)	718 (33.7%)
2 (Twice)	716 (33.6%)
3 (Three times)	353 (16.6%)
4 (Four times)	69 (3.2%)
**Psychosocial**	

For peer review only - http://bmjopen.bmj.com/site/about/guidelines.xhtml

0 (None)	572 (26.8%)
1 (Once)	786 (36.9%)
2 (Twice)	543 (25.5%)
3 (Three times)	204 (9.6%)
4 (Four times)	26 (1.2%)
**Covariates**	
Sex (Female)	1086 (51.0%)
Educational attainment (26 years; O-levels or below)	1197 (59.3%)
Childhood internalising problems (13-15 years)	
0 (Absent)	1001 (52.4%)
1 (Mild)	713 (37.3%)
2 (Severe)	196 (10.3%)
**Mental health**	
Psychological distress (GHQ-28 total score; max = 84) (M; SD)	16.38 (8.02)
**Cognitive functioning**	
Cognitive state (ACE-III total score; max = 100) (M; SD)	91.52 (6.01)
Processing speed (Max = 600) (M; SD)	262.30 (74.14)
Verbal memory (Max = 45) (M; SD)	22.17 (6.07)

Accumulation model

Increased duration of exposure to any adversity across the lifecourse was associated with lower performance on cognitive state (ACE-III) and verbal memory, but not processing speed, at 69 years. Each additional period of exposure was associated with a 0.4 to 0.5 standard deviation (SD) decrease in cognitive state (β = -0.39; 95%CI [-0.59, -0.20]) and verbal memory (β = -0.45; 95%CI [-0.63, -0.27]) (Fig. 2, supplementary materials: Table S2). However, these effect sizes were attenuated in fully adjusted models.

[Figure 2]

Mediation model

Each increased period of exposure to any adversity across the lifecourse was associated with a 0.46 SD increase in psychological distress, and each SD increase in psychological distress was associated with a 0.07 SD decrease in cognitive state and verbal memory, and a 0.05 SD decrease in processing speed (Fig. 3; supplementary materials: Table S3). Causal mediation analysis found no direct effect of duration of exposure to any adversity on cognitive functioning; however, mediated pathways were found to cognitive state and verbal memory via

For peer review only - http://bmjopen.bmj.com/site/about/guidelines.xhtml

increased psychological distress at 60-64 years, with weaker evidence of a mediated effect found for processing speed (supplementary materials Table S4).

[Figure 3]

Specificity of adversity

Economic

Each additional period of exposure to economic adversity was associated with a 0.51 (95%CI [-0.69, -0.32]), 0.23 (95%CI [-0.40, -0.06]) and 0.54 (95%CI [-0.70, -0.39]) SD decrease in cognitive state, processing speed and verbal memory, respectively (Fig. 2; supplementary materials, Table S2). These were attenuated in fully adjusted models, although a small effect remained on verbal memory ($\beta = -0.18$; 95%CI [-0.32, -0.03]) (Fig. 2; supplementary materials: Table S2). Mediated effects were found to cognitive state and verbal memory via increased psychological distress at 60-64 years, with weaker evidence for a mediated effect to processing speed (supplementary materials: Table S3, S4; Fig.4). Increased exposure to economic adversity showed both a direct and indirect effect on verbal memory, with around 15% of its total effect mediated via psychological distress (calculated as the ratio between total effect and mediated effect).

Psychosocial

A small dose-response effect was found between duration of exposure to psychosocial adversity and verbal memory ($\beta = -0.23$; 95%CI [-0.46, -0.01]), although this became attenuated when adjusted for all covariates (Fig. 2; supplementary materials: Table S2). No other associations were found for cognitive state or processing speed. Causal mediation analysis showed no direct effect of psychosocial adversity, although mediated effects were

For peer review only - http://bmjopen.bmj.com/site/about/guidelines.xhtml

found via psychological distress to both cognitive state and verbal memory, with weaker evidence for mediation on processing speed (supplementary materials Table S3, S4; Fig. 4).

[Figure 4]

Additional exploratory analysis

Given that both economic and psychosocial adversity were associated separately with verbal memory, an interaction term between them was included in the model to examine whether there was evidence for a multiplicative effect, although no interaction was detected ($p = 0.597$) (supplementary materials, Table S4).

Sensitivity analysis

Steps from the accumulation model and mediation model were repeated within a subset of participants with complete data on all three cognitive outcomes, and findings remained the same (supplementary materials Tables S6-7)

Discussion

The overall aim of this paper was to use a lifecourse approach to examine the accumulation of adversity (defined as the duration of exposure) from childhood to adulthood on cognitive function in older adults and estimate the mediating role of mental health on the causal pathway between them. Some support for the dose-response effect of the duration of exposure to adversity was found on cognitive state and verbal memory, but not processing speed. Although most of these effects were attenuated after adjusting for covariates, mediated effects via psychological distress were identified for cognitive state and verbal memory. Stronger associations were found from economic compared to psychosocial adversity to all three

For peer review only - http://bmjopen.bmj.com/site/about/guidelines.xhtml

For peer review only

cognitive outcomes, although similar mediated effects via psychological distress were found regardless of the type of adversity.

The accumulation of adversity can be defined using both the number of adversities experienced or the duration of exposure across time. Although the current study only focused on testing the latter, the dose-response effect of the duration of exposure to adversity on poorer cognitive function is consistent with and expands on previous research which have assessed the number of adversities, offering support for both interpretations of the accumulation model.[3,5,7,12] However, most of these associations were attenuated after adjusting for educational attainment and childhood characteristics (cognition and emotional problems),[4,36–40] highlighting the importance of having information on these outcomes from earlier in the lifecourse when trying to estimate effects in older adults. Childhood emotional problems are also a predictor of mental ill-health in later life,[41,42] which has been associated with cognition prospectively,[18,19] and may also increase risk of being exposed to further adversities later in life.[43,44] Therefore, although increased duration of exposure to adversity may have a small dose-response effect on poorer cognitive function in older age, most of this can be accounted for by socio-demographic and cognitive and emotional function in childhood.

Although there was weaker evidence for direct effects, mediated effects were found via psychological distress to both cognitive state and verbal memory. Previous research in the same cohort have shown that the accumulation of adversity (both the number of adversities as well as the duration of exposure) is associated with increased psychological distress and decreased wellbeing,[16] and a few studies have also shown an indirect effect of specific types of adversity (e.g., maltreatment, financial hardships) on cognition in adulthood via depressive symptoms.[11,20] However, this is the first study to our knowledge to show prospectively that increased duration of exposure to any adversity across the lifecourse was indirectly associated

For peer review only - http://bmjopen.bmj.com/site/about/guidelines.xhtml

with both cognitive state and specific domains of cognitive function in older adulthood via mental health in older age. One proposed mechanism is that increased psychological distress may lead to over-activation of the HPA-axis and increased cortisol production, which have been associated with cognitive impairments.[45,46] Mental ill-health in older adulthood may therefore be considered as both an antecedent for cognitive impairments later in life as well as a consequence of adversities accumulated across the lifecourse. Given that late-life depression was recently identified as one of the leading modifiable risk factors for dementia,[17] improving mental health in older adults may therefore not only directly improve cognitive function in older age, but could also mitigate some of the effect of lifecourse adversities on cognition.

Different findings also emerged when economic and psychosocial adversities were examined, with stronger associations from economic adversity to verbal memory compared to psychosocial adversity. There is some research suggesting that deprivation (e.g., economic) related adversities may impact more on cognitive processes,[47] possibly via reducing cognitive bandwidth (the mental capacity for paying attention and decision-making) and increasing cognitive load,[48,49] as well as increasing brain atrophy through stress accumulation.[50,51] For example, the hippocampus in particular may be more vulnerable to economic adversity, and the accumulation of economic adversity has been previously associated with increased hippocampal atrophy in mid-to-late life.[50,51] On the other hand, psychosocial adversities may affect emotional reactivity more than cognitive functioning.[47] Although similar mediated effects via psychological distress were found from both types of adversity, this only accounted for a part of the total effect of economic adversity on verbal memory, whereas the effect of increased psychosocial adversity on poorer verbal memory was wholly mediated via poorer mental health, suggesting that mental ill-health may be the primary pathway from psychosocial adversity to worse cognitive function. Economic adversity further

For peer review only - http://bmjopen.bmj.com/site/about/guidelines.xhtml

showed a small effect on processing speed, suggesting that processing speed may be more sensitive to economic adversity compared to other types of adversity. One potential mechanism is cardiometabolic health, which has been previously associated with both economic adversity and processing speed,[52,53] and may also mediate the association between economic adversity and cognitive function and requires further investigation.[54] Nevertheless, an important possibility is that improving mental health in older adults could help improve cognitive function in the ageing population and mitigate some of the risks associated with persistent adversities. Given the social and health costs associated with cognitive impairments and dementia [55] – combined with the cost of mental ill-health in the population [56] – increasing investment in mental health services may be crucial, in the long term, to promote healthy cognitive and mental ageing.

Strengths and limitations

The longitudinal nature of this study offers several strengths. This is the first study to our knowledge to use prospective indicators of adversity across different lifecourse stages to show the dose-response effect of duration of exposure to adversity on poorer cognitive functioning in late adulthood. The assessment of cognition and mental health in childhood also allowed adjustment for important covariates and helped to mitigate against reverse directionality, although it does not fully exclude this possibility. The inclusion of two different domains of adversity as well as three different cognitive measures further allowed testing of specificity in the associations.

There are also some limitations that should be considered. For instance, there may be bias from selective drop-out, as previous research has suggested that those who dropped out were more likely to have lower childhood cognitive ability and educational attainment, and worse mental health.[25] This may have led to an underestimation in the association between exposure to

For peer review only - http://bmjopen.bmj.com/site/about/guidelines.xhtml

For peer review only

adversity and cognitive functioning. In addition, certain adversities may not be experienced in the same way across different lifecourse stages. For example, childhood economic adversity was relatively common in this cohort, given they were born in a time of significant economic uncertainty in a post-war Britain,[57] and may be experienced differently compared to younger birth cohorts. There is some evidence suggesting that the severity of adversity should also be considered in the accumulation model, as adverse events that are perceived as life changing may show stronger effect.[58,59] Caution is also needed in assuming that the same adverse experience is being captured across the lifecourse, particularly as certain indicators of adversity (i.e., difficulties with peers) was reported by the teacher. The lack of ethnic diversity in the cohort also limits the generalisability of these findings, particularly as people from ethnic minority backgrounds are more likely to experience adversities [60] and may be disproportionally affected by them. Furthermore, certain adversities may also co-occur as risk clusters or risk chains,[61–63] and adversities occurring at different stages of the lifecourse may also show differential effects. This was not investigated in the current study given our aim of testing the duration of exposure across the lifecourse, However, there is evidence for a recency effect of adversities on mental health, where later life adversities show stronger associations with mental health compared to adversities experienced earlier in the lifecourse.[58] Further research should therefore focus on assessing the severity as well as the timing of adversities, taking into account risk clusters between different adversities, and to investigate other possible mechanisms such as cardiometabolic health when examining the impact of lifecourse adversity on cognitive ageing.

Conclusion

Overall, findings from this study suggest that increased duration of exposure to any adversity – particularly economic adversity – has a small dose-response effect on cognitive state and

For peer review only - http://bmjopen.bmj.com/site/about/guidelines.xhtml

verbal memory in older age. Mental health mediated the dose-response effect of duration of exposure to all types of adversity (any, economic, psychosocial) on cognition, suggesting that routinely assessing and improving mental health in older adults may also help improve cognitive function in older age. Future studies should also account for the severity, timing, and co-occurrence of adversities, as well as investigate other potential mediators such as cardiometabolic health, to better understand pathways to cognitive ageing.

For peer review only

For peer review only - http://bmjopen.bmj.com/site/about/guidelines.xhtml

Funding: This study is funded by the UK Medical Research Council which provides core funding for the NSHD (MC_UU_00019/1). MR additionally reports funding from MRC (MC_UU_00019/3). JMS acknowledges the support of the National Institute for Health Research University College London Hospitals Biomedical Research Centre, Alzheimer's Research UK, Medical Research Council, UK Dementia Research Institute, Alzheimer's Association, and the British Heart Foundation (PG/17/90/33415). The funders had no involvement in study design, data collection, analysis, interpretation or the decision to submit for publication. The views expressed are those of the author(s) and YL serves as guarantor for the contents of this paper.

Declaration of interest: None

Acknowledgements

We are grateful to all study members who took part in the NSHD and to the study team members who helped to collect the data over the last seven decades.

Author contributions

Conceptualisation: YL, MR, PP

Methodology: YL, PP, JS

Funding acquisition: MR, JMS, PP

Formal analysis: YL

Writing – Original draft: YL

Writing – Review and editing: All authors

Data availability

The data that support the findings of this study are available to bona fide researchers upon request to the NSHD Data Sharing Committee. For more information see: http://www.nshd.mrc.ac.uk/data.

For peer review only - http://bmjopen.bmj.com/site/about/guidelines.xhtml

List of Figures

Fig. 1. Sample flow diagram. *ACE-III: Addenbrooke's Cognitive Examination-III*

Fig. 2. Standardised estimates on the association between duration of exposure to adversity and each cognitive function at 69 years.

Fig. 3. Mediation models showing the direct and indirect effects of lifecourse adversity (duration of exposure to any adversity) on each cognitive function via mental health (psychological distress). **p<0.05; ** p<0.01*

Fig. 4. Mediation models showing the direct and indirect pathways from duration of exposure to economic or psychosocial adversity to cognitive function via mental health (psychological distress). **p<0.05; ** p<0.01*

For peer review only

For peer review only - http://bmjopen.bmj.com/site/about/guidelines.xhtml

References

1 Prince MJ, Wu F, Guo Y, *et al.* The burden of disease in older people and implications for health policy and practice. *The Lancet* 2015; **385**: 549–62.

2 Turner RJ, Lloyd DA. Lifetime traumas and mental health: the significance of cumulative adversity. *J Health Soc Behav* 1995; **36**: 360–76.

3 Chen R, Williams DR, Nishimi K, Slopen N, Kubzansky LD, Weuve J. A life course approach to understanding stress exposures and cognitive function among middle-aged and older adults. *Soc Sci Med* 2022; **314**: 115448.

4 Greenfield EA, Moorman SM. Childhood Socioeconomic Status and Later Life Cognition: Evidence From the Wisconsin Longitudinal Study. *J Aging Health* 2019; **31**: 1589–615.

5 Lee Y, Back JH, Kim J, Byeon H. Multiple Socioeconomic Risks and Cognitive Impairment in Older Adults. *Dement Geriatr Cogn Disord* 2010; **29**: 523–9.

6 Lyu J, Burr JA. Socioeconomic Status Across the Life Course and Cognitive Function Among Older Adults: An Examination of the Latency, Pathways, and Accumulation Hypotheses. *J Aging Health* 2016; **28**: 40–67.

7 Shrira A. Greater age-related decline in markers of physical, mental and cognitive health among Israeli older adults exposed to lifetime cumulative adversity. *Aging Ment Health* 2014; **18**: 610–8.

8 McEwen BS, Rasgon NL. The Brain and Body on Stress: Allostatic Load and Mechanisms for Depression and Dementia. Oxford University Press, 2018 https://oxfordmedicine.com/view/10.1093/med/9780190603342.001.0001/med-9780190603342-chapter-2 (accessed Feb 15, 2022).

9 Power C, Kuh D, Morton S. From Developmental Origins of Adult Disease to Life Course Research on Adult Disease and Aging: Insights from Birth Cohort Studies. *Annu Rev Public Health* 2013; **34**: 7–28.

10 Shrira A. Greater age-related decline in markers of physical, mental and cognitive health among Israeli older adults exposed to lifetime cumulative adversity. *Aging Ment Health* 2014; **18**: 610–8.

11 Künzi M, Gheorghe DA, Kliegel M, Ballhausen N, Gallacher J, Bauermeister S. Cumulative life course adversity, mental health, and cognition in the UK biobank. *Sci Rep* 2022; **12**: 14700.

12 Zeki Al Hazzouri A, Elfassy T, Sidney S, Jacobs D, Pérez Stable EJ, Yaffe K. Sustained Economic Hardship and Cognitive Function: The Coronary Artery Risk Development in Young Adults Study. *Am J Prev Med* 2017; **52**: 1–9.

13 Ejlskov L, Bøggild H, Kuh D, Stafford M. Social relationship adversities throughout the lifecourse and risk of loneliness in later life. *Ageing Soc* 2020; **40**: 1718–34.

14 Hatch SL, Mishra G, Hotopf M, Jones PB, Kuh D. Appraisals of stressors and common mental disorder from early to mid-adulthood in the 1946 British birth cohort. *J Affect Disord* 2009; **119**: 66–75.

For peer review only - http://bmjopen.bmj.com/site/about/guidelines.xhtml

15 Shrira A. The effect of lifetime cumulative adversity on change and chronicity in depressive symptoms and quality of life in older adults. *Int Psychogeriatr* 2012; **24**: 1988–97.

16 Liu Y, Hatch SL, Patalay P, Schott JM, Richards M. A lifecourse approach in examining the association between accumulation of adversity and mental health in older adulthood. *J Affect Disord* 2023; **339**: 211–8.

17 Livingston G, Huntley J, Sommerlad A, *et al.* Dementia prevention, intervention, and care: 2020 report of the Lancet Commission. *The Lancet* 2020; **396**: 413–46.

18 John A, Rusted J, Richards M, Gaysina D. Bidirectional relation between affective symptoms and cognitive function from middle to late adulthood: a population-based birth cohort study. *Aging Ment Health* 2021; **25**: 1642–8.

19 Richards M, Hatch SL. A life course approach to the development of mental skills. *J Gerontol B Psychol Sci Soc Sci* 2011; **66 Suppl 1**: i26-35.

20 Cadar D, Kobayashi LK, Steptoe AP, Demakakos P. OP24 The mediating pathways of the association between adverse childhood experiences and cognitive health in later life. *J Epidemiol Community Health* 2020; **74**: A11–2.

21 Verlinden M, Veenstra R, Ghassabian A, *et al.* Executive Functioning and Non-Verbal Intelligence as Predictors of Bullying in Early Elementary School. *J Abnorm Child Psychol* 2014; **42**: 953–66.

22 Zych I, Farrington DP, Llorent VJ, Ribeaud D, Eisner MP. Childhood Risk and Protective Factors as Predictors of Adolescent Bullying Roles. *Int J Bullying Prev* 2021; **3**: 138–46.

23 Lawn RB, Anderson EL, Suderman M, *et al.* Psychosocial adversity and socioeconomic position during childhood and epigenetic age: analysis of two prospective cohort studies. *Hum Mol Genet* 2018; **27**: 1301–8.

24 Sousa ACP de A, Guerra RO, Tu MT, Phillips SP, Guralnik JM, Zunzunegui M-V. Lifecourse Adversity and Physical Performance across Countries among Men and Women Aged 65-74. *PLOS ONE* 2014; **9**: e102299.

25 Kuh D, Wong A, Shah I, *et al.* The MRC National Survey of Health and Development reaches age 70: maintaining participation at older ages in a birth cohort study. *Eur J Epidemiol* 2016; **31**: 1135–47.

26 Goldberg DP, Hillier VF. A scaled version of the General Health Questionnaire. *Psychol Med* 1979; **9**: 139–45.

27 Noone P. Addenbrooke's Cognitive Examination-III. *Occup Med* 2015; **65**: 418–20.

28 James S-N, Davis D, O'Hare C, *et al.* Lifetime affective problems and later-life cognitive state: Over 50 years of follow-up in a British birth cohort study. *J Affect Disord* 2018; **241**: 348–55.

29 Richards M, James S-N, Sizer A, *et al.* Identifying the lifetime cognitive and socioeconomic antecedents of cognitive state: seven decades of follow-up in a British birth cohort study. *BMJ Open* 2019; **9**: e024404.

30 Pigeon DA. Tests used in the 1954 and 1957 surveys. *Home Sch* 1964; : 129–32.

For peer review only - http://bmjopen.bmj.com/site/about/guidelines.xhtml

31 Imai K, Keele L, Tingley D, Yamamoto T. Unpacking the Black Box of Causality: Learning about Causal Mechanisms from Experimental and Observational Studies. *Am Polit Sci Rev* 2011; **105**: 765–89.

32 Imai K, Keele L, Tingley D. A general approach to causal mediation analysis. *Psychol Methods* 2010; **15**: 309–34.

33 Imai K, Keele L, Yamamoto T. Identification, Inference and Sensitivity Analysis for Causal Mediation Effects. *Stat Sci* 2010; **25**: 51–71.

34 Tingley D, Yamamoto T, Hirose K, Keele LJ, Imai K. mediation: R Package for Causal Mediation Analysis. *J Stat Softw* 2014; **59**: 1–38.

35 Buuren SV, Groothuis-oudshoorn K. MICE: Multivariate imputation by chained equations in R. *J Stat Softw* 2011; **45**: 1–67.

36 Kuiper JS, Smidt N, Zuidema SU, Comijs HC, Oude Voshaar RC, Zuidersma M. A longitudinal study of the impact of social network size and loneliness on cognitive performance in depressed older adults. *Aging Ment Health* 2020; **24**: 889–97.

37 Richards M, Wadsworth MEJ. Long term effects of early adversity on cognitive function. *Arch Dis Child* 2004; **89**: 922–7.

38 Staff RT, Chapko D, Hogan MJ, Whalley LJ. Life course socioeconomic status and the decline in information processing speed in late life. *Soc Sci Med* 2016; **151**: 130–8.

39 Ding R, He P. Associations between childhood adversities and late-life cognitive function: Potential mechanisms. *Soc Sci Med* 2021; **291**: 114478.

40 Halpin AB, MacAulay RK, Boeve AR, D'Errico LM, Michaud S. Are Adverse Childhood Experiences Associated with Worse Cognitive Function in Older Adults? *J Int Neuropsychol Soc* 2021; : 1–10.

41 Colman I, Wadsworth MEJ, Croudace TJ, Jones PB. Forty-Year Psychiatric Outcomes Following Assessment for Internalizing Disorder in Adolescence. *Am J Psychiatry* 2007; **164**: 126–33.

42 Copeland WE, Alaie I, Jonsson U, Shanahan L. Associations of Childhood and Adolescent Depression With Adult Psychiatric and Functional Outcomes. *J Am Acad Child Adolesc Psychiatry* 2021; **60**: 604–11.

43 Groot S de, Veldman K, Iii BCA, Bültmann U. Work functioning among young adults: the role of mental health problems from childhood to young adulthood. *Occup Environ Med* 2022; **79**: 217–23.

44 Stansfeld SA, Clark C, Caldwell T, Rodgers B, Power C. Psychosocial work characteristics and anxiety and depressive disorders in midlife: the effects of prior psychological distress. *Occup Environ Med* 2008; **65**: 634–42.

45 Keller J, Gomez R, Williams G, *et al.* HPA axis in major depression: cortisol, clinical symptomatology and genetic variation predict cognition. *Mol Psychiatry* 2017; **22**: 527–36.

46 Wingenfeld K, Wolf OT. HPA Axis Alterations in Mental Disorders: Impact on Memory and its Relevance for Therapeutic Interventions. *CNS Neurosci Ther* 2011; **17**: 714–22.

For peer review only - http://bmjopen.bmj.com/site/about/guidelines.xhtml

47 Sheridan MA, Shi F, Miller AB, Salhi C, McLaughlin KA. Network structure reveals clusters of associations between childhood adversities and development outcomes. *Dev Sci* 2020; **23**: e12934.

48 Mani A, Mullainathan S, Shafir E, Zhao J. Poverty Impedes Cognitive Function. *Science* 2013; **341**: 976–80.

49 Shah AK, Mullainathan S, Shafir E. Some Consequences of Having Too Little. *Science* 2012; **338**: 682–5.

50 Butterworth P, Cherbuin N, Sachdev P, Anstey KJ. The association between financial hardship and amygdala and hippocampal volumes: results from the PATH through life project. *Soc Cogn Affect Neurosci* 2012; **7**: 548–56.

51 Elbejjani M, Fuhrer R, Abrahamowicz M, *et al.* Life-Course Socioeconomic Position and Hippocampal Atrophy in a Prospective Cohort of Older Adults. *Psychosom Med* 2017; **79**: 14–23.

52 Pollitt RA, Rose KM, Kaufman JS. Evaluating the evidence for models of life course socioeconomic factors and cardiovascular outcomes: a systematic review. *BMC Public Health* 2005; **5**: 7.

53 Lyall DM, Celis-Morales CA, Anderson J, *et al.* Associations between single and multiple cardiometabolic diseases and cognitive abilities in 474 129 UK Biobank participants. *Eur Heart J* 2017; **38**: 577–83.

54 Oi K, Haas S. Cardiometabolic Risk and Cognitive Decline: The Role of Socioeconomic Status in Childhood and Adulthood. *J Health Soc Behav* 2019; **60**: 326–43.

55 Prince MJ, Wimo A, Guerchet MM, Ali GC, Wu Y-T, Prina M. World Alzheimer Report 2015 - The Global Impact of Dementia: An analysis of prevalence, incidence, cost and trends. 2015; published online Aug. https://kclpure.kcl.ac.uk/portal/en/publications/world-alzheimer-report-2015--the-global-impact-of-dementia(ae525fda-1938-4892-8daa-a2222a672254).html (accessed May 24, 2022).

56 Trautmann S, Rehm J, Wittchen H-U. The economic costs of mental disorders. *EMBO Rep* 2016; **17**: 1245–9.

57 Short JR. Housing in Britain: The Post-War Experience. London: Routledge, 2021 DOI:10.4324/9781003158066.

58 Hatch SL, Mishra G, Hotopf M, Jones PB, Kuh D. Appraisals of stressors and common mental disorder from early to mid-adulthood in the 1946 British birth cohort. *J Affect Disord* 2009; **119**: 66–75.

59 Power C, Kuh D, Morton S. From Developmental Origins of Adult Disease to Life Course Research on Adult Disease and Aging: Insights from Birth Cohort Studies. *Annu Rev Public Health* 2013; **34**: 7–28.

60 Adjei NK, Schlüter DK, Straatmann VS, *et al.* Impact of poverty and family adversity on adolescent health: a multi-trajectory analysis using the UK Millennium Cohort Study. *Lancet Reg Health - Eur* 2022; **13**: 100279.

61 Kuh D, Ben-Shlomo Y, Lynch J, Hallqvist J, Power C. Life course epidemiology. *J Epidemiol Community Health* 2003; **57**: 778–83.

For peer review only - http://bmjopen.bmj.com/site/about/guidelines.xhtml

62 Rutter M. Psychosocial Adversity: Risk, Resilience & Recovery. *South Afr J Child Adolesc Ment Health* 1995; **7**: 75–88.

63 Rutter M. Resilience concepts and findings: implications for family therapy. *J Fam Ther* 1999; **21**: 119–44.

For peer review only

For peer review only - http://bmjopen.bmj.com/site/about/guidelines.xhtml

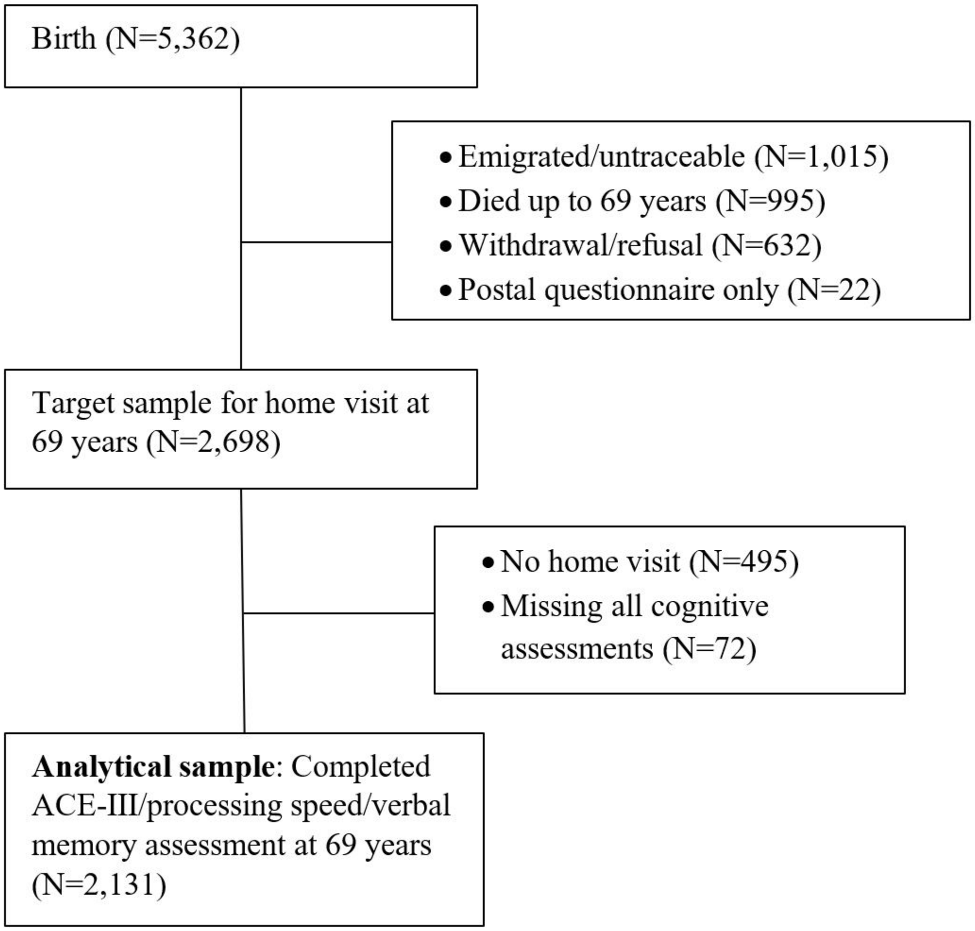

Fig. 1. Sample flow diagram. ACE-III: Addenbrooke's Cognitive Examination-III.

224x220mm (120 x 120 DPI)

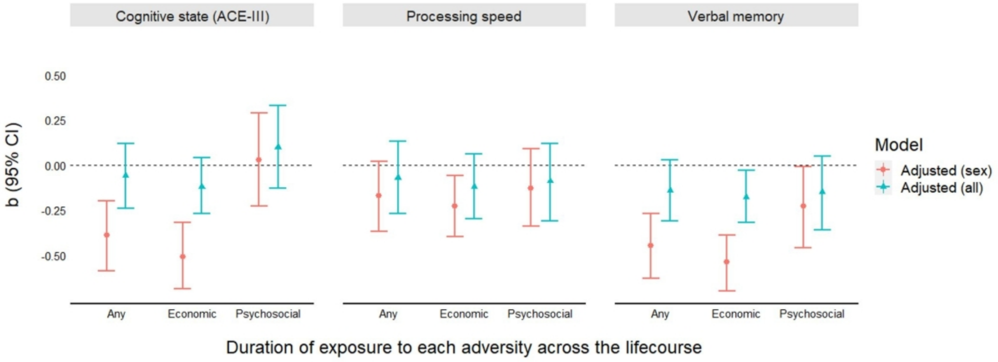

Fig. 2. Standardised estimates on the association between duration of exposure to adversity and each cognitive function at 69 years.

437x168mm (120 x 120 DPI)

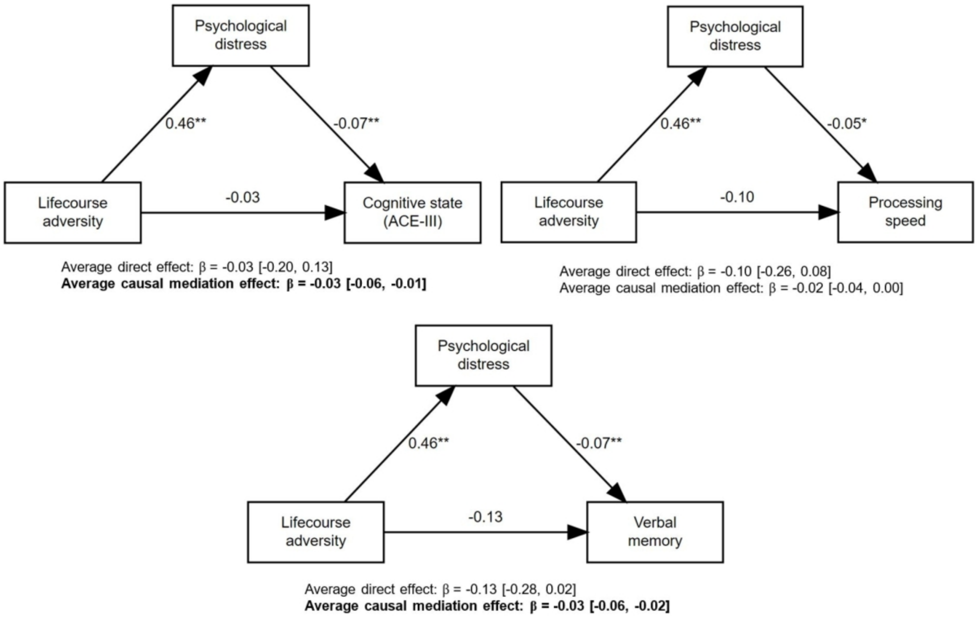

Fig. 3. Mediation models showing the direct and indirect effects of lifecourse adversity (duration of exposure to any adversity) on each cognitive function via mental health (psychological distress). **p<0.05; ** p<0.01*

404x250mm (120 x 120 DPI)

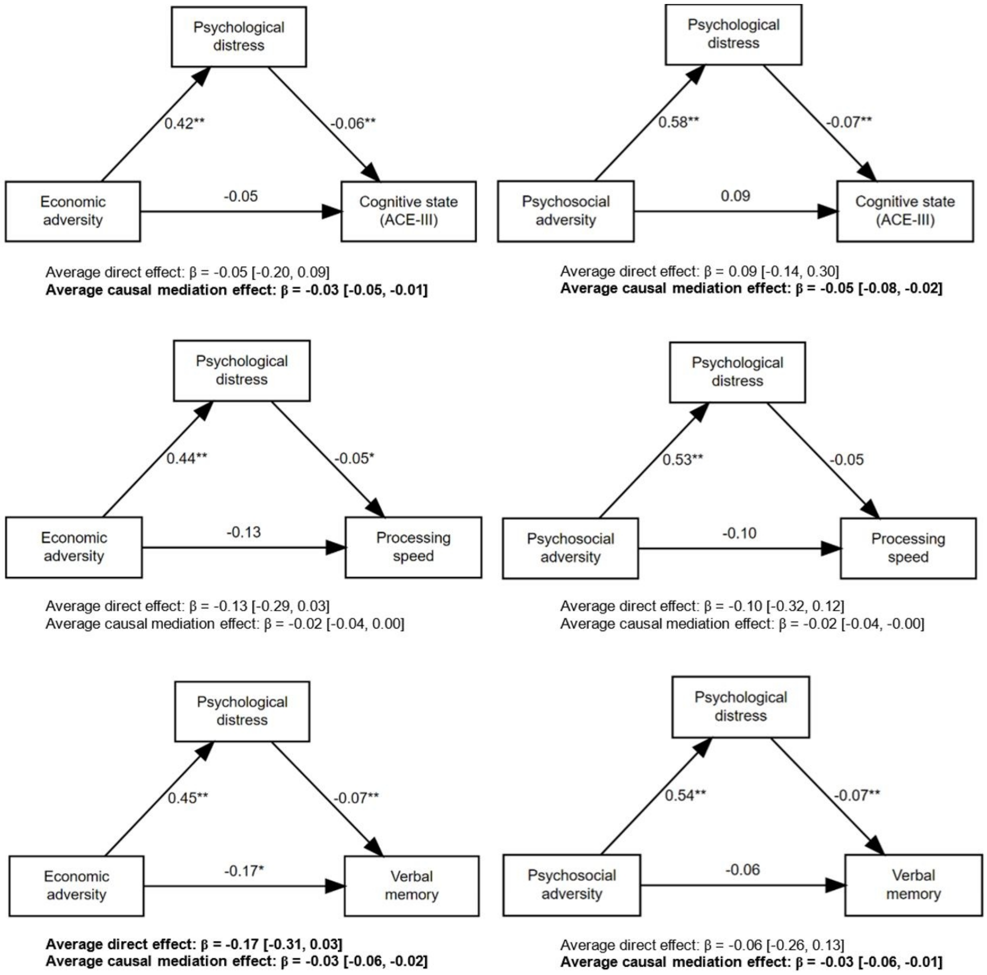

Fig. 4. Mediation models showing the direct and indirect pathways from duration of exposure to economic or psychosocial adversity to cognitive function via mental health (psychological distress). **p<0.05; ** p<0.01*

264x256mm (120 x 120 DPI)

Supplementary materials

Table S1. List of adversity indicators at each stage of the life course.

Adversity variables	N (%)
**Childhood (<17 years)**	
Economic adversity	1353 (63.6%)
Overcrowding	*376 (17.7%)*
Lack of household amenities	*1095 (52.6%)*
Adverse housing condition	*518 (28.9%)*
Paternal occupational status	*167 (8.9%)*
Psychosocial adversity	324 (15.2%)
Separation from mother (>28 days; not due to hospitalisation)	*98 (5.1%)*
Parental divorce	*118 (5.5%)*
Negative peer relationships	*129 (6.8%)*
**Young adulthood (20-36 years)**	
Economic adversity	823 (40.3%)
Overcrowding	*24 (1.3%)*
Lack of household amenities	*185 (9.6%)*
Financial hardships	*323 (20.9%)*
Unemployment	*387 (19.9%)*
Psychosocial adversity	648 (31.4%)
Social isolation	*569 (29.3%)*
Divorce/separation	*112 (5.5%)*
**Mid-adulthood (43-53 years)**	
Economic adversity	993 (47.4%)
Financial hardships	*573 (27.4%)*
Work-related crisis	*358 (17.1%)*
Adverse living conditions	*410 (20.3%)*
Psychosocial adversity	1105 (52.7%)
Social isolation	*148 (7.1%)*
Lack of social support	*207 (9.9%)*
Loss of contact with relatives or friends	*269 (12.8%)*
Difficulties with own children	*563 (29.7%)*
Divorce/separation	*355 (16.9%)*
**Late adulthood (63 years)**	
Economic adversity	316 (16.7%)
Financial hardships	*234 (12.7%)*
Work-related crisis	*113 (6.1%)*
Psychosocial adversity	511 (25.8%)
Social isolation	*50 (2.8%)*
Lack of social support	*12 (0.7%)*
Loss of contact with relatives or friends	*99 (5.4%)*
Difficulties with own children	*217 (13.0%)*
Divorce/separation	*209 (11.5%)*

For peer review only

Causal mediation analysis: Statistical equations

Defining total effect, average direct effect (ADE) and average causal mediation effect (ACME) (adapted from Imai et al., 2011):

Let t = exposure, M = mediator, and Y = outcome. The ACME can be defined using the below formula:

$$\sum\{Y(t, M(t_1)) - Y(t, M(t_0))\}$$

Where ACME equals the difference in outcome due to the change in mediator from the value expected under no exposure ($M(t_0)$) to the value expected under exposure ($M(t_1)$), holding the exposure t at a constant. Fixing the exposure and changing the mediator allows the elimination of other mechanisms and isolates the mediation effect via the mediator.

The ADE can be defined using the following:

$$\sum\{Y(t_1, M(t)) - Y(t_0, M(t))\}$$

Where ADE equals the difference in outcome due to a change in the exposure from not exposed (t_0) to exposed (t_1), holding the mediator at a constant that would be expected under exposure ($M(t)$). This represents the effect of exposure on the outcome that is not hypothesised to be transmitted via the mediator. The total effect equals the sum of the ACME and ADE.

Reference:

Imai K, Keele L, Tingley D, Yamamoto T. Unpacking the Black Box of Causality: Learning about Causal Mechanisms from Experimental and Observational Studies. *Am Polit Sci Rev* 2011; 105: 765–89.

For peer review only - http://bmjopen.bmj.com/site/about/guidelines.xhtml

Table S2. Linear regression models showing the association between duration of exposure to adversity (any, economic, psychosocial) across the lifecourse and cognitive function at 69 years.

Duration of exposure across the lifecourse to:	Cognitive state (ACE-III)			Processing speed			Verbal memory		
	β	95%CI	p-value	β	95%CI	p-value	β	95%CI	p-value
**Any adversity [linear trend]**									
Model 1[a]	**-0.39**	**-0.59; -0.20**	**<0.001**	-0.17	-0.37; 0.02	0.084	**-0.45**	**-0.63; -0.27**	**<0.001**
Model 2[b]	-0.06	-0.24; 0.12	0.510	-0.07	-0.27; 0.13	0.476	-0.14	-0.31; 0.03	0.102
**Economic adversity [linear trend]**									
Model 1[a]	**-0.51**	**-0.69; -0.32**	**<0.001**	**-0.23**	**-0.40; -0.06**	**0.010**	**-0.54**	**-0.70; -0.39**	**<0.001**
Model 2[b]	-0.12	-0.27; 0.04	0.147	-0.12	-0.30; 0.06	0.195	**-0.18**	**-0.32; -0.03**	**0.016**
**Psychosocial adversity [linear trend]**									
Model 1[a]	0.03	-0.23; 0.29	0.834	-0.13	-0.34; 0.09	0.255	**-0.23**	**-0.46; -0.01**	**0.044**
Model 2[b]	0.10	-0.13; 0.33	0.397	-0.09	-0.31; 0.12	0.402	-0.15	-0.36; 0.05	0.143

[a]Adjusted for sex
[b]Adjusted additionally for educational attainment and childhood (cognition and emotional problems) covariates.

Table S3. Linear regression models showing the association between duration of exposure to adversity (any, economic, psychosocial) across the lifecourse and mental health (Psychological distress), and between mental health (psychological distress) and cognitive function at 69 years.

	Cognitive state (ACE-III)			Processing speed			Verbal memory		
	β	95%CI	p-value	β	95%CI	p-value	β	95%CI	p-value
**Any adversity**									
Duration of exposure → Psychological distress	**0.46**	**0.26; 0.66**	**<0.001**	**0.46**	**0.28; 0.63**	**<0.001**	**0.46**	**0.28; 0.64**	**<0.001**
Psychological distress → Cognitive function	**-0.07**	**-0.11; -0.02**	**0.005**	**-0.05**	**-0.10; -0.004**	**0.035**	**-0.07**	**-0.11; -0.03**	**0.001**
**Economic adversity**									
Duration of exposure → Psychological distress	**0.42**	**0.21; 0.63**	**<0.001**	**0.44**	**0.26; 0.61**	**<0.001**	**0.45**	**0.27; 0.64**	**<0.001**
Psychological distress → Cognitive function	**-0.06**	**-0.11; -0.02**	**0.007**	**-0.05**	**-0.10; -0.003**	**0.038**	**-0.07**	**-0.11; -0.03**	**0.001**
**Psychosocial adversity**									
Duration of exposure → Psychological distress	**0.58**	**0.31; 0.84**	**<0.001**	**0.53**	**0.30; 0.76**	**<0.001**	**0.54**	**0.29; 0.80**	**<0.001**
Psychological distress → Cognitive function	**-0.07**	**-0.12; -0.02**	**0.004**	-0.05	-0.10; 0.00	0.051	**-0.07**	**-0.11; -0.03**	**0.001**

All models adjusted for socio-demographic (sex, educational attainment) and childhood (cognition and emotional problems) covariates.

For peer review only - http://bmjopen.bmj.com/site/about/guidelines.xhtml

Table S4. Causal mediation analysis showing the average direct, mediated (via mental health), and total effect of duration of exposure to adversity (any, economic, psychosocial) on cognitive function at 69 years.

	Cognitive state (ACE-III)		Processing speed		Verbal memory	
	β	95%CI	β	95%CI	β	95%CI
**Any adversity**						
ADE	-0.03	-0.20; 0.13	-0.10	-0.26; 0.08	-0.13	-0.28; 0.02
ACME	**-0.03**	**-0.06; -0.01**	-0.02	-0.04, 0.00	**-0.03**	**-0.06; -0.02**
Total effect	-0.07	-0.23; 0.09	-0.12	-0.28; 0.05	**-0.16**	**-0.31; -0.02**
**Economic**						
ADE	-0.05	-0.20; 0.09	-0.13	-0.29; 0.03	**-0.17**	**-0.31; -0.03**
ACME	**-0.03**	**-0.05; -0.01**	-0.02	-0.04; 0.00	**-0.03**	**-0.06; -0.02**
Total effect	-0.08	-0.23; 0.06	-0.14	-0.31; 0.01	**-0.20**	**-0.34; -0.07**
**Psychosocial**						
ADE	0.09	-0.14; 0.30	-0.10	-0.32; 0.12	-0.06	-0.26; 0.13
ACME	**-0.05**	**-0.08; -0.02**	-0.02	-0.04; 0.00	**-0.03**	**-0.06; -0.01**
Total effect	0.04	-0.18; 0.25	-0.11	-0.34; 0.10	-0.10	-0.30; 0.09

Adjusted for socio-demographic (sex, educational attainment) and childhood (cognition and emotional problems) covariates. Abbreviations: ADE: average direct effect; ACME: average causal mediation effect.

Table S5. Linear regression models on the interaction between economic and psychosocial adversity on verbal memory at 69 years.

	Verbal memory		
	**β**	**95%CI**	**p-value**
**Interaction model**			
Economic x psychosocial adversity	-0.79	-3.73; 2.15	0.597

For peer review only - http://bmjopen.bmj.com/site/about/guidelines.xhtml

Sensitivity analyses

Repeating steps from main analysis for a subset of participants with complete data on all three cognitive measures (N=1720)

Table S6. Linear regression models showing the association between duration of exposure to adversity (any, economic, psychosocial) across the lifecourse and cognitive function at 69 years (sensitivity analyses).

Duration of exposure across the lifecourse to:	Cognitive state (ACE-III)			Processing speed			Verbal memory		
	β	95%CI	p-value	β	95%CI	p-value	β	95%CI	p-value
**Any adversity [linear trend]**									
Model 1[a]	**-0.37**	**-0.55; -0.18**	**<0.001**	-0.17	-0.37; 0.04	0.107	**-0.46**	**-0.66; -0.27**	**<0.001**
Model 2[b]	-0.03	-0.19; 0.13	0.716	-0.06	-0.27; 0.15	0.579	-0.15	-0.33; 0.02	0.091

[a]Adjusted for sex

[b]Adjusted additionally for educational attainment and childhood (cognition and emotional problems) covariates.

Table S7. Causal mediation analysis showing the average direct, mediated (via mental health), and total effect of duration of exposure to adversity (any, economic, psychosocial) on cognitive function at 69 years.

	Cognitive state (ACE-III)		Processing speed		Verbal memory	
	β	95%CI	β	95%CI	β	95%CI
**Any adversity**						
ADE	0.01	-0.14; 0.16	0.01	-0.17; 0.20	-0.11	-0.28; 0.05
ACME	**-0.03**	**-0.06; -0.01**	-0.02	-0.05; 0.00	**-0.04**	**-0.06; -0.02**
Total effect	-0.02	-0.18; 0.13	-0.01	-0.20; 0.17	-0.15	-0.31; 0.01

Adjusted for socio-demographic (sex, educational attainment) and childhood (cognition and emotional problems) covariates. Abbreviations: ADE: average direct effect; ACME: average causal mediation effect.

For peer review only - http://bmjopen.bmj.com/site/about/guidelines.xhtml

STROBE Statement—Checklist of items that should be included in reports of ***cohort studies***

	Item No	Recommendation	Page No
**Title and abstract**	1	(*a*) Indicate the study's design with a commonly used term in the title or the abstract<br>(*b*) Provide in the abstract an informative and balanced summary of what was done and what was found	1-3
**Introduction**			
Background/rationale	2	Explain the scientific background and rationale for the investigation being reported	4-5
Objectives	3	State specific objectives, including any prespecified hypotheses	5-6
**Methods**			
Study design	4	Present key elements of study design early in the paper	6
Setting	5	Describe the setting, locations, and relevant dates, including periods of recruitment, exposure, follow-up, and data collection	6
Participants	6	(*a*) Give the eligibility criteria, and the sources and methods of selection of participants. Describe methods of follow-up<br>(*b*) For matched studies, give matching criteria and number of exposed and unexposed	6-7
Variables	7	Clearly define all outcomes, exposures, predictors, potential confounders, and effect modifiers. Give diagnostic criteria, if applicable	7-10
Data sources/ measurement	8*	For each variable of interest, give sources of data and details of methods of assessment (measurement). Describe comparability of assessment methods if there is more than one group	7-10
Bias	9	Describe any efforts to address potential sources of bias	11
Study size	10	Explain how the study size was arrived at	6
Quantitative variables	11	Explain how quantitative variables were handled in the analyses. If applicable, describe which groupings were chosen and why	7-10
Statistical methods	12	(*a*) Describe all statistical methods, including those used to control for confounding<br>(*b*) Describe any methods used to examine subgroups and interactions<br>(*c*) Explain how missing data were addressed<br>(*d*) If applicable, explain how loss to follow-up was addressed<br>(*e*) Describe any sensitivity analyses	10-12
**Results**			
Participants	13*	(a) Report numbers of individuals at each stage of study—eg numbers potentially eligible, examined for eligibility, confirmed eligible, included in the study, completing follow-up, and analysed<br>(b) Give reasons for non-participation at each stage<br>(c) Consider use of a flow diagram	6-7
Descriptive data	14*	(a) Give characteristics of study participants (eg demographic, clinical, social) and information on exposures and potential confounders<br>(b) Indicate number of participants with missing data for each variable of interest<br>(c) Summarise follow-up time (eg, average and total amount)	12-13
Outcome data	15*	Report numbers of outcome events or summary measures over time	13

For peer review only - http://bmjopen.bmj.com/site/about/guidelines.xhtml

Main results	16	(*a*) Give unadjusted estimates and, if applicable, confounder-adjusted estimates and their precision (eg, 95% confidence interval). Make clear which confounders were adjusted for and why they were included<br>(*b*) Report category boundaries when continuous variables were categorized<br>(*c*) If relevant, consider translating estimates of relative risk into absolute risk for a meaningful time period	14-16
Other analyses	17	Report other analyses done—eg analyses of subgroups and interactions, and sensitivity analyses	18
**Discussion**			
Key results	18	Summarise key results with reference to study objectives	18
Limitations	19	Discuss limitations of the study, taking into account sources of potential bias or imprecision. Discuss both direction and magnitude of any potential bias	21-22
Interpretation	20	Give a cautious overall interpretation of results considering objectives, limitations, multiplicity of analyses, results from similar studies, and other relevant evidence	20-21
Generalisability	21	Discuss the generalisability (external validity) of the study results	21-22
**Other information**			
Funding	22	Give the source of funding and the role of the funders for the present study and, if applicable, for the original study on which the present article is based	23

*Give information separately for exposed and unexposed groups.

Note: An Explanation and Elaboration article discusses each checklist item and gives methodological background and published examples of transparent reporting. The STROBE checklist is best used in conjunction with this article (freely available on the Web sites of PLoS Medicine at http://www.plosmedicine.org/, Annals of Internal Medicine at http://www.annals.org/, and Epidemiology at http://www.epidem.com/). Information on the STROBE Initiative is available at http://www.strobe-statement.org.

For peer review only
